# Nitrogen in Supramolecular Chemistry: Understanding the Pervasive Role as Acceptor of Halogen, Chalcogen, Pnictogen and Tetrel Bonds

**DOI:** 10.1021/jacsau.6c00809

**Published:** 2026-09-01

**Authors:** Harry. W. Nash, Elna Pidcock, Joanna S. Stevens, J. Grant Hill, Lee Brammer

**Affiliations:** † Chemistry Division, MPS School, 7315 University of Sheffield, Sheffield S3 7HF, U.K.; ‡ The Cambridge Crystallographic Data Centre, 12 Union Road, Cambridge CB2 1EZ, U.K.

**Keywords:** nitrogen Lewis bases, halogen bonding, chalcogen bonding, pnictogen bonding, tetrel bonding

## Abstract

Nitrogen-centered Lewis bases are well established as nucleophiles in synthetic chemistry, as ligands in coordination chemistry and as pervasive components of hydrogen bonding in chemistry and structural biology. Their role as acceptors of halogen, chalcogen, pnictogen and tetrel bonds, collectively σ-hole interactions, is also well established experimentally, but only selectively investigated through exploration of geometric and energetic trends. Here we report a comprehensive study, in which we identify the prevalence of this class of interactions and analyze experimental geometric trends from crystallographic data. These analyses are supported by high-accuracy coupled-cluster quantum chemical calculations of interaction energies and use of symmetry-adapted perturbation theory (SAPT) to examine the underlying energetic contributions. Our focus is on comparison, across the four families of σ-hole interactions, of the behavior for N_sp^3^_ vs N_sp^2^_ vs N_sp_ hybridization at the nitrogen atom bearing the lone pair. Halogen and chalcogen bonds are more abundant than pnictogen and tetrel bonds, whereas interaction prevalence tends to be greatest for the heavier congeners in each σ-hole donor group (i.e., I, Te, Sb/Bi, Sn/Pb). The relative prevalence of tetrel bonds compared to halogen bonds increases from N_sp^3^_ to N_sp^2^_ to N_sp_ acceptors, highlighting the importance of steric demands. The heavier congeners among the σ-hole donors generally form interactions with shorter normalized interaction distances, although this is mitigated by sterics, most prominently for tetrel bonds and for N_sp^3^_ Lewis bases. Preference for linearity with respect to the σ-hole angle and the angle at the nitrogen lone pair follow the trend N_sp^3^_ > N_sp^2^_ > N_sp_ also as a consequence of steric demands. More generally, SAPT analysis of interaction energies clearly indicates the prominent influence of sterics, through exchange repulsion, on angular preferences of all interactions. The broader implications of these comprehensive results in areas such as supramolecular, materials and medicinal chemistry are considered.

## Graphical abstract

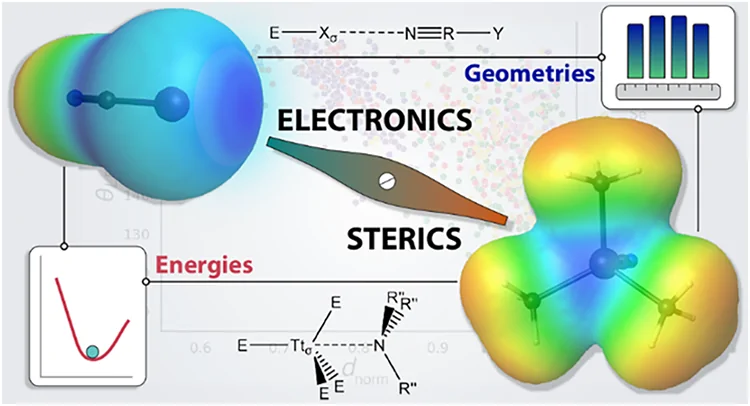

## Introduction

Lewis acid-Lewis base interactions enable both intra- and intermolecular bonding and provide understanding and control of reactions involving engagement of nucleophiles with electrophiles. Among the most pervasive of Lewis bases are molecules that exhibit this basicity through a lone-pair associated with a nitrogen atom,^1–3^ encompassing a large variety of organic functional groups in which the nitrogen atom can be part of either an acyclic or cyclic moiety. Their Lewis acidic partners may be, for example, electrophilic groups in synthetic chemistry,^4,5^ metal ions in coordination chemistry^6–8^ or hydrogen bond donors in supramolecular chemistry^9^ and structural biology.^10^ Over the past 25 years there has been a rapid expansion in interest and investigations of a class of directional noncovalent, predominantly intermolecular, interactions now commonly classified as σ-hole interactions.^11,12^ These include halogen bonds,^13^ chalcogen bonds,^14^ pnictogen bonds^15^ and tetrel bonds^16^ in which the Lewis acidic component, known as the σ-hole donor (*viz*. halogen-bond donor, etc.), is an element in Group 17, 16, 15 or 14, respectively (Figure 1). Other σ-hole donors are known,^17,18^ but our focus in this study is on the aforementioned classes, which are the most prominently studied experimentally. The breadth of applications of these interactions has developed markedly over the past decade or more for halogen bonds^13,19–22^ and chalcogen bonds,^14,23–25^ but understanding and exploitation of other σ-hole interactions is at a much earlier stage of development. Areas of interest include supramolecular chemistry, synthetic chemistry and catalysis, materials chemistry and pharmaceuticals. Pervasive and prominent among these applications of σ-hole interactions is the involvement of nitrogen atoms in the σ-hole acceptor (i.e., Lewis basic) role.

**Figure 1:**
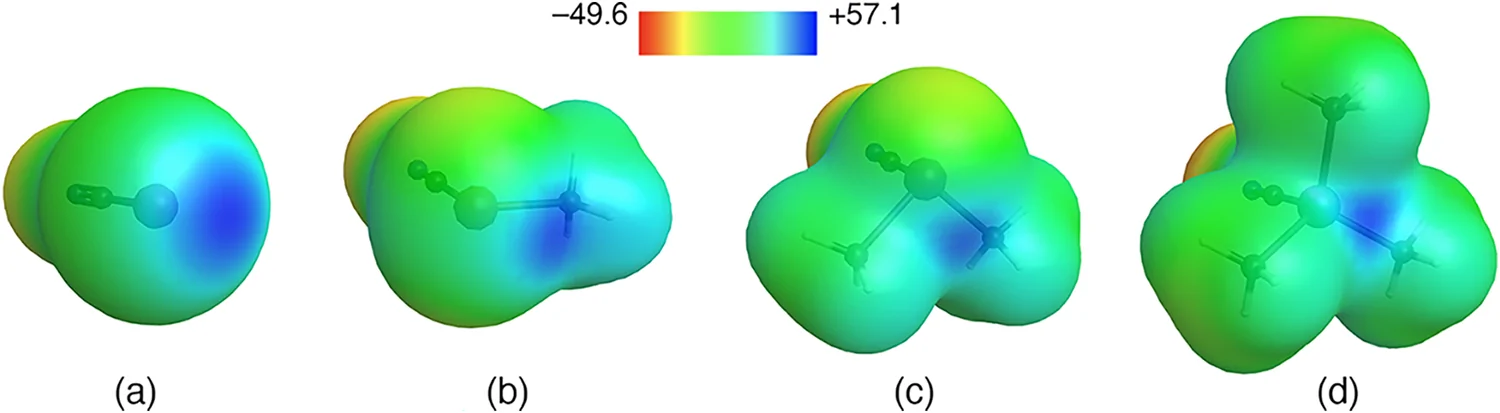
Molecular electrostatic surface potential (kcal mol^–1^) plotted on the 0.002 electrons bohr^–3^ isosurface, calculated at the M06–2X/DZVP level of theory, showing the region of positive electrostatic potential (dark blue) present at the σ-hole, which lies trans to the most electron-withdrawing substituent (CN) for (a) halogen-bond-donor ICN, (b) chalcogen-bond donor Te­(CN)­Me, (c) pnictogen-bond donor Sb­(CN)­Me_2_, (d) tetrel-bond donor Sn­(CN)­Me_3_. A single color range is used for all four molecules.

There have been several models proposed for this type of noncovalent interaction, including in terms of Mulliken complexes involving lone-pair charge transfer to the σ* orbital associated with the covalently bound Lewis acidic atom^26,27^ (e.g., the halogen in a halogen bond). Indeed, most early descriptions followed this bonding model.^17,28^ The σ-hole model was introduced to explain halogen bonding and other similar interactions in electrostatic terms through consideration of the stationary points seen on molecular electrostatic surface potentials (MESPs).^29^ This model has since been revisited^30^ and efforts to address common misconceptions have been presented.^31^ There has also recently been growing understanding of the contributions of energetic components such as dispersion, induction/polarization and quantum mechanical exchange to the overall interaction energy and the observed interaction geometry.^32–34^ The σ-hole is so designated because of the region of reduced electron density situated along the extension of a covalent σ-bond, which is associated with the anisotropic charge distribution around an atom that results from formation of the σ-bond and is accentuated where the bond is formed to an electron withdrawing group.^29^ The σ-hole typically results in a region of positive electrostatic potential (Figure 1) that can form attractive electrostatic interactions with Lewis bases such as anions, lone-pair-containing groups, and π-systems.^29–31,35^ The location of the σ-hole and its associated positive potential, colinear with the σ-bond, leads to a directional, tunable interaction that can be exploited experimentally.^36^

The Cambridge Structural Database (CSD),^37^ with its ever-growing repository of curated crystallographic data (currently >1.4 M crystal structures), provides a platform for investigation of geometric trends in intermolecular interactions. Previous studies of this type on hydrogen bonds or classes of σ-hole interactions have typically focused on the interaction donor (Lewis acid), although occasionally the interaction acceptor (Lewis base) has been investigated in more detail.^38,39^ In the present study we have retrieved and analyzed geometric information from the CSD for halogen, chalcogen, pnictogen and tetrel bonds involving nitrogen Lewis bases as the σ-hole acceptor. The study has enabled comparison of these four classes of σ-hole interactions with common acceptor groups, including subdivision by σ-hole donor atoms within Groups 14–17 and investigation of substituent effects. Alongside these comparisons we have been able to assess the role of nitrogen atom in the acceptors in terms of hybridization at the nitrogen atom. Here we might expect the trend in lone-pair availability (i.e., radial extension) to be N_sp^3^_ > N_sp^3^_ > N_sp_, whereas the trend in lone-pair accessibility (for steric reasons) should be N_sp^3^_ < N_sp^3^_ < N_sp_, giving rise to competing effects on σ-hole interaction formation. Where possible, we have also considered the effect of different nitrogen functional groups. We anticipate that the behavior determined from these studies will be of value in considering more broadly their role as Lewis bases. We have excluded from consideration the lightest congeners from each of the σ-hole donor groups (*viz*. C, N, O and F), which have smaller radii and higher charge density than their heavier congeners, resulting in lower polarizability and limited capability to form σ-hole interactions^40^ and correspondingly limited applicability. The heavier radioactive elements polonium and astatine do not currently feature in the CSD. Consequently only elements from Periods 3–5 have been considered for halogen (X) and chalcogen (Ch) bonding and from Periods 3–6 for pnictogen (Pn) and tetrel (Tt) bonding. Each class of σ-hole donors is also only considered in terms of their saturated valence, i.e., only monovalent halogens, divalent chalcogens, *etc*. (see Figure S1).

Specifically, we have examined trends in the interaction distance normalized with respect to the sum of van der Waals (vdW) radii of the donor and acceptor

$$d_{\mathrm{norm}}=\frac{d}{r_{\sigma }+r_{N}}$$

where *d*_norm_ is the normalized interaction distance,^41^ *d* is the measured contact distance, *r*_σ_ is the vdW radius of the σ-hole donor and *r*_*N*_ is the vdW radius of nitrogen (as the σ-hole acceptor). The directionality with respect to σ-hole of the donor and acceptor atoms has also been examined with the changing nature of the nitrogen Lewis base. The σ-hole angle (θ_σ_) and lone-pair angle (θ_LP_), as defined in Figures 2 and 3, respectively, have a maximum value of 180° when the extension of the σ-bond formed by the σ-hole donor aligns with the direction of the lone pair of the (nitrogen) σ-hole acceptor, which would be considered to be the idealized linear geometry for the σ-hole interaction. Interactions that were so short as to be classified in the CSD as single bonds (i.e., hypervalency at the σ-hole donor) have not been included in our analyses.

**Figure 2:**
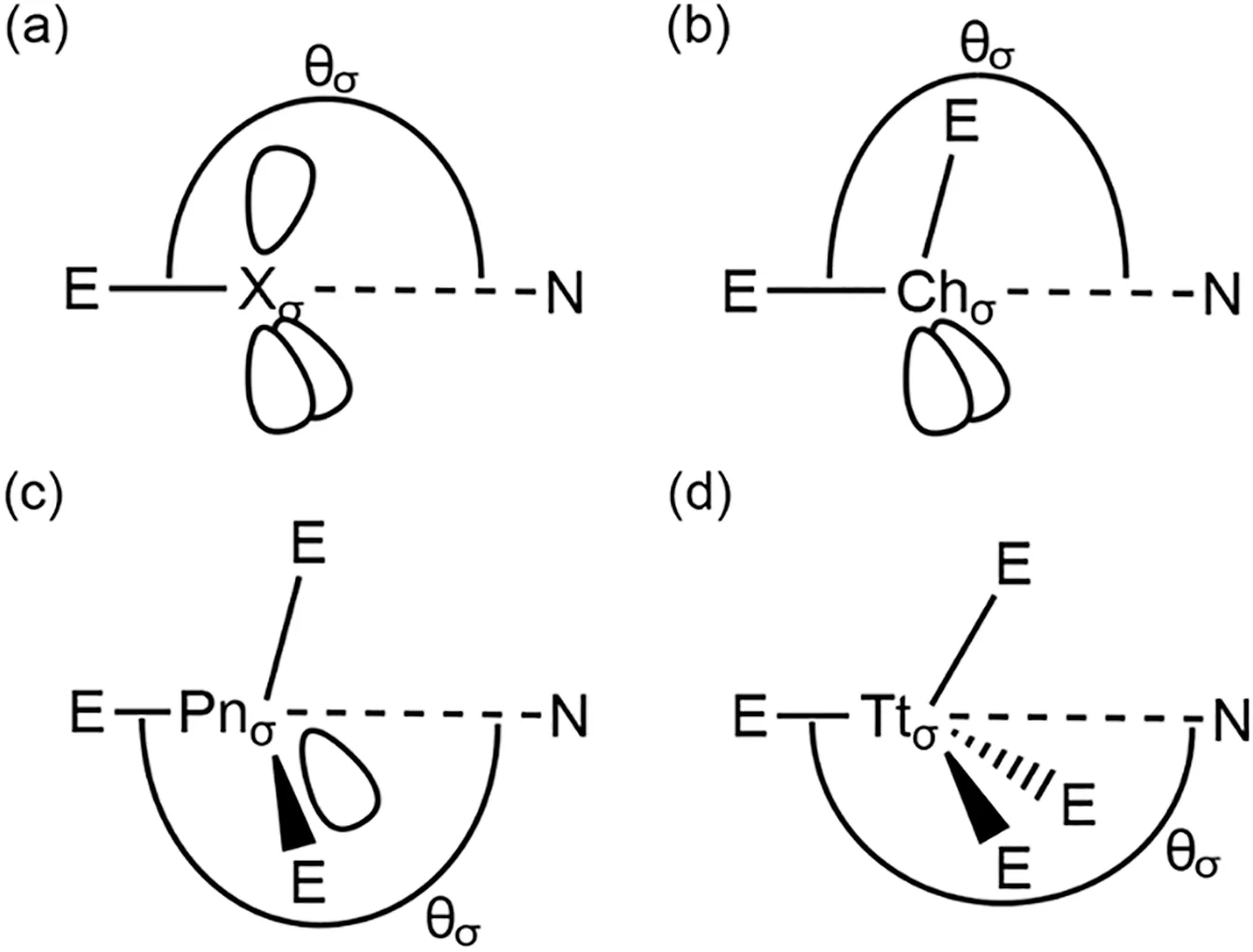
σ-Hole angle, θ_σ_, describes directionality of the σ-hole interaction with respect to the σ-hole donor: (a) halogen bonds, X_σ_ = Cl, Br, I; (b) chalcogen bonds, Ch_σ_ = S, Se, Te; (c) pnictogen bonds, Pn_σ_ = P, As, Sb, Bi and (d) tetrel bonds, Tt_σ_ = Si, Ge, Sn, Pb. Where more than one angle can be defined, the largest of these angles is assigned as the σ-hole angle. Substituent groups in this study were restricted to σ-bonded atoms, E = C, Si, Ge, N, P, As, Sb, O, S, Se, Te, F, Cl, Br, I.

**Figure 3:**
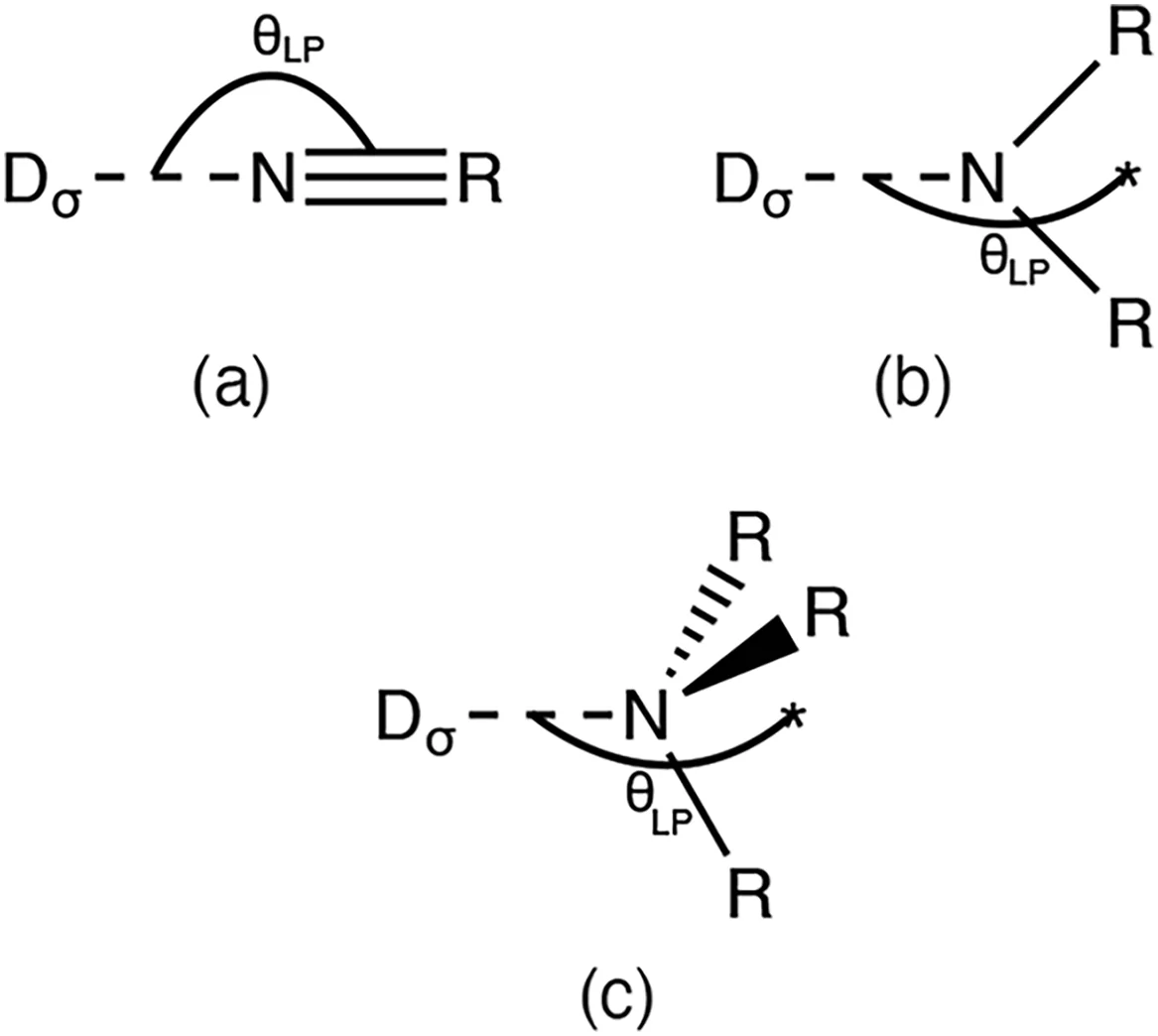
Lone-pair angle, θ_LP_, describes directionality of the σ-hole interaction with respect to the σ-hole acceptor. Defined for the different nitrogen hybridization states of the σ-hole acceptor: (a) N_sp_; (b) N_sp^2^_; (c) N_sp^3^_. D_σ_ is the σ-hole donor (X_σ_, Ch_σ_ Pn_σ_ or Tt_σ_) and the * represents a centroid between the multiple substituents on the sp^2^- and sp^3^-hybridized nitrogen atoms. R = H, B, C, N, O, F, Si, P, S, Cl, Se, Br, Te, I, for (a) and (b) and R = any nonmetal for (c).

The analysis of geometric trends is complemented by high-accuracy ab initio calculations of geometries and σ-hole interaction energies for a series of model systems that reflect the distribution of σ-hole donors and acceptors represented in the CSD studies. These calculations align well with the trends discerned from the experimental data and, notably, through the analysis of underlying energetic contributions provided by symmetry-adapted perturbation theory (SAPT), shed light on the prominent impact of exchange repulsion (viz. sterics) of angular preferences for σ-hole interactions.

## Geometric Trends

### Abundance and Prevalence of σ-Hole Interactions Involving Nitrogen Acceptors

The CSD searches identified 56,797 crystal structures that contain at least one potential σ-hole donor of those under consideration, namely monovalent halogen (Cl–I), divalent chalcogen (S–Te), trivalent pnictogen (P–Bi) or tetravalent tetrel (Si–Pb), along with a nitrogen Lewis base as a potential σ-hole acceptor. Of these crystal structures, 9,689 contained one or more donor–acceptor contacts that met our geometric criteria for consideration as a σ-hole interaction, providing 16,356 σ-hole interactions for further analysis. sp^3^-Hybridized σ-hole acceptors (N_sp^3^_) accounted for 927 interactions (5.67%), N_sp^2^_ for 11,556 interactions (70.65%) and N_sp_ for 3873 interactions (23.68%). Halogen bonds and chalcogen bonds are considerably more abundant than pnictogen and tetrel bonds (Figure 4a), which reflects, in part, the overall abundance in crystal structures of the σ-hole donor elements in their saturated valence.^42^ This is also consistent with levels of research activity to date involving each of these four classes of σ-hole interactions.^12^ Assessing the prevalence of noncovalent interactions is common in the hydrogen bond literature^43^ and serves as an indicator of how likely such interactions are to form and thus a potential guide to efficacy in applications.^44,45^ Our approach follows the structural probability approach of Allen et al.^43^ by determining the number of crystal structures that contain the appropriate σ-hole donor and acceptor moieties to form an interaction (*S*_poss_) and the number of crystal structures in which that interaction occurs within our geometric criteria (*S*_obs_), giving a prevalence (or probability of formation) as *P*_S_(%) = 100 × *S*_obs_/*S*_poss_. It can be seen that prevalence increases for the heavier congeners within each σ-hole donor group (Figure 4b). The relationship of these prevalences to interaction geometries and energies is explored in later sections. Upon examination of the prevalences as a function of hybridization at the σ-hole acceptor nitrogen atom, it becomes apparent that for pnictogen and tetrel bonds the prevalence is high for N_sp_ but decreases for N_sp^2^_ and markedly for N_sp^3^_ (see Tables S1–S5 and Figures S2–S5). This trend is less well pronounced for chalcogen bonds and particularly so for halogen bonds, suggesting an important role for steric interactions arising from increased numbers of substituents at either the σ-hole donor or acceptor atom. Geometric trends across all σ-hole donors involving the three different hybridizations for nitrogen σ-hole acceptors are examined in the following Sections to establish the effects of changes in Lewis basicity at nitrogen and the steric effect of both donor and acceptor groups.

**Figure 4:**
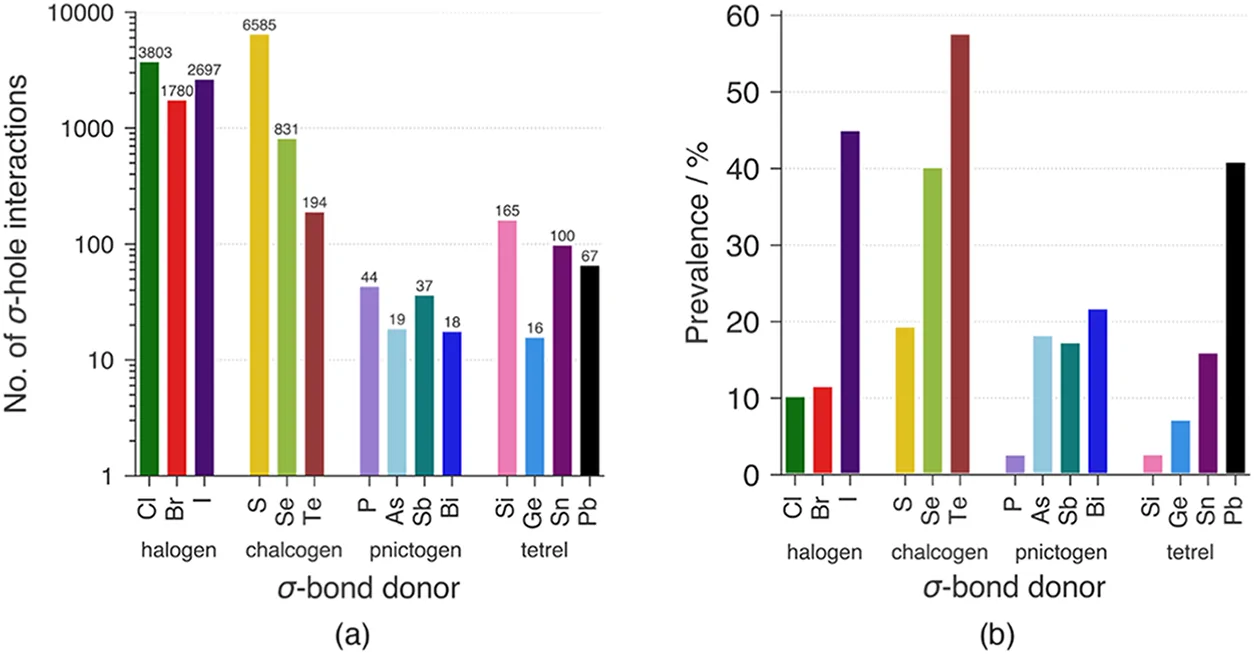
(a) Number of σ-hole interactions (logarithmic scale) for each σ-hole donor group. (b) Prevalence of crystal structures with a σ-hole interaction as proportion of crystal structures that contain the necessary moieties to form such an interaction. Data are shown for interactions involving all nitrogen σ-hole acceptor groups.

### σ-Hole Interactions with N_sp^3^_ Acceptor Groups

For interactions involving N_sp^3^_ acceptor groups, the normalized contact distance, *d*_norm_, is plotted as a function of σ-hole interaction directionality at the σ-hole donor (σ-hole angle, θ_σ_) and acceptor (lone-pair angle, θ_LP_) in Figure 5. These data are further broken down into halogen, chalcogen, pnictogen and tetrel bonds in Figures S6–S9. A strong preference for linearity at the σ-hole donor, wherein θ_σ_ ≥ 160° for most interactions with *d*_norm_ < 1.0, is accentuated further for the shortest interactions. These shorter interactions are dominated by halogen bonds and, in particular, by those involving iodine or bromine as the halogen-bond donor atom, X_σ_. There is a preference for alignment of the interaction with the N_sp^3^_ lone pair of the σ-hole acceptor as shown by θ_LP_ angles approaching 180°, with increased tendency for interactions with shorter *d*_norm_ (Figure 5b). These observations are consistent with there being an important steric role in defining interaction geometry and, by implication, interaction strength (*vide infra*). The N_sp^3^_ acceptor groups are the most sterically encumbered of the different nitrogen hybridization states that we have examined and the steric effect on the interaction geometry is mitigated to the greatest extent by the least sterically encumbered σ-hole donor groups, namely the (monovalent) halogen-bond donors. The mean geometric data presented by σ-hole donor atom type (Table 1), suggest a general trend of shorter interaction distances (*d*_norm_) with geometries closer to linearity (θ_σ_ and θ_LP_) for the heavier elements in each group, whereas the lightest elements exhibit mostly longer *d*_norm_ with very limited preference for larger θ_σ_ and θ_LP_ angles. This is consistent with established trends in polarizability and electronegativity that give rise to more prominent σ-holes both in terms of depletion of electron density and magnitude of positive MESPs for the heavier elements.^30,46^ The very shortest interactions (*d*_norm_ < 0.7) are ones in which the description of bonding begins to move from a σ-hole interaction, commonly described as a noncovalent interaction, to one which approaches hypervalent bonding at the σ-hole donor atom. It has been noted in many previous reports that there exists, in principle, a continuum of interactions between weak (long) noncovalent σ-hole interactions of the type we are concerned with herein and hypervalent *p*-block chemistry. This is eloquently discussed with some historical perspective by Crabtree.^47^

**Figure 5:**
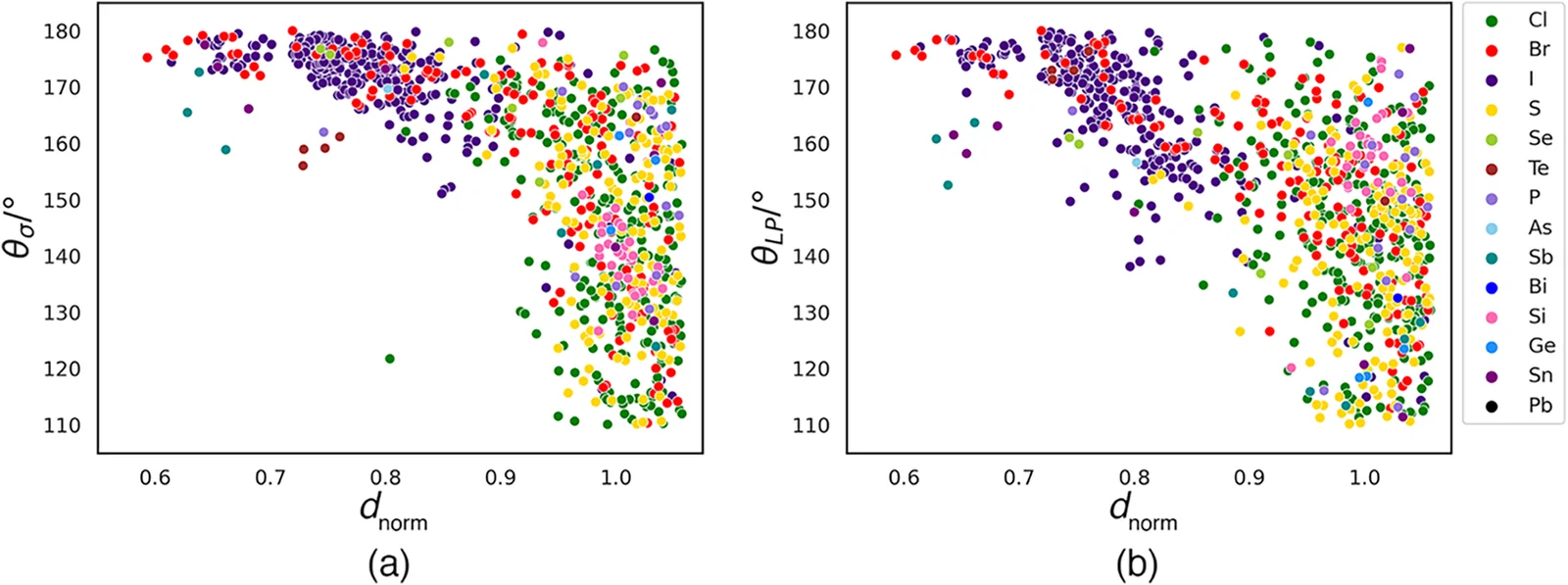
Distribution of geometries for σ-hole interactions involving N_sp^3^_ acceptor groups. (a) Normalized D_σ_···N distance (*d*_norm_) vs E–D_σ_···N σ-hole angle, θ_σ_. (b) Normalized D_σ_···N distance vs N_sp^3^_ lone-pair angle, θ_LP_. Data points are color-coded by σ-hole donor atom, D_σ_. Substituent E is as defined in Figure 2.

**Table 1:** Mean Geometries for σ-Hole Interactions Involving N_sp^3^_ Acceptor Groups^a^

donor–acceptor (D_σ_···N)	mean *d*_norm_	mean D_σ_···N distance (Å)	mean θ_σ_ angle (°)	mean θ_LP_ angle (°)	number of observations (*N*_obs_)
Cl···N_sp^3^_	0.991	3.449	144	145	263
Br···N_sp^3^_	0.918	3.232	157	154	153
I···N_sp^3^_	0.791	2.927	170	165	291
S···N_sp^3^_	0.996	3.535	147	141	141
Se···N_sp^3^_	0.886	3.084	170	149	7
Te···N_sp^3^_	0.796	2.907	160	168	5
P···N_sp^3^_	1.001	3.562	154	149	15
Sb···N_sp^3^_	0.854	3.527	157	136	8
Si···N_sp^3^_	1.004	3.865	140	157	30
Sn···N_sp^3^_	0.860	3.510	161	150	8

a Interactions with fewer than five observations for a given σ-hole donor are not shown (data provided in Table S6).

Shorter interactions typically involve bicyclic amines, such as 1,4-diazabicyclooctane (DABCO) or quinuclidine, which are strongly Lewis basic and less sterically encumbered than most N_sp^3^_ σ-hole acceptor groups, as illustrated in Figure 6. The influence of electron-withdrawing substituents (E) in shortening E–D_σ_···N distances is also apparent from these examples and is examined more generally in a later Section.

**Figure 6:**
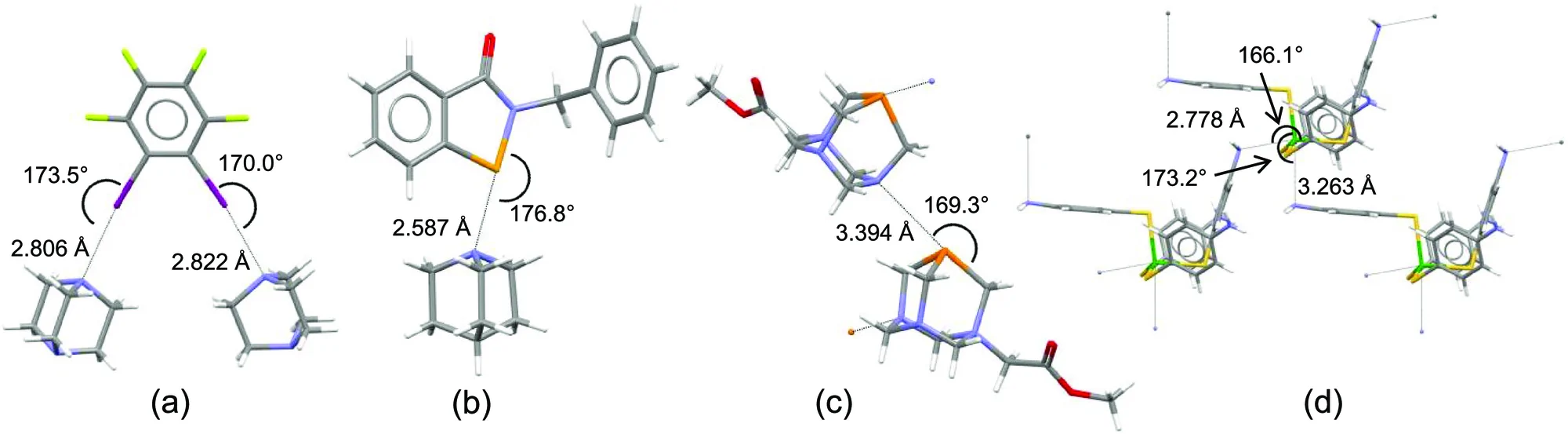
Crystal structures illustrating σ-hole interactions with short D_σ_···N_sp^3^_ distances: (a) C–I···N halogen bonding (*d*_norm_ = 0.76, 0.76) in (DABCO)_2_(1,2-diiodotetrafluorobenzene) (CSD refcode SUMZEI).^48^ (b) N–Se···N chalcogen bonding (*d*_norm_ = 0.74) in a benzisoselenazolinone-quinuclidine cocrystal (CSD refcode GOBYOO).^49^ (c) C–P···N pnictogen bonding (*d*_norm_ = 0.95) in 1-(2-methoxy-2-oxoethyl)-3,5-diaza-1-azonia-7-phosphatricyclo­[3.3.1.1^3,7^]­decane bromide hydrate (CSD refcode DIBWOC; for clarity, only the cation is shown).^50^ (d) S–Sn···N tetrel bonding (*d*_norm_ = 0.68, 0.80) in tetrakis­(4-aminobenzenethiolato)tin (CSD refcode FEYZIT).^51^

### σ-Hole Interactions with N_sp^2^_ Acceptor Groups

By far the most numerous of σ-hole interactions involving nitrogen acceptor groups are those with sp^2^-hybridized nitrogen Lewis bases. Similar geometric trends to those for N_sp^3^_ acceptor groups are observed with an increasing preference for geometries closer to linearity at the σ-hole donor and acceptor (angles θ_σ_ and θ_LP_, respectively) for shorter *d*_norm_ (Figure 7). Illustrative examples of E–D_σ_···N_sp^2^_ bonding are shown in Figure 8. A preference for linearity for interactions involving all σ-hole donors studied is suggested, where sufficient data to discern a trend are available (Table 2, Figures S10–S13), albeit a less pronounced preference than for interactions involving N_sp^3^_ acceptor groups. Halogen bonds with *d*_norm_ < 0.7 are dominated by D_σ_ = I, although there are examples of Br and even Cl with such geometries. Upon examination of the substituent (E) to which the halogen (X_σ_) is σ-bonded it is apparent that these shorter, and presumed stronger, halogen bonds are generated by dihalogens (i.e., X–X_σ_···N) or are N–X_σ_···N interactions (Figure S14). Among chalcogen bonds, those involving sulfur are most numerous overall but those with shorter *d*_norm_ are dominated by Ch_σ_ = Se or Te. It is apparent that, in addition to the general trend of close-to-linear interactions (θ_σ_ > 160°), there are also prominent clusters of examples that exhibit *d*_norm_ < 0.8 in conjunction with θ_σ_ < 160°, notably for Ch_σ_ = Te and even Ch_σ_ = S (Figures 7, S11, S16a). These are associated with cyclic chalcazoles, which exhibit a prominent σ-hole along the extension of the N–Ch bond, leading to strong chalcogen bonds,^49,52,53^ but which require some deviation from linearity of the interaction in order to accommodate molecular dimers for which chalcazole self-association is via a pair of inversion-related N–Ch···N chalcogen bonds (e.g., Figure 8b). We have verified this with high-accuracy coupled-cluster calculations of the intermolecular interaction energy for a simple tellurazole dimer (Figure S17), showing that formation of the second chalcogen bond more than compensates for any required deviation from linearity of θ_σ_ angle(s) required to accommodate this arrangement. Such compounds have been studied in the context of applications or properties such as nonlinear optics,^53^ charge-transfer^54,55^ and mechanism of drug action.^56^ Pnictogen bond geometries suggest some preference for larger θ_σ_ and θ_LP_ angles for interactions with shorter *d*_norm_, albeit with few angles exceeding 170°. The shortest interactions involve Pn_σ_ = Sb. For tetrel bonds, in the absence of those interactions involving Tt_σ_ = Pb, there is a clear increasing preference for linearity (θ_σ_ and θ_LP_ angles tending toward 180°) for the shorter interactions. E–Pb···N_sp^2^_ interactions, although typically short, however, exhibit θ_σ_ angles approximately in the 135–170° range with a very wide range of θ_LP_ angles (approximately 110–170°). These geometries arise from the dominance of hemidirected Pb­(II) complexes (30 of the 31 four-coordinate compounds identified in this study) rather than tetrahedral Pb­(IV) complexes. The former, although potentially able to provide a less sterically crowded environment to enable tetrel bonding, are often encircled by large flat ligands leading to intermolecular association via π-stacking interactions. The association via π-stacking makes the Pb­(II) centers less sterically accessible. In such cases E–Pb···N_sp^2^_ interactions that meet our geometric criteria are often distorted substantially away from linearity (particularly θ_LP_ angle) to accommodate the π-stacking (Figure S18).^57^ These hemidirected complexes can adopt close to linear angles (θ_σ_ and θ_LP_), however, where access to the Pb­(II) site is not as perturbed by intermolecular π-stacking (Figure S19).^58^

**Table 2:** Mean Geometries for σ-Hole Interactions Involving N_sp^2^_ Acceptor Groups^a^

donor–acceptor (D_σ_···N)	mean *d*_norm_	mean D_σ_···N distance (Å)	mean θ_σ_ angle (°)	mean θ_LP_ angle (°)	number of observations (*N*_obs_)
Cl···N_sp^2^_	0.976	3.379	149	140	2764
Br···N_sp^2^_	0.934	3.288	158	146	1172
I···N_sp^2^_	0.815	3.017	170	161	2066
S···N_sp^2^_	0.955	3.392	152	139	4545
Se···N_sp^2^_	0.890	3.097	159	145	586
Te···N_sp^2^_	0.841	3.071	160	150	171
P···N_sp^2^_	1.012	3.603	155	138	12
As···N_sp^2^_	0.943	3.337	159	146	8
Sb···N_sp^2^_	0.812	3.352	162	147	23
Bi···N_sp^2^_	0.883	3.707	162	147	9
Si···N_sp^2^_	1.005	3.868	145	153	70
Sn···N_sp^2^_	0.790	3.225	162	158	66
Pb···N_sp^2^_	0.766	3.262	153	134	63

a Interactions with fewer than five observations for a given σ-hole donor are not shown (data provided in Table S7).

**Figure 7:**
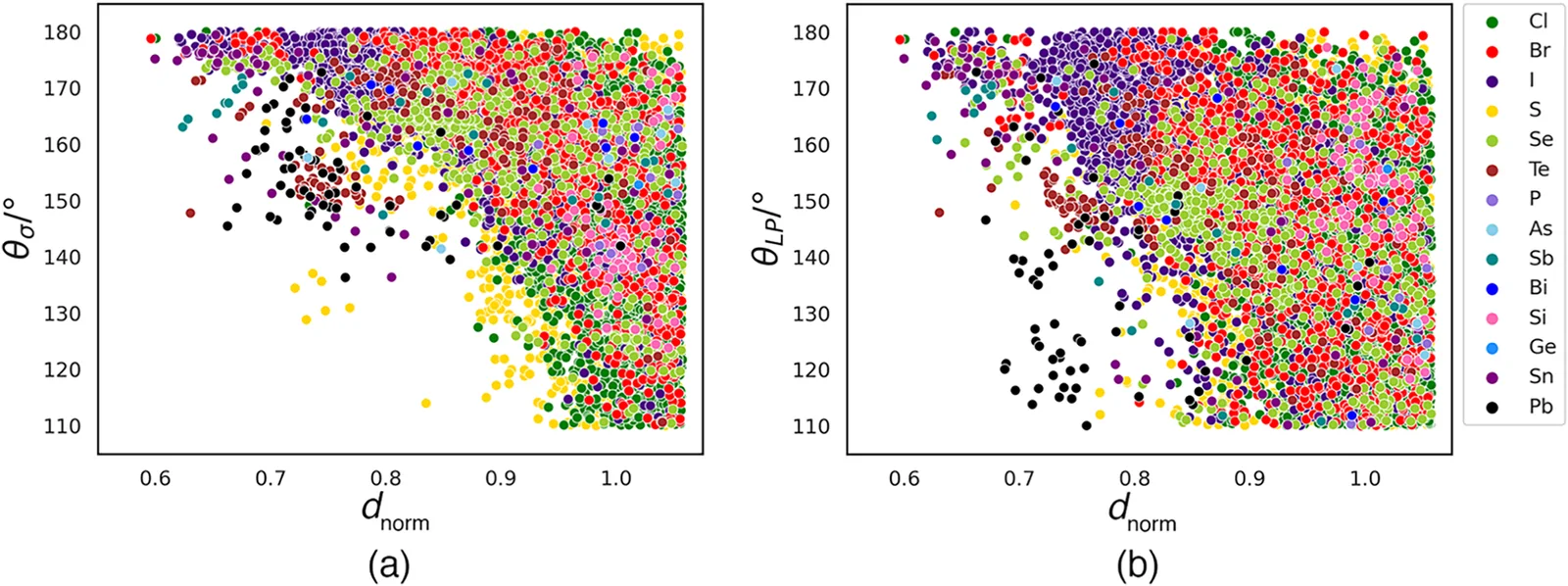
Distribution of geometries for σ-hole interactions involving N_sp^2^_ acceptor groups. (a) Normalized D_σ_···N distance (*d*_norm_) vs E–D_σ_···N σ-hole angle, θ_σ_. (b) Normalized D_σ_···N distance vs N_sp^2^_ lone-pair angle, θ_LP_. Data points are color-coded by σ-hole donor atom, D_σ_. Substituent E is as defined in Figure 2.

**Figure 8:**
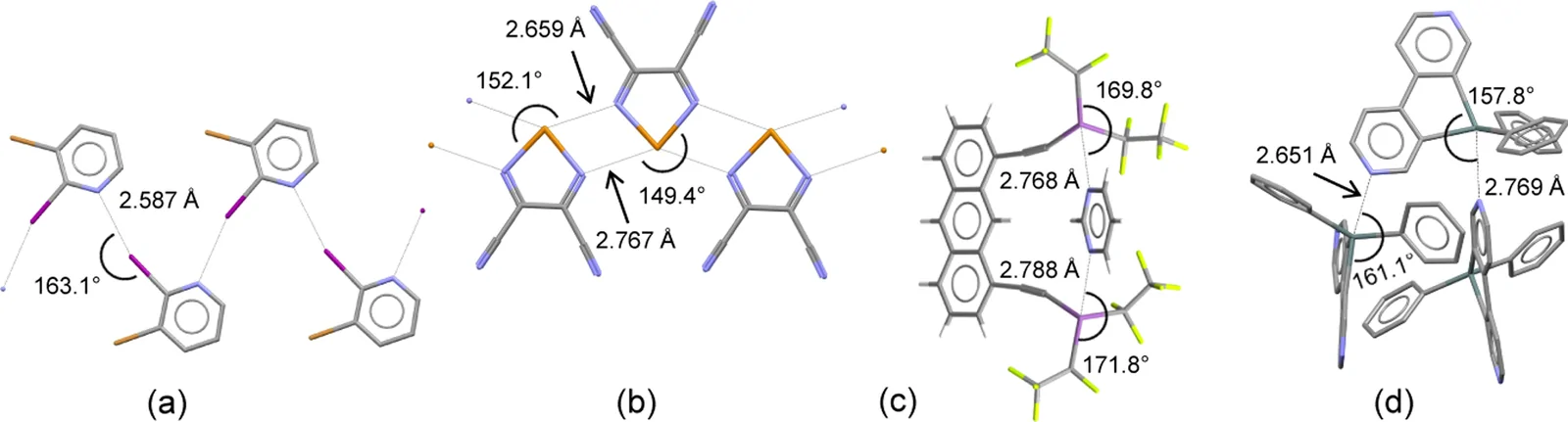
Crystal structures illustrating σ-hole interactions with short D_σ_···N_sp^2^_ distances: (a) C–I···N halogen bonding (*d*_norm_ = 0.70) in 3-bromo-2-iodo-pyridine (CSD refcode COBDUU);^59^ (b) N–Te···N chalcogen bonding (*d*_norm_ = 0.73, 0.76) in 1,2,5-telluradiazole-3,4-dicarbonitrile (CSD refcode AREGEK);^53^ (c) C–Sb···N pnictogen bonding (*d*_norm_ = 0.66, 0.67, 0.68, 0.68) in [anthracene-1,8-diylbis­(ethyne-2,1-diyl)]­bis­[bis­(pentafluoroethyl)­stibane] pyrimidine (CSD refcode NIXSIA);^60^ (d) C–Sn···N tetrel bonding (*d*_norm_ = 0.65, 0.68) in 9,9-diphenyl-9H-stannolo­[2,3-c:5,4-c′]­dipyridine chloroform solvate (CSD refcode OWUSAD).^61^ Hydrogen atoms not shown in (a) and (d); chloroform molecules not shown in (d).

Examination of the types of N_sp^2^_ Lewis bases involved in σ-hole interactions reveals the dominance of 5- and 6-membered-ring heterocycles (Table S6, Figures S15, S16). Thus, of 6002 halogen bonds, 5560 involve nitrogen as part of a heterocycle (1704 for 5-membered rings; 3487 for 6-membered rings). Similarly, of 5302 chalcogen bonds, 5119 involve nitrogen heterocycles (3782 for 5-membered rings; 1064 for 6-membered rings). The abundance of observations involving 6-membered rings for halogen bonds is consistent with the pervasive use of pyridyl or polypyridyl derivatives in halogen-bonding applications in supramolecular chemistry, a choice that is justified by the reliable preference for linearity of such interactions (θ_σ_ > 160°) particularly where X_σ_ = I, Br (Figure S15b). The abundance of observations involving 5-membered rings for chalcogen bonds (Figure S16a) is consistent with the reliable formation of (often strong) chalcogen bonds by 5-membered chalcazole-ring moieties (*vide supra*). The less pronounced preference for linearity with respect to the θ_LP_ angle relative to that observed for contacts involving N_sp^3^_ acceptor groups may be due to the increasing *s*-character and corresponding contraction of the nitrogen lone pair (from N_sp^3^_ to N_sp^2^_) or alternatively may be due to competition as σ-hole acceptor site from the π-system of the (aromatic) 5- or 6-membered ring present in the majority of N_sp^2^_ acceptor groups.

### σ-Hole Interactions with N_sp_ Acceptor Groups

sp-Hybridized nitrogen Lewis bases are inevitably dominated by nitrile/cyano groups (−C≡N), which are sterically more accessible for intermolecular interactions than their N_sp^3^_ and N_sp^2^_ counterparts. Distance vs angle plots for σ-hole interactions involving N_sp_ groups (*d*_norm_ vs θ_σ_ and *d*_norm_ vs θ_LP_) are presented in Figure 9. Illustrative examples of E–D_σ_···N_sp_ bonding are shown in Figure 10. Increasing linearity of the σ-hole angle for shorter *d*_norm_ (Figure 9a) is observed, analogous to the trend for N_sp^3^_ and N_sp^2^_ Lewis bases, albeit with fewer short interactions, consistent with the well-established trend in Lewis basicity (N_sp^3^_ > N_sp^2^_ > N_sp_) that arises from increasing nitrogen lone-pair contraction as a function of increasing *s*-character in this hybrid orbital. Breaking down these data by σ-hole donor class (D_σ_ = X_σ_, Ch_σ_, Pn_σ_, Tt_σ_; Figures S20a–S23a) and further by σ-hole donor atom type (Table 3) indicates that the shortest interactions can be found for pnictogen and tetrel bonds, for which interactions as short as *d*_norm_ ≈ 0.65 are observed, whereas interaction distances appear predominantly at *d*_norm_ > 0.75 for halogen bonds and *d*_norm_ > 0.8 for chalcogen bonds. The shortest tetrel bonds are associated with marked distortion from tetrahedral geometry about the tetrel atom (Tt_σ_) consistent with incipient hypervalent coordination^47^ or indeed incipient S_N_2 reaction.^62^ Halogen bonds show the most prominent directionality (*viz*. θ_σ_) for shorter interactions (Figure S20a), whereas chalcogen bonds, which are dominated by interactions for which Ch_σ_ = S, exhibit comparably less directionality for the θ_σ_ angle (Figure S18a). Trends in *d*_norm_ within classes of σ-hole donor resemble those noted for N_sp^3^_ and N_sp^2^_ Lewis bases (*vide supra*) wherein shorter mean distances are observed for the heavier, more polarizable donor atoms (D_σ_).

**Figure 9:**
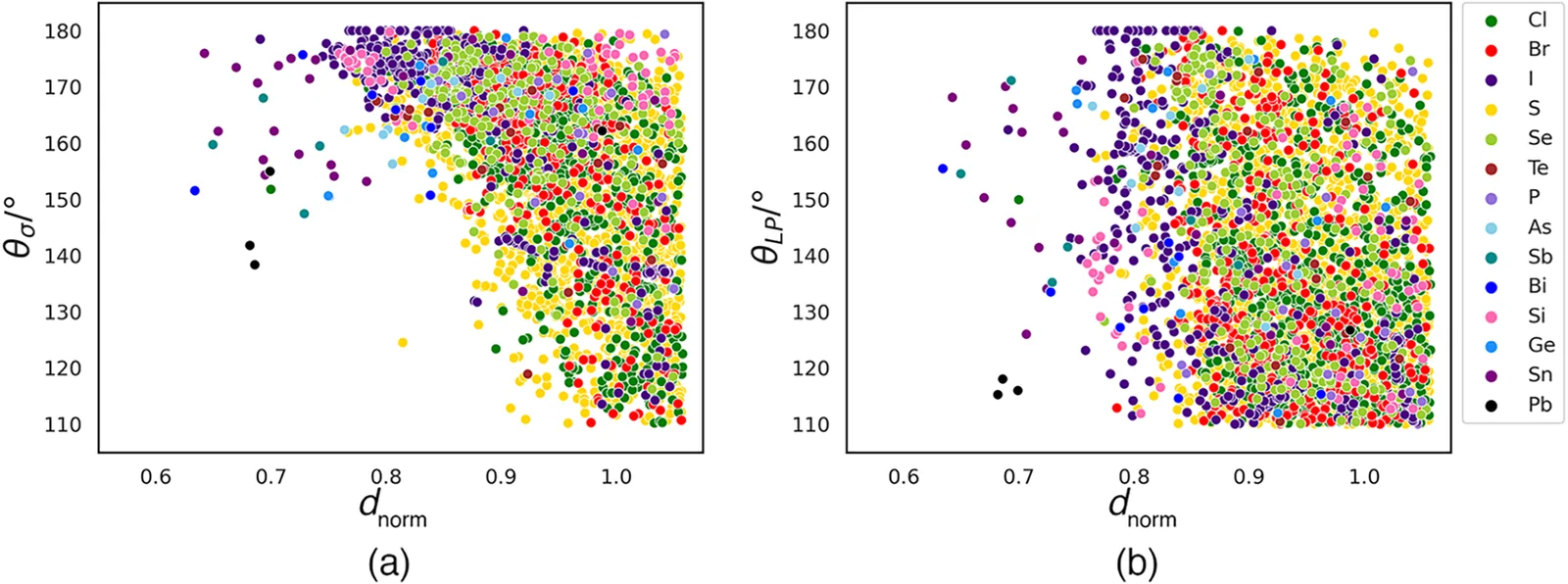
Distribution of geometries for σ-hole interactions involving N_sp_ acceptor groups. (a) Normalized D_σ_···N distance (*d*_norm_) vs E–D_σ_···N σ-hole angle, θ_σ_. (b) Normalized D_σ_···N distance vs N_sp_ lone-pair angle, θ_LP_. Data points are color-coded by σ-hole donor atom, D_σ_. Substituent E is as defined in Figure 2.

**Figure 10:**
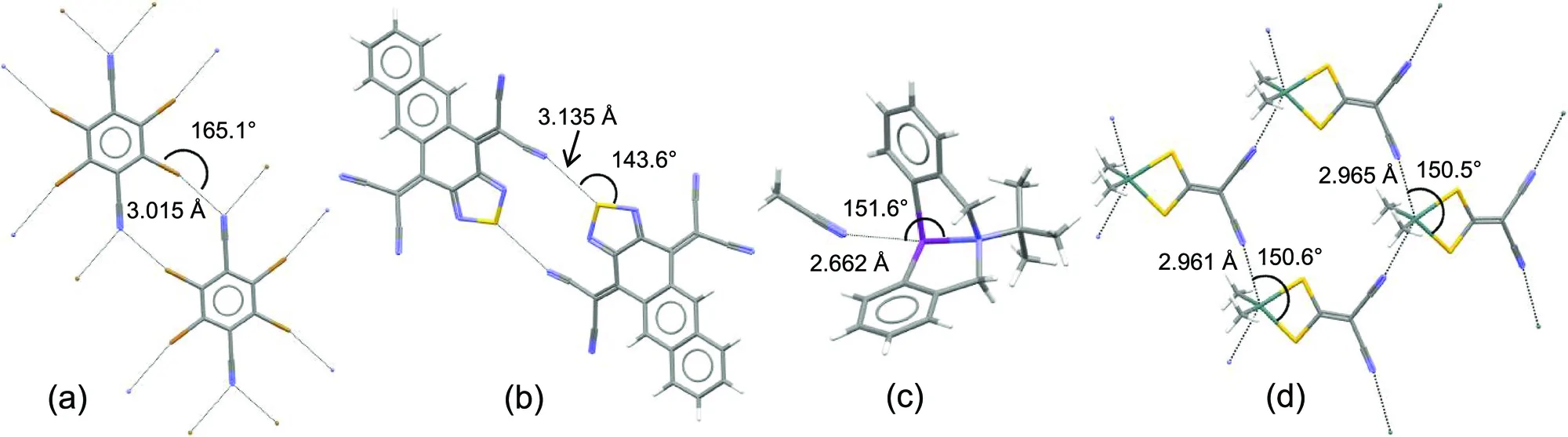
Crystal structures illustrating σ-hole interactions with short D_σ_···N_sp_ distances: (a) C–Br···N halogen bonding (*d*_norm_ = 0.86) in 2,3,5,6-tetrabromoterephthalonitrile (CSD refcode JOGPON);^69^ (b) N–S···N chalcogen bonding (*d*_norm_ = 0.88) in 2,2′-(anthra­[2,3-*c*]­[1,2,5]­thiadiazole-4,11-diylidene)­dipropanedinitrile (CSD refcode YACKUL01);^70^ (c) N–Bi···N pnictogen bonding (*d*_norm_ = 0.63) in 6-t-butyl-5,6,7,12-tetrahydrodibenzo­[*c*,*f*]­[1,5]­azabismocin-12-ylium tetrakis­(penta-fluorophenyl)­borate acetonitrile solvate (CSD refcode PICQIC);^71^ (d) S–Ge···N pnictogen bonding (*d*_norm_ = 0.75, 0.75) in 2-(2,2-Dimethyl-1,3,2-dithiagermetan-4-ylidene)­malononitrile (CSD refcode ASOWAG).^72^ The B­(C_6_F_5_)_4_^–^ anion is not shown in (c).

In contrast to observed trends for N_sp^3^_ and N_sp^2^_ Lewis bases, there is no obvious trend in directionality of interaction at the nitrogen atom (θ_LP_) as a function of distance, *d*_norm_ (Figures 9b and S20b–S23b). A likely reason for this observation is that the π-bond associated with all N_sp_ Lewis bases provides a reasonably competitive alternative to the lone pair in the σ-hole acceptor,^63^ effectively enabling a continuum of θ_LP_ angles that can accommodate crystal packing requirements driven by energetic/geometric optimization of other intermolecular interactions in the solid state. The observation and its contrast with the behavior for N_sp^3^_ and N_sp^2^_ Lewis bases is consistent with trends in interaction energy as a function of θ_LP_ angle as calculated by Symmetry Adapted Perturbation Theory (SAPT) analysis (*vide infra*). Specifically, as demonstrated in Figures S42c–S55c, there is little difference in σ-hole interaction energy as a function of θ_LP_ angle for E–D_σ_···N_sp_ interactions.

Shorter E–X_σ_···N_sp_ halogen bonds are found to contain a prominent population that involve metal cyanides (e.g., cyanometallates) as the N_sp_ Lewis base (Figure 11),^63–65^ consistent with the ability of cyanide ligands to accept hydrogen bonds^66^ and to form bridging interactions through N_sp_ Lewis basicity, e.g., in Prussian blue-type materials.^67^ Enhanced N_sp_ Lewis basicity that aids σ-hole acceptor capability can arise from π-back-donation from the metal center, combined with the overall negative charge associated with cyanometallates.^63^ Chalcogen bonding involving cyanometallates is also established^68^ (Figure S24), but the paucity of examples involving pnictogen or tetrel bonding suggests an opportunity for experimental exploration.

**Figure 11:**
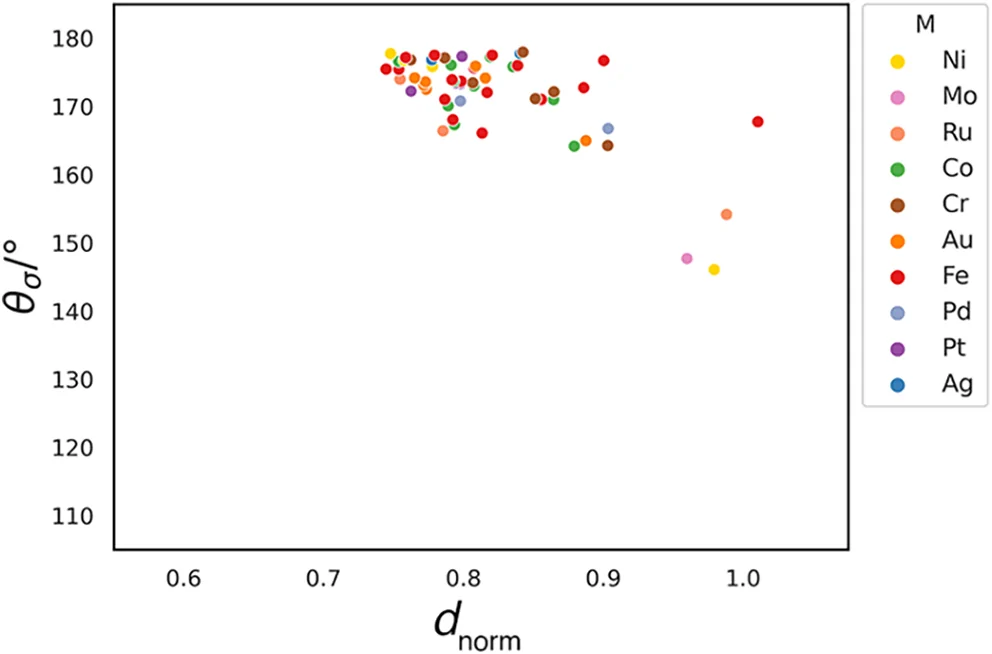
Distribution of normalized X_σ_···N distance (d_norm_) vs E–X_σ_···N σ-hole angle (θ_σ_) for halogen bonds to M–C≡N moieties (predominately involving cyanometallates). Data points color coded by metal (M) species.

### Substituent Effects on σ-Hole Donors (*E*–D_σ_)

Although the main focus of this report has been on the nitrogen σ-hole acceptor atoms, further insight regarding the effect of substituents on the σ-hole donor can be obtained. For halogen bonds, E–X_σ_···N, the predominant substituent is carbon (E = C), but the shortest and presumably strongest, halogen bonds are generated by dihalogens (i.e., X–X_σ_···N) or are N–X_σ_···N interactions (Figure 12) for N_sp^3^_ and N_sp^2^_ Lewis bases, where often X_σ_ = I, although these shortest interactions are largely absent (to date) for N_sp_ Lewis bases. N–X_σ_···N halogen bonds have also been studied extensively by ^15^N NMR spectroscopy in solution and can exhibit behavior very close to symmetric N···X···N interactions,^73,74^ although such symmetric interactions are outside the scope of the present study as they are classified as divalent halogen complexes within the CSD. Similar dominance, particularly of N–Ch_σ_···N interactions, can be found for chalcogen bonds with the shortest *d*_norm_ values, including those with N_sp_ chalcogen bond acceptors (Figure S25). Substituent effects are more difficult to discern for pnictogen bonds (Figure S26), mostly due to fewer observations, whereas tetrel bonds show an increasing presence of oxygen and sulfur (i.e., O–Tt_σ_···N, S–Tt_σ_···N) among the interactions with shorter *d*_norm_ values (Figure S27).

**Figure 12:**
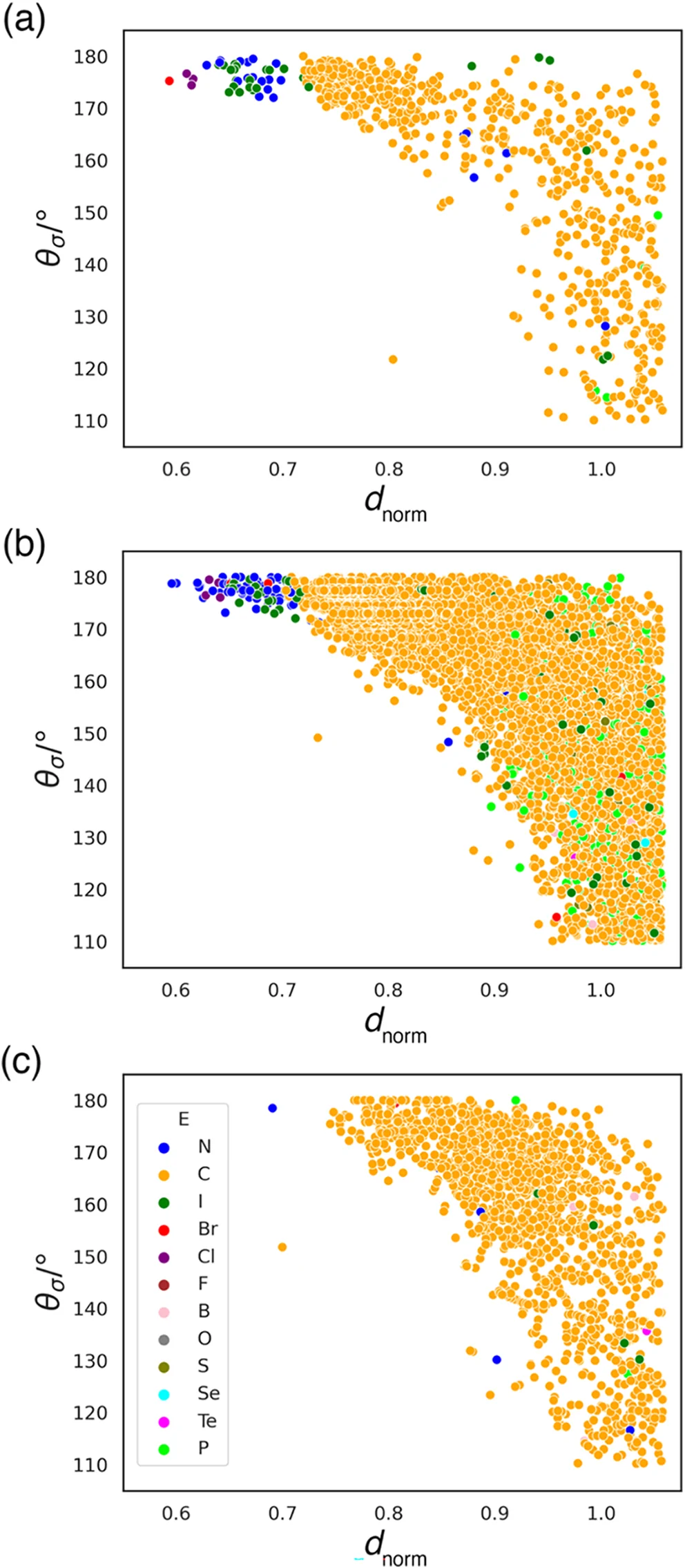
Distribution of geometries for halogen bonds involving (a) N_sp^3^_, (b) N_sp^3^_ and (c) N_sp_ acceptor groups represented as normalized X_σ_···N distance (*d*_norm_) vs E–X_σ_···N σ-hole angle, θ_σ_. Data points are color-coded by substituent (E) on the halogen-bond donor atom.

Carbon substituents (i.e., C–D_σ_···N interactions) are sufficiently prevalent that the effect of hybridization at carbon can be investigated. By analogy to C–H···N or C–H···O hydrogen bonds, one might anticipate an increased electron withdrawing effect (C_sp_ > C_sp^2^_ > C_sp^3^_) upon contraction of the carbon hybrid orbital involved in the C–D_σ_ bond (cf. C–H bond),^75^ to lead to shorter *d*_norm_ values for C–D_σ_···N interactions.^76^ For halogen bonds, minimum *d*_norm_ values are, in fact, similar for all hybridizations of carbon (Figure 13). This can be rationalized by the electron-withdrawing effect of C_sp_ substituents (alkynes) being matched by C_sp^2^_ and C_sp^3^_ bound to highly electron-withdrawing groups such as fluorine (e.g., as C_6_F_5_ or CF_3_ groups), which are often used in deliberate design of halogen-bonded assemblies.^13^ It is noticeable, however, that far fewer C_sp_–D_σ_···N interactions are reported than their C_sp^2^_ and C_sp^3^_ counterparts, which we attribute to less facile synthetic accessibility. Similar trends are observed for C–Ch_σ_···N chalcogen bonds, but numbers of observations are too few to make subdivisions based on carbon atom hybridization for pnictogen or tetrel bonds.

**Figure 13:**
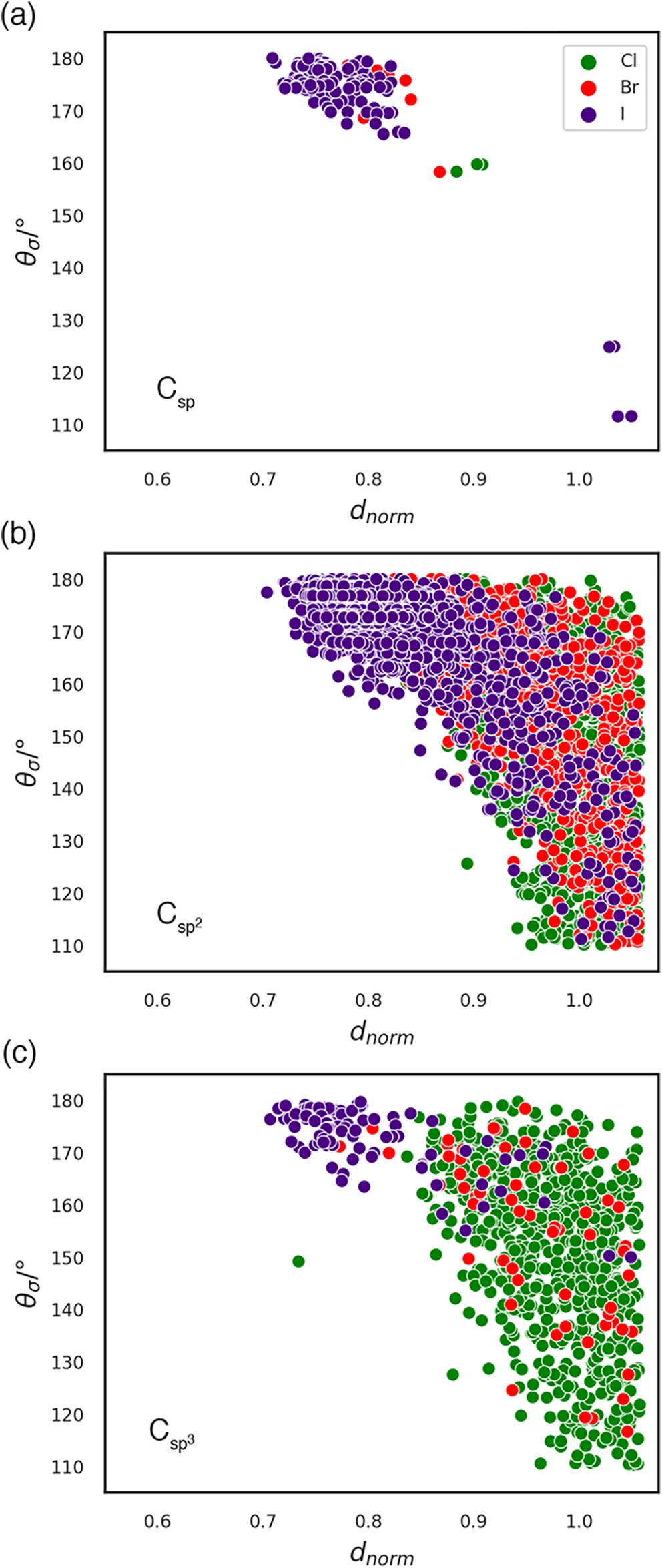
Distribution of geometries for halogen-bonding interactions involving N_sp^2^_ acceptor groups shown as normalized X_σ_···N distance (d_norm_) vs E– X_σ_···N σ-hole angle, θ_σ_ for (a) E = C_sp_, (b) E = C_sp^2^_, (c) E = C_sp^3^_. Data points are color-coded by halogen-bond donor atom, X_σ_.

## Energetic Trends

### Trends in Interaction Energies

To investigate the strength of σ-hole interactions involving nitrogen Lewis bases as acceptors, high-accuracy interaction energies were calculated for a range of “model” systems. The σ-hole donors selected for these systems were monocyano halogens (X), chalcogens (Ch), pnictogens (Pn), and tetrels (Tt), with the remaining valence saturated with methyl groups. The *X*CN, *Ch*(CN)­Me, *Pn*(CN)­Me_2_, and *Tt*(CN)­Me_3_ σ-hole donors were then combined with each of acetonitrile (N_sp_), imidazole (N_sp^2^_), and trimethylamine (N_sp^3^_) as typical examples of the three hybridizations that the Lewis basic nitrogen can adopt, resulting in a total of 42 σ-hole donor–acceptor-pair systems. Their geometries were freely optimized and counterpoise-corrected interaction energies were then calculated at these geometries, as described in the Section S1.2 of the Supporting Information. Although each σ-hole donor moiety possesses only one electron-withdrawing cyano group, satisfying the valence of the σ-hole donor atom requires a different number of methyl groups for each class of σ-hole interaction. As methyl groups are typically considered to be weakly electron-donating (although this position has recently been challenged by Elliot et al.^77^) the change in the number of attached methyl groups may affect the charge anisotropy that results in the σ-hole. Steric effects may also perturb these interactions unequally, especially for the tetrel-bond donors where three methyl groups surround the σ-hole. Despite these caveats, this choice of model systems should provide information on the trends in σ-hole interaction strengths, both within a given class of interaction, and across the different interaction types.

The interaction energies across the various σ-hole donor and acceptor moiety combinations are shown in Figure 14. For a given σ-hole type, the strength of the interactions increases with the polarizability of the σ-hole donor atom, which typically increases as a Group is descended. The trend is consistent with the experimentally observed general trends of increasing interaction prevalence (Figure 4), of shortening of mean *d*_norm_ values and of increasing tendency toward interaction linearity (θ_σ_ increasing toward 180°), with occasional exceptions where few observations are present (*N*_obs_ < 10; Tables 1–3). An outlier to the general trend of increasing polarizabilities on descending a Group is that Pb has a lower polarizability than Sn,^78^ but this is also observed in the interaction energies (C–Pb···*N* < C–Sn···N). There are currently too few experimental observations for tetrel bonds with Tt_σ_ = Pb, however, to enable comparison with geometric trends.

**Figure 14:**
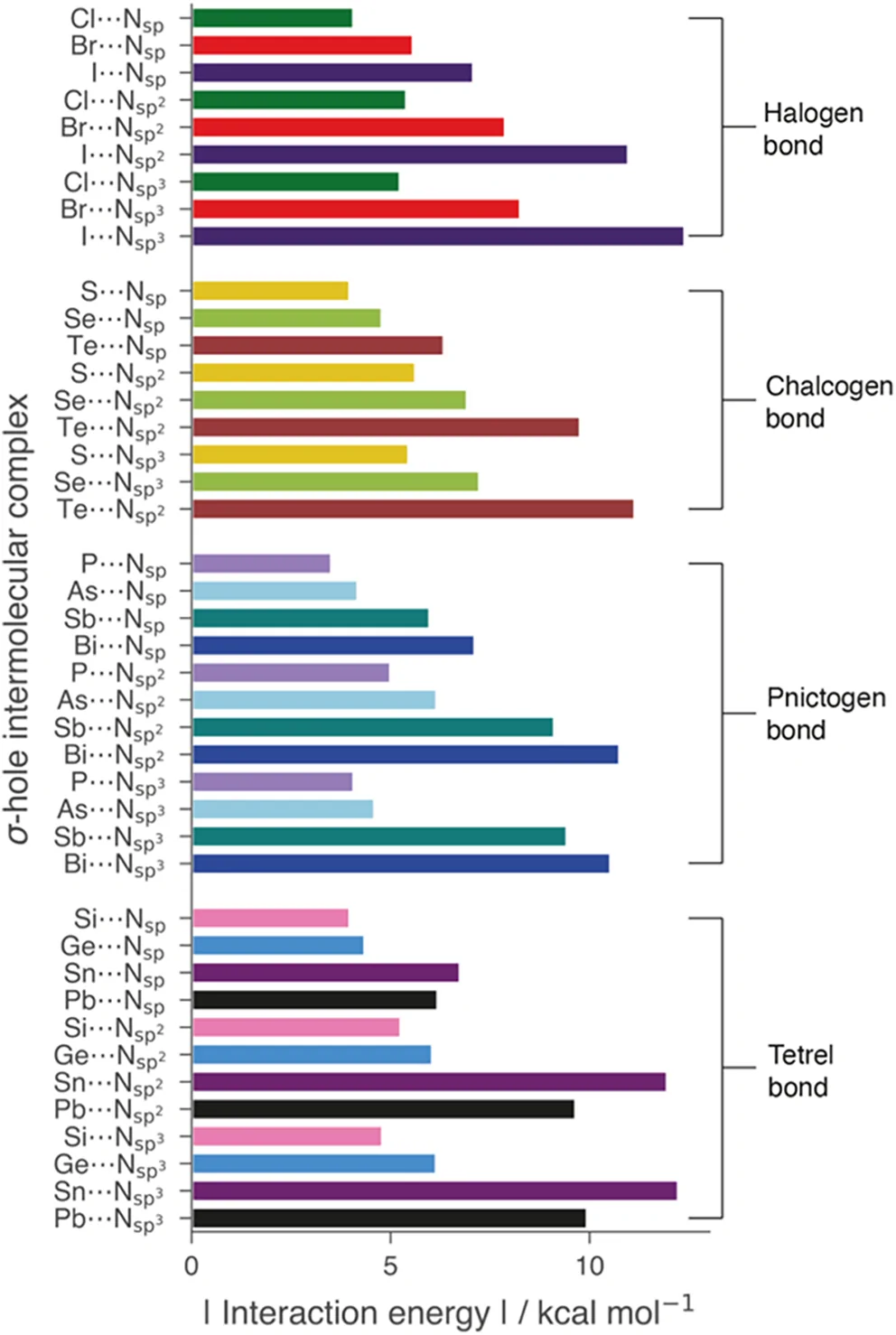
Counterpoise-corrected LCCSD­(T*)-F12b/cc-pVDZ­(-PP)-F12 absolute interaction energies for model σ-hole intermolecular complexes with acetonitrile (N_sp_), imidazole (N_sp^2^_), and trimethylamine (N_sp^3^_). The σ-hole donor molecules are abbreviated as the donor atom.

**Table 3:** Mean Geometries for σ-Hole Interactions Involving N_sp_ Acceptor Groups^a^

donor–acceptor (D_σ_···N)	mean *d*_norm_	mean D_σ_···N distance (Å)	mean θ_σ_ angle (°)	mean θ_LP_ angle (°)	number of observations (*N*_obs_)
Cl···N_sp_	0.950	3.305	155	136	776
Br···N_sp_	0.929	3.270	159	136	455
I···N_sp_	0.884	3.271	161	140	340
S···N_sp_	0.948	3.365	155	136	1899
Se···N_sp_	0.920	3.203	163	144	238
Te···N_sp_	0.912	3.327	156	137	18
P···N_sp_	0.958	3.409	162	140	17
As···N_sp_	0.845	2.993	166	154	10
Sb···N_sp_	0.759	3.134	161	147	6
Bi···N_sp_	0.803	3.260	164	132	8
Si···N_sp_	0.925	3.563	171	138	65
Ge···N_sp_	0.873	3.447	160	153	11
Sn···N_sp_	0.800	3.266	163	148	26

a Interactions with fewer than five observations for a given σ-hole donor are not shown (data provided in Table S8).

The strength of interaction between donor atoms from the same Period is roughly similar across the different classes of σ-hole interaction and, for the intermolecular complexes considered here, it is not conclusive whether, for example, a halogen bond is stronger than the analogous tetrel bond. Figure 14 shows that the strength of σ-hole interaction with respect to hybridization of the nitrogen atom typically increases with the amount of *p*-like character in the hybridization (sp^3^ > sp^2^ > sp). There are a few σ-hole donors for which this trend is reversed with respect to interactions of N_sp^2^_ and N_sp^3^_ acceptors (i.e., sp^2^ > sp^3^ > sp), notably for D_σ_ = Cl, S, P, Si in the third Period, D_σ_ = As in the fourth Period and D_σ_ = Bi in the sixth Period. The strength of these interactions is also very similar in magnitude for D_σ_ = Sb, Sn in the fifth Period. These observations are broadly consistent with trends in interaction distance, *d*_norm_, wherein steric factors are implicated in opposing the trend (sp^3^ > sp^2^ > sp) based upon nitrogen acceptor basicity (see General Discussion, *vide infra*).

### Trends in Directionality – the Guiding Influence of Sterics

To probe the underlying nature of the interactions that underpin the optimal σ-hole angle (θ_σ_) and lone-pair angle (θ_LP_) for the 42 model σ-hole interactions, stepped scans of each of these angles were performed individually, and at each step Symmetry-Adapted Perturbation Theory (SAPT) analysis of the intermolecular interaction energies was conducted. In each scan of θ_σ_ or θ_LP_, the angle was adjusted over the range 95–180° in 5° increments, with all other internal coordinates fixed, generating 18 sampling points. A SAPT2/def2-TZVPD calculation was carried out at each new geometry along the angle scans, using the Psi4 package, version 1.6.1 ^79^ (see Supporting Information for details of computational methods).

The SAPT energy components; dispersion, electrostatic, induction, and exchange, along with the total interaction energy, and their variation with σ-hole angle, plotted in Figure 15, show the C–D_σ_···N_sp^3^_ interactions (D_σ_ = I, Te, Sb, and Sn) of the previously described model σ-hole donor species. Only the interactions involving trimethylamine (NMe_3_) are presented, as the trends with σ-hole angle among the other nitrogen acceptors are analogous and are presented, along with plots for other σ-hole donor species, in Figures S29–S40. For all classes of σ-hole interaction, the strongest attractive interaction energy occurs when the σ-hole is aligned with the acceptor atom (θ_σ_ = 180°). The variation in the total interaction energy with respect to θ_σ_ also closely follows the trend in the exchange contribution, while all other SAPT components show weaker angular dependence. This is consistent with the findings of Stone and co-workers,^32,41^ later confirmed by Hill and Legon,^33^ with regards to halogen-bonded complexes. These previous findings challenged the widely held perception that the directionality in halogen bonding is primarily dictated by electrostatic attraction and concluded that although electrostatics are the main attractive contribution to the total binding energy, the directionality of the interactions is predominantly a consequence of exchange repulsion. The evidence from the current investigation suggests this is common across all major classes of σ-hole interaction.

**Figure 15:**
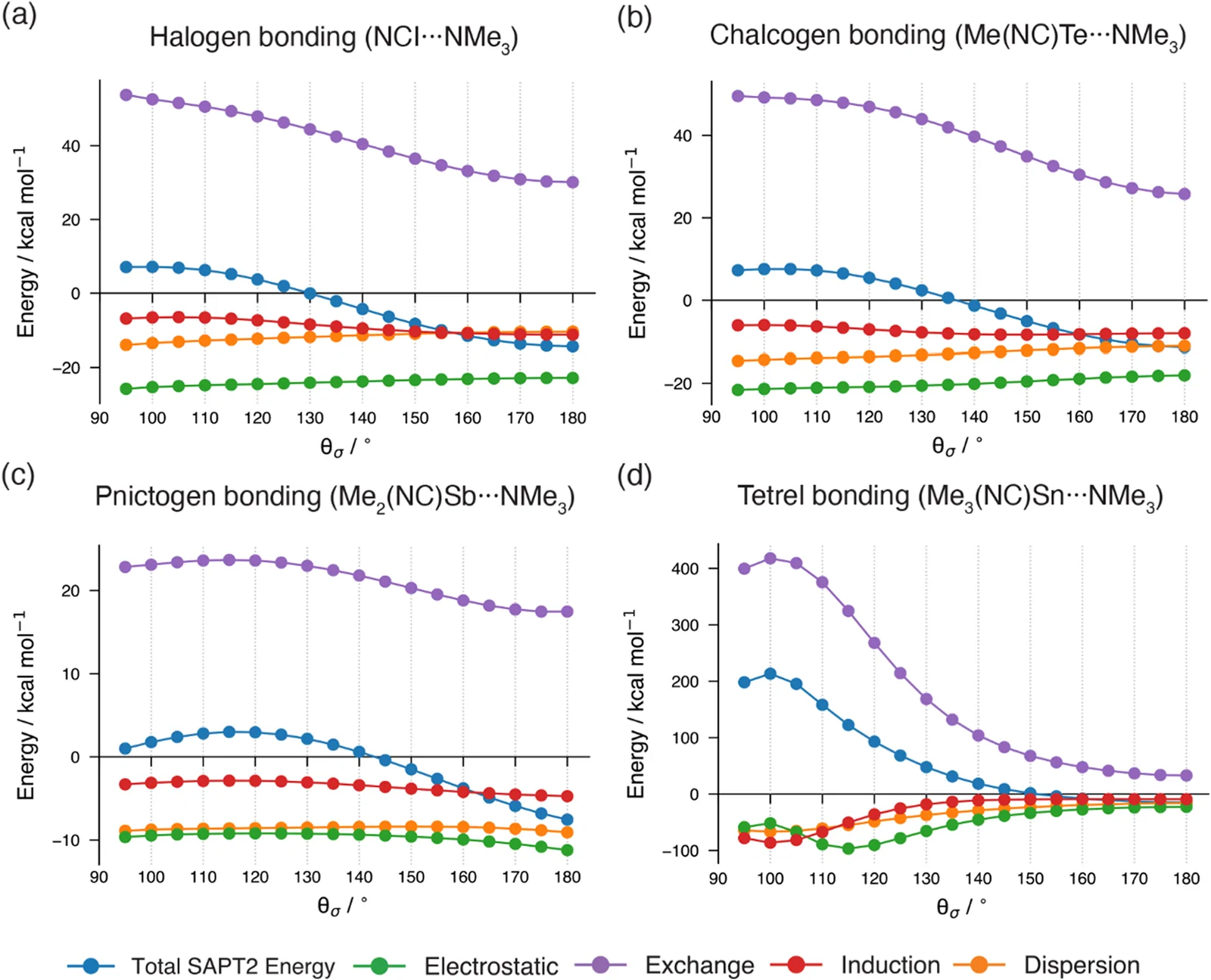
SAPT2/def2-TZVPD energetic components as a function of the σ-hole angle (θ_σ_) for: (a) halogen bonding between ICN and NMe_3_, (b) chalcogen bonding between Te­(CN)­Me and NMe_3_, (c) pnictogen bonding between Sb­(CN)­Me_2_ and NMe_3_, and (d) tetrel bonding between Sn­(CN)­Me_3_ and NMe_3_. The total SAPT2 interaction energy is abbreviated as “Total SAPT2 Energy”.

The halogen- and chalcogen-bonding θ_σ_ scans, shown in panels (a) and (b) of Figure 15, have similar trends and magnitudes in all energetic components. As expected from Figure 14, the chalcogen-bonding example (Te) has a slightly weaker total interaction energy than the halogen-bonding example (I) at θ_σ_ = 180° (SAPT2 interaction energies of −11.36 and −14.43 kcal mol^–1^, respectively), and becomes repulsive at θ_σ_ ≈ 135° or below, compared to 130° (or below) for the halogen-bonding motif. For the “tilting” of the pnictogen bonding interaction shown in Figure 15c, the total interaction energy becomes repulsive at θ_σ_ ≈ 145° and, in conjunction with the exchange component, reaches a maximum at θ_σ_ ≈ 120°, with the geometry corresponding to this point visualized in Figure S41a. In this geometric configuration, the lone pair on the N atom of the σ-hole acceptor is aligned with where VSEPR theory would position the lone pair on the Sb pnictogen-bond donor atom, rationalizing this repulsive maximum. The tetrel-bonding case (Figure 15d) shows strongly repulsive maxima for both the total interaction energy and exchange component at θ_σ_ ≈ 100°. Figure S41b reveals that in this geometry methyl groups on the σ-hole acceptor closely approach those on the σ-hole donor, leading to the strong repulsion. This is perhaps unsurprising as the tetrel-bond donors are the most sterically crowded of the σ-hole donors considered, with four groups covalently bound to the donor atom. When combined with the most sterically crowded acceptor molecule, trimethylamine (N_sp^3^_), this leads to clashes between the two interacting molecules as the σ-hole angle is varied, resulting in highly repulsive energies. The total interaction energy becomes repulsive at θ_σ_ ≈ 150° for the tetrel bonding case.

The variation in the interaction energy with θ_σ_ provides some information on when the interaction between the donor and acceptor becomes repulsive, and hence, potentially, what may be reasonable constraints on θ_σ_ for CSD searches such as those carried out in the present investigation. Figures S29–S40 demonstrate that this is dependent on both the σ-hole donor and the acceptor. In some systems, such as NCCl···NCMe (Figure S29a), the total interaction energy remains (weakly) attractive for all values of θ_σ_ probed and thus it would be difficult, from these data alone, to determine which angles may correspond to a σ-hole interaction. In other cases, the interaction becomes repulsive when θ_σ_ < ∼155°. More generally there is a tendency for the interaction to be attractive over a wider angular range for σ-hole donors, D_σ_, in the sequence X_σ_ > Ch_σ_ > Pn_σ_ > Tt_σ_. Clearly, in geometric searches of the CSD, any single angular constraint applied could exclude some attractive, electrostatically driven interactions, but may also capture interactions of lesser interest. The constraint of 110 ≤ θ_σ_ ≤ 180° used in this work is thus a compromise and intended to be inclusive of the vast potential scope of σ-hole interactions.

Varying the lone-pair angle, θ_LP_, can be thought of as varying the direction of the Lewis base moiety relative to the σ-hole donor (i.e., rotation of the σ-hole acceptor about the nitrogen atom). Figure 16 plots the changes in SAPT energy components as a function of the variation in θ_LP_ for the interaction of the ICN halogen bond donor with acetonitrile (NCMe), imidazole [N­(NH)­(CH)_3_], and trimethylamine (NMe_3_). Similar plots for the other classes of σ-hole interaction are mostly analogous and can be found in Figures S42–S55. For the σ-hole interactions involving acetonitrile and imidazole as acceptors, it can be seen that all of the SAPT components, including exchange, are much less sensitive to variation in θ_LP_ than θ_σ_, with the components in Figure 16b,c, for example, only showing a notable change as θ_LP_ nears 90°. From the geometric data (*vide supra*), this insensitivity to θ_LP_ is most evident for N_sp_ acceptors (Figure 9b), but lone-pair directionality is only evident at the shortest *d*_norm_ values even for N_sp^2^_ acceptors (Figure 7b). A likely rationale for the energetic trends with θ_LP_ angle for both acetonitrile (N_sp_) and imidazole (N_sp^2^_) is the proximity of a π-system that can act as an orthogonally oriented Lewis base in competition to the lone pair of the σ-hole acceptor. As shown in Figure 16a, the variation in SAPT components with θ_LP_ is much more dramatic for σ-hole interactions to trimethylamine, which is the acceptor with the greatest steric bulk. The large total (repulsive) interaction energy and exchange term for the lower values of θ_LP_ immediately suggests a steric interaction, even for the halogen bonding examples. As a consequence of the large repulsive interaction, an inset with expanded ordinate (energy) scale is necessary to depict the magnitudes of the attractive SAPT components for θ_LP_ > 130°. The inset shows that this particular halogen-bonding interaction (NC–I···NMe_3_) is slightly more attractive than the corresponding interactions with N_sp^2^_ and N_sp_ species, but is repulsive for θ_LP_ < ∼130°. This is consistent with the geometric trends observed for θ_LP_ for N_sp^3^_ acceptors (Figure 5b) relative to their N_sp^2^_ and N_sp_ counterparts. The angular region (Δθ_LP_) where the analogous interaction is attractive for N_sp^3^_ acceptors becomes even narrower for pnictogen and tetrel bonding (Figures S52 and S53).

**Figure 16:**
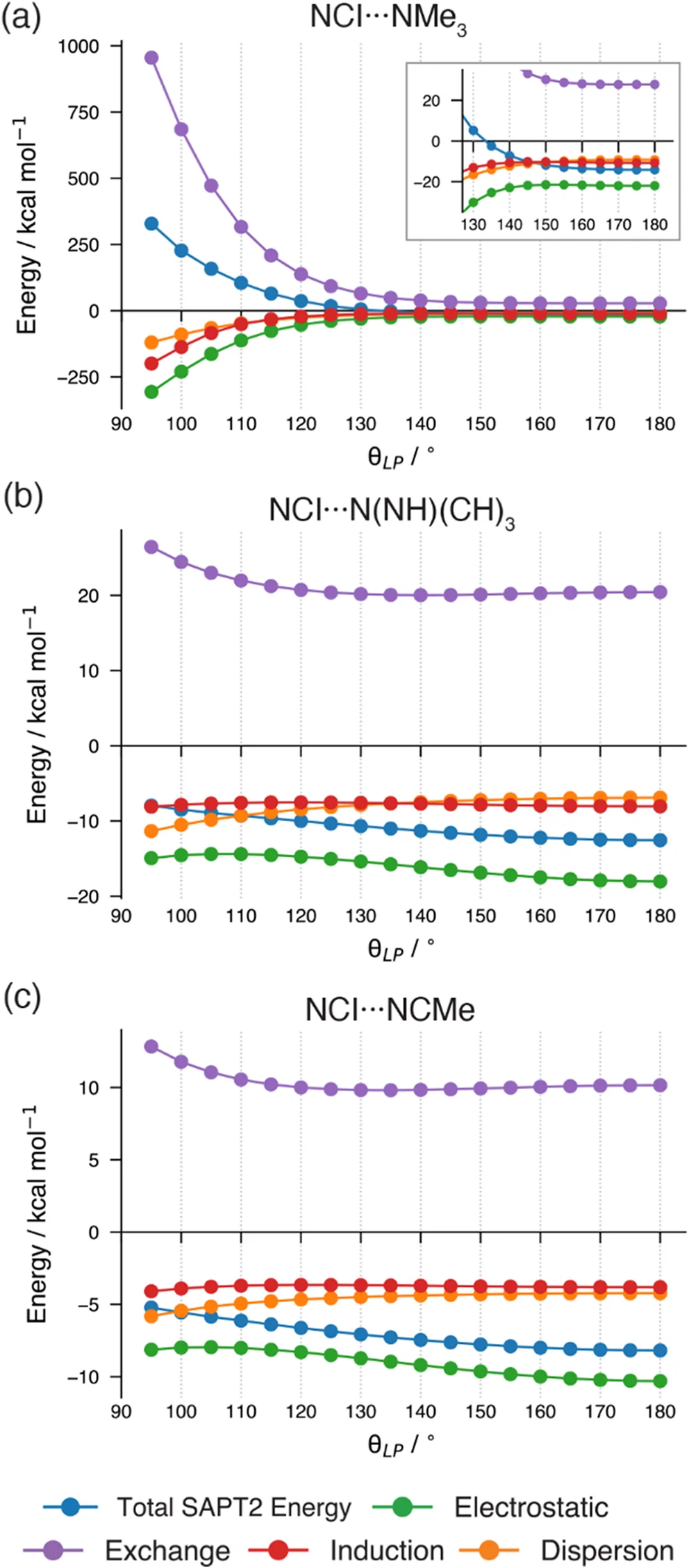
SAPT2/def2-TZVPD energy components as a function of the lone-pair angle (θ_LP_) for (a) halogen bonding between ICN and NMe_3_, (b) halogen bonding between ICN and imidazole, and (c) halogen bonding between ICN and MeCN. The total SAPT2 interaction energy is abbreviated as “Total SAPT2 Energy”. The inset to panel (a) shows the change in the energy components over the range θ_LP_ = 130–180°.

## Overview and Perspective

A number of overarching trends can be discerned from the geometric and energetic data presented herein. Three geometric measurements have been considered for each σ-hole interaction. The donor–acceptor distance (D_σ_···N) has been normalized (*d*_norm_) relative to the sum of van der Waals radii of the interacting atoms to enable more meaningful establishment of trends across all interactions. Although statistical normalisation of angle distributions for intermolecular interactions is also possible,^38,41,80^ this is sometimes perceived as a less straightforward interpretation. We have therefore presented data for the two key angles that define σ-hole interaction (θ_σ_ and θ_LP_) without further normalization. Mean geometric data across different interaction classes are summarized in Figure 17. The high-accuracy *ab initio* calculations of interaction energies are conducted on gas-phase molecular pairs, but trends in these energies are consistent with geometric trends arising from the experimental data in the solid state (Figure S28).

**Figure 17:**
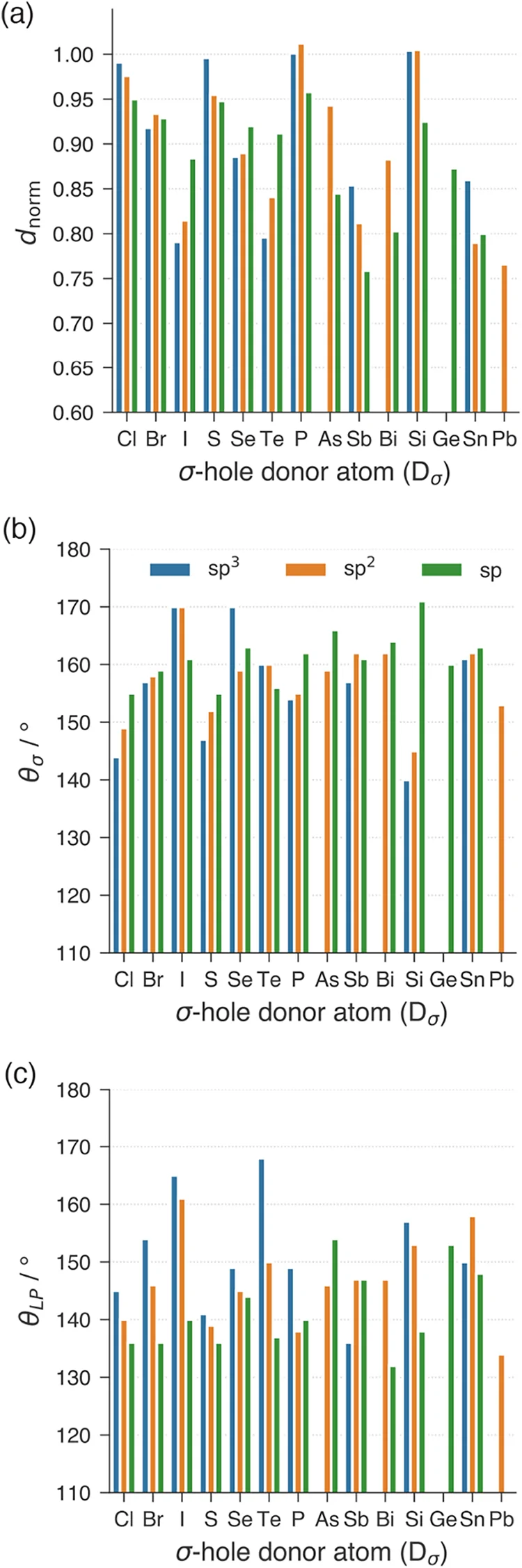
Trend in experimental (a) mean *d*_norm_ values, (b) mean θ_σ_ and (c) mean θ_LP_ as a function nitrogen hybridization of acceptor groups (N_sp^3^_, blue; N_sp^2^_, orange; N_sp_, green) shown for each σ-hole donor atom (D_σ_).

There is a general trend toward linearity in both θ_σ_ and θ_LP_ angles upon shortening of the interaction distance (*d*_norm_) across all classes of σ-hole interaction studied, although the comprehensive nature of the study has enabled some key exceptions to be identified. Thus, short interaction distances with nonlinear geometries (*d*_norm_ < 0.8; θ_σ_ < 160°) are prominent for cyclic chalcazoles which form molecular dimers by self-association via a pair of inversion-related N–Ch···N chalcogen bonds. Lone-pair directionality (θ_LP_) of the interactions is not evident for N_sp_ acceptor groups and is diminished for N_sp^2^_ acceptor groups relative to their N_sp^3^_ counterparts, conclusions that are rationalized by the energy decomposition analyses as a function of θ_LP_ angle (e.g., see Figure S56).

A prominent conclusion from both analysis of geometric trends and energy decomposition calculations is that although electrostatic attraction (the foundation of the σ-hole concept) is an important attractive driving force in σ-hole interactions, steric factors play a major role in defining interaction geometry, most prominently in the angles (θ_σ_ and θ_LP_), but also in the donor–acceptor separation (*d*_norm_). Steric encumbrance increases with the number of substituents associated with the donor atom (Tt_σ_ > Pn_σ_ > Ch_σ_ > X_σ_) or the acceptor atom (N_sp^3^_ > N_sp^2^_ > N_sp_). The general trend in *d*_norm_ values as a function of nitrogen hybridization of acceptor groups is N_sp^3^_ < N_sp^2^_ < N_sp_ for halogen bonds and chalcogen bonds of each donor atom (except for the weakest/longest interactions where D_σ_ = Cl or S), consistent with interaction distance being driven by the attractive component(s) of the interaction. By contrast, for pnictogen bonds and tetrel bonds *d*_norm_ values decrease in the sequence N_sp^3^_ > N_sp^2^_ > N_sp_, consistent with a dominant role of steric interactions. These trends are reflected to some extent in the (gas-phase) interaction energy calculations (Figures 14 and S28), which show a general trend of stronger interactions for halogen bonds and chalcogen bonds in the (acceptor) sequence N_sp_ < N_sp^2^_ < N_sp^3^_. For pnictogen and tetrel bonds, however, the trend in energies is N_sp_ < N_sp^2^_ ≈ N_sp^3^_ and for the lighter σ-hole donor, for which there are shorter interaction distances (*d* rather than *d*_norm_) that may accentuate steric effects, E–D_σ_···N_sp^2^_ are stronger interactions than E–D_σ_···N_sp^3^_. The importance of steric influence on interaction geometry is further emphasized by the prominent contribution of the exchange repulsion to the interaction energy as interaction geometries (θ_σ_ and θ_LP_) deviate substantially from linear (Sections S6 and S7), in particular for the most sterically encumbered σ-hole donors and acceptors. To further support the importance of (overcoming) steric interactions, the trend across all four general families of σ-hole interactions is one of decreasing prevalence (i.e., likelihood of formation, *vide supra*) with increasing number of substituents at the acceptor atom, a trend that is more prominent for the more sterically encumbered σ-hole donors (Section S2).

Beyond these delineated trends other features emerge and opportunities are presented. Thus, the prominent role of cyclic imines as (N_sp^2^_) σ-hole acceptors is clearly evident and presumably arises in part due to efficacy and in part due to synthetic accessibility. This leads directly to overall numbers of E–D_σ_···N_sp^2^_ interactions being greatest overall (70.7% of all σ-hole interactions) and for each of the four families of interactions. The potential of cyanometallates to form short (strong) E–D_σ_···N_sp_ σ-hole interactions is identified for halogen and chalcogen bonds, but is unexplored for Tt_σ_ and Pn_σ_ donors, interactions which should not be prohibited by steric restrictions, thereby presenting an opportunity for future investigation. Throughout this presentation we have also been able to use overarching trends to identify specific deviations from these trends, often in classes or small groups of compounds. Where possible we have directly identified or at least suggested the underlying chemical rationale for such behaviors, but these observations also present opportunities for more detailed future experimental and computational investigation.

Finally it is important to recognize, as we noted at the outset, that these comprehensive studies have wider implications for nitrogen Lewis bases in, for example, synthetic organic chemistry, coordination chemistry and through hydrogen bonding in supramolecular chemistry and structural biology. Thus, many of the observations, trends and explanations provided herein will have parallels in these other areas of chemistry.

## Supplementary Material



## Data Availability

A list of REFCODEs for all structures identified in each search of the CSD and Cartesian coordinates for the optimized geometries of the interacting molecular pairs used to calculated interaction energies in Figure 14 are available via the University of Sheffield ORDA research data repository at 10.15131/shef.data.32270448.

## Notes

Description of Methods; comprehensive presentation of experimental geometric data in tabular and graphical form; and graphical presentation of interaction energy decomposition analyses of σ-hole interactions for all donor–acceptor pairs (PDF)

## References

[1] Rochlin P., Murphy D. B., Help S. (1954). Relative Basicities of Some Organic
Nitrogen Bases. J. Am. Chem. Soc..

[2] Catalan J., Abboud J. L. M., Elguero J. (1987). Basicity and Acidity
of Azoles. Adv. Heterocycl. Chem..

[3] Headley A. D. (1991). Quantitative
Analysis of Solvation Effects and the Influence of Alkyl Substituents
on the Basicity of Amines. J. Org. Chem..

[4] Denmark S. E., Beutner G. L. (2008). Lewis Base Catalysis
in Organic Synthesis. Angew. Chem., Int. Ed..

[5] Yang Y., Zhang D., Vessally E. (2020). Direct Amination
of Aromatic C–H
Bonds with Free Amines. Top. Curr. Chem..

[6] Pombeiro A. J. L. (2019). Nitrogen
Ligands. Dalton Trans..

[7] Togni A., Venanzi L. M. (1994). Nitrogen Donors
in Organometallic Chemistry and Homogeneous
Catalysis. Angew. Chem., Int. Ed. Engl..

[8] Fache F., Dunjic B., Gamez P., Lemaire M. (1997). Recent advances in
homogeneous and heterogeneous asymmetric catalysis with nitrogen-containing
ligands. Topics Catal..

[9] Desiraju, G. R. ; Steiner, T. The Weak Hydrogen Bond, IUCr Monographs on Crystallography 9; Oxford University Press: Oxford, 1999.

[10] Jeffrey, G. A. ; Saenger, W. Hydrogen Bonding in Biological Structures; Springer-Verlag: Berlin, 1991.

[11] Brammer L. (2017). Halogen bonding,
chalcogen bonding, pnictogen bonding, tetrel bonding: origins, current
status and discussion. Faraday Discuss..

[12] Brammer L., Peuronen A., Roseveare T. M. (2023). Halogen bonds, chalcogen bonds, pnictogen
bonds, tetrel bonds and other σ-hole interactions: a snapshot
of current progress. Acta Crystallogr., Sect.
C.

[13] Cavallo G., Metrangolo P., Milani R., Pilati T., Priimagi A., Resnati G., Terraneo G. (2016). The Halogen Bond. Chem. Rev..

[14] Vogel L., Wonner P., Huber S. M. (2019). Chalcogen
Bonding: An Overview. Angew. Chem., Int. Ed..

[15] Scheiner S. (2013). The Pnicogen
Bond: Its Relation to Hydrogen, Halogen, and Other Noncovalent Bonds. Acc. Chem. Res..

[16] Bauzá A., Mooibroek T. J., Frontera A. (2016). Tetrel Bonding Interactions. Chem. Rec..

[17] Alcock N. W. (1972). Secondary
Bonding to Nonmetallic Elements. Adv. Inorg.
Chem. Radiochem..

[18] Alkorta I., Elguero J., Frontera A. (2020). Not Only Hydrogen Bonds:
Other Noncovalent
Interactions. Crystals.

[19] Pancholi J., Beer P. D. (2020). Halogen bonding
motifs for anion recognition. Coord. Chem. Rev..

[20] Yamada S., Konno T. (2020). Recent Advances in
Halogen Bond-assisted Organic Synthesis. Curr.
Org. Chem..

[21] Zheng J., Suwardi A., Wong C. J. F., Loh X. J., Li Z. (2021). Halogen bonding
regulated functional nanomaterials. Nanoscale
Adv..

[22] Costa P. J., Nunes R., Vila-Viçosa D. (2019). Halogen bonding
in halocarbon–protein
complexes and computational tools for rational drug design. Expert Opin. Drug Discovery.

[23] Yan W., Zheng M., Xu C., Chen F. E. (2021). Harnessing noncovalent
interaction of chalcogen bond in organocatalysis: From the catalyst
point of view. Green Synth. Catal..

[24] Ho P. C., Wang J. Z., Meloni F., Vargas-Baca I. (2020). Chalcogen
bonding in materials chemistry. Coord. Chem.
Rev..

[25] Fourmigué M., Dhaka A. (2020). Chalcogen bonding in
crystalline diselenides and selenocyanates:
from molecules of pharmaceutical interest to conducting materials. Coord. Chem. Rev..

[26] Mulliken R. S. (1952). Molecular
Compounds and their Spectra. J. Am. Chem. Soc..

[27] Huber S. M., Scanlon J. D., Jimenez-Izal E., Ugalde J. M., Infante I. (2013). On the directionality
of halogen bonding. Phys. Chem. Chem. Phys..

[28] Bent H. A. (1968). Structural
chemistry of donor-acceptor interactions. Chem.
Rev..

[29] Clark T., Hennemann M., Murray J. S., Politzer P. (2007). Halogen bonding: the
σ-hole. J. Mol. Model..

[30] Politzer P., Murray J. S., Clark T., Resnati G. (2017). The σ-hole revisited. Phys.
Chem. Chem. Phys..

[31] Politzer P., Murray J. S. (2017). σ-Hole Interactions: Perspectives and Misconceptions. Crystals.

[32] Stone A. J. (2013). Are Halogen
Bonded Structures Electrostatically Driven?. J. Am. Chem. Soc..

[33] Hill J. G., Legon A. C. (2015). On the directionality and non-linearity of halogen
and hydrogen bonds. Phys. Chem. Chem. Phys..

[34] de
Azevedo Santos L., Ramalho T. C., Hamlin T. A., Bickelhaupt F. M. (2023). Intermolecular
Covalent Interactions: Nature and Directionality. Chem. - Eur. J..

[35] Politzer P., Murray J. S., Clark T. (2013). Halogen bonding and other σ-hole
interactions: a perspective. Phys. Chem. Chem.
Phys..

[36] Lim J. Y.
C., Beer P. D. (2018). Sigma-Hole
Interactions in Anion Recognition. Chem.

[37] Groom C. R., Bruno I. J., Lightfoot M. P., Ward S. C. (2016). The Cambridge Structural
Database. Acta Crystallogr., Sect. B.

[38] Brammer L., Bruton E. A., Sherwood P. (2001). Understanding
the Behavior of Halogens
as Hydrogen Bond Acceptors. Cryst. Growth Des..

[39] Bauzá A., Quiñonero D., Deyà P. M., Frontera A. (2013). Halogen bonding versus
chalcogen and pnicogen bonding: a combined Cambridge structural database
and theoretical study. CrystEngComm.

[40] Metrangolo P., Murray J. S., Pilati T., Politzer P., Resnati G., Terraneo G. (2011). The Fluorine Atom as
a Halogen Bond Donor, viz. a Positive
Site. CrystEngComm.

[41] Lommerse J. P. M., Stone A. J., Taylor R., Allen F. H. (1996). The Nature and Geometry
of Intermolecular Interactions between Halogens and Oxygen or Nitrogen. J. Am. Chem. Soc..

[42] From search of CSD (Nov. 2024 update): crystal structures containing (i) monovalent Cl/Br/I = 330,071, (ii) divalent S/Se/Te = 126,011, trivalent P/As/Sb/Bi = 20,126, tetravalent Si/Ge/Sn/Pb = 72,318).

[43] Allen F. H., Motherwell W. D. S., Raithby P. R., Shields G. P., Taylor R. (1999). Systematic
analysis of the probabilities of formation of bimolecular hydrogen-bonded
ring motifs in organic crystal structures. New
J. Chem..

[44] Galek P. T. A., Fábián L., Motherwell W. D. S., Allen F. H., Feeder N. (2007). Knowledge-based model
of hydrogen-bonding
propensity in organic crystals. Acta Crystallogr.,
Sect. B.

[45] Macrae C. F., Bruno I. J., Chisholm J. A., Edgington P. R., McCabe P., Pidcock E., Rodriguez-Monge L., Taylor R., van de Streek J., Wood P. A. (2008). *Mercury
CSD 2.0* – New Features for the Visualization and Investigation
of Crystal Structures. J. Appl. Crystallogr..

[46] Clark T. (2013). σ-Holes. WIREs Comput. Mol. Sci..

[47] Crabtree R. H. (2017). Hypervalency,
secondary bonding and hydrogen bonding: siblings under the skin. Chem. Soc. Rev..

[48] Arhangelskis M., Topić F., Hindle P., Tran R., Morris A. J., Cinčić D., Friščić T. (2020). Mechanochemical
reactions of cocrystals: comparing theory with experiment in the making
and breaking of halogen bonds in the solid state. Chem. Commun..

[49] Fellowes T., White J. M. (2019). New insights into chalcogen bonding provided by co-crystal
structures of benzisoselenazolinone derivatives and nitrogen bases. CrystEngComm.

[50] García-Moreno E., Cerrada E., Bolsa M. J., Luquin A., Laguna M. (2013). Water-Soluble
Phosphanes Derived from 1,3,5-Triaza-7-phosphaadamantane and Their
Reactivity towards Gold­(I) Complexes. Eur. J.
Inorg. Chem..

[51] Wang L.-S., Zhang J.-J., Du W.-X., Hu S.-M., Fu R.-B., Xia S.-Q., Wu X.-T., Xiang S.-C., Li Y.-M., Fu Z.-Y. (2005). Syntheses and Characterizations of (4-NH_2_C_6_H_4_S)_4_Sn and [(4-NH_2_C_6_H_4_S)_2_(4-NH_3_C_6_H_4_S)_2_Sn]­Cl_2_. Chin. J. Struct.
Chem..

[52] Nag T., Ovens J. S., Bryce D. L. (2023). ^77^Se and ^125^Te solid-state NMR and X-ray diffraction structural
study of chalcogen-bonded
3,4-dicyano-1,2,5-chalcogenodiazole cocrystals. Acta Crystallogr., Sect. C.

[53] Cozzolino A. F., Yang Q., Vargas-Baca I. (2010). Engineering Second-Order Nonlinear
Optical Activity by Means of a Noncentrosymmetric Distortion of the
[Te-N]_2_ Supramolecular Synthon. Cryst.
Growth Des..

[54] Chulanova E. A., Radiush E. A., Balmohammadi Y., Beckmann J., Grabowsky S., Zibarev A. V. (2023). New charge-transfer complexes of 1,2,5-chalcogenadiazoles
with tetrathiafulvalenes. CrystEngComm.

[55] Pushkarevsky N. A., Lonchakov A. V., Semenov N. A., Lork E., Buravov L. I., Konstantinova L. S., Silber G. T., Robertson N., Gritsan N. P., Rakitin O. A., Woollins J. D., Yagubskii E. B., Beckmann J., Zibarev A. V. (2012). First charge-transfer
complexes between
tetrathiafulvalene and 1,2,5-chalcogenadiazole derivatives: Design,
synthesis, crystal structures, electronic and electrical properties. Synth. Met..

[56] Thomas S. P., Satheeshkumar K., Mugesh G., Guru Row T. (2015). Unusually Short Chalcogen
Bonds Involving Organoselenium: Insights into the Se–N Bond
Cleavage Mechanism of the Antioxidant Ebselen and Analogues. Chem. - Eur. J..

[57] Gao Y., Chen Y., Li R., Bian Y., Li X., Jiang J. (2009). Nonperipherally Octa­(butyloxy)-Substituted
Phthalocyanine Derivatives
with Good Crystallinity: Effects of Metal-Ligand Coordination on the
Molecular Structure, Internal Structure, and Dimensions of Self-Assembled
Nanostructures. Chem. - Eur. J..

[58] Yuan Y.-Z., Zhou J., Liu X., Liu L.-H., Yu K.-B. (2007). A new hemidirected
lead­(II) complex converted into holodirected by secondary Pb-N interactions. Inorg. Chem. Commun..

[59] Dhau J. S., Singh A., Kasetti Y., Bhatia S., Bharatam P. V., Brandao P., Felix V., Singh K. N. (2013). A study
on the BF_3_ directed lithiation of 3-chloro- and 3-bromopyridine. Tetrahedron.

[60] Beckmann J. L., Krieft J., Vishnevskiy Y. V., Neumann B., Stammler H.-G., Mitzel N. W. (2023). A Bidentate Antimony
Pnictogen Bonding Host System. Angew. Chem.,
Int. Ed..

[61] Ohshita J., Yamaji K., Adachi Y. (2020). Crystal Structures
and Phosphorescent
Properties of Group 14 Dipyridinometalloles and Their Copper Complexes. ChemPlusChem.

[62] Grabowski S. J. (2014). Tetrel
bond−σ-hole bond as a preliminary stage of the S_N_2 reaction. Phys. Chem. Chem. Phys..

[63] Ormond-Prout J. E., Smart P., Brammer L. (2012). Cyanometallates
as Halogen Bond Acceptors. Cryst. Growth Des..

[64] Christopherson J. C., Potts K. P., Bushuyev O. S., Topić F., Huskić I., Rissanen K., Barrett C. J., Friščić T. (2017). Assembly and
dichroism of a four-component halogen-bonded metalorganic cocrystal
salt solvate involving dicyanoaurate­(I) acceptors. Faraday Discuss..

[65] Imakubo T., Tajima N., Tamura M., Kato R., Nishiob Y., Kajitab K. (2002). A supramolecular superconductor θ-(DIETS)_2_[Au­(CN)_4_]. J. Mater. Chem..

[66] Cvrtila I., Stilinovic V. (2017). New Tricks by Old Anions: Hydrogen Bonded Hexacyanoferrous
Anionic Networks. Cryst. Growth Des..

[67] Boström H. L.
B., Collings I. E., Daisenberger D., Ridley C. J., Funnell N. P., Cairns A. B. (2021). Probing
the Influence of Defects, Hydration, and Composition
on Prussian Blue Analogues with Pressure. J.
Am. Chem. Soc..

[68] Streit T. N., Gomila R. M., Sievers R., Frontera A., Malischewski M. (2024). CF_3_-substituted sulfonium cations as efficient chalcogen bond donors
towards cyanometallates. CrystEngComm.

[69] Noland W. E., Schneerer A. K., Rabergea E. J., Tritch K. J. (2019). Crystal structure
of 2,3,5,6-tetrabromoterephthalonitrile. Acta
Crystallogr., Sect. E.

[70] Ishigaki Y., Asai K., Shimajiri T., Akutagawa T., Fukushima T., Suzuki T. (2021). Chalcogen Bond versus
Weak Hydrogen
Bond: Changing Contributions in Determining the Crystal Packing of
[1,2,5]-Chalcogenadiazole-Fused Tetracyanonaphthoquinodimethanes. Org. Mater..

[71] Bao M., Hayashi T., Shimada S. (2007). Cationic Organobismuth
Complex with
5,6,7,12-Tetrahydro-dibenz­[*c*,*f*]­[1,5]­azabismocine
Framework and Its Coordination Complexes with Neutral Molecules. Organometallics.

[72] Akkurt M., Özturk S., Kök T. R., Heinemann F. W. (2004). 2-(2,2-Dimethyl-1,3,2-dithiagermetan-4-ylidene)­malononitrile. Acta Crystallogr., Sect. E.

[73] Carlsson A.-C. C., Gräfenstein J., Budnjo A., Laurila J. L., Bergquist J., Karim A., Kleinmaier R., Brath U., Erdélyi M. (2012). Symmetric
Halogen Bonding Is Preferred
in Solution. J. Am. Chem. Soc..

[74] Wieske L. H. E., Erdelyi M. (2024). Halogen Bonds of Halogen­(I)
Ions—Where Are We
and Where to Go?. J. Am. Chem. Soc..

[75] Steiner T. (1997). Unrolling
the hydrogen bond properties of C–H···O interactions. Chem. Commun..

[76] Perkins C., Libri S., Adams H., Brammer L. (2012). Diiodoacetylene: compact,
strong ditopic halogen bond donor. CrystEngComm.

[77] Elliott M. C., Hughes C. E., Knowles P. J., Ward B. D. (2025). Alkyl groups in
organic molecules are NOT inductively electron-releasing. Org. Biomol. Chem..

[78] Schwerdtfeger P., Nagle J. K. (2019). 2018 Table of static
dipole polarizabilities of the neutral elements in the periodic table. Mol. Phys..

[79] Smith D. G. A., Burns L. A., Simmonett A. C., Parrish R. M., Schieber M. C., Galvelis R., Kraus P., Kruse H., Di Remigio R., Alenaizan A., James A. M., Lehtola S., Misiewicz J. P., Scheurer M., Shaw R. A., Schriber J. B., Xie Y., Glick Z. L., Sirianni D. A., O’Brien J. S., Waldrop J. M., Kumar A., Hohenstein E. G., Pritchard B. P., Brooks B. R., Schaefer H. F., Sokolov A. Y., Patkowski K., DePrince A. E., Bozkaya U., King R. A., Evangelista F. A., Turney J. M., Crawford T. D., Sherrill C. D. (2020). PSI4 1.4:
Open-source software for high-throughput quantum chemistry. J. Chem. Phys..

[80] Kroon J., Kanters J. A. (1974). Non-linearity of
hydrogen bonds in molecular crystals. Nature.

