# Supplementary material for: Nitrogen in Supramolecular Chemistry: Understanding the Pervasive Role as Acceptor of Halogen, Chalcogen, Pnictogen and Tetrel Bonds
Source: JACS Au. 2026 Sep 1;6(9):5163–78. doi: 10.1021/jacsau.6c00809 (PMC13625582; doi:10.1021/jacsau.6c00809)
Supplement: Supplementary file 1 [file au6c00809_si_001.pdf]

Supporting Information for "Nitrogen in supramolecular chemistry: Understanding the pervasive role as acceptor of halogen, chalcogen, pnictogen and tetrel bonds"

Harry. W. Nash,[1] Elna Pidcock,[2] Joanna S. Stevens,[2] J. Grant Hill,[,1] Lee Brammer*[,1]*

[1] *Chemistry Division, MPS School, University of Sheffield, Sheffield, S3 7HF, UK*

[2] *The Cambridge Crystallographic Data Centre, 12 Union Road, Cambridge, CB2 1EZ, UK*

Corresponding author emails: grant.hill@sheffield.ac.uk; lee.brammer@sheffield.ac.uk

Table of Contents

S1. Methods……………………………………………………………………………………………S2

S1.1 Geometric Data Retrieval from the CSD and Data Analysis ……………………………………S2

S1.2 Computational Methods……………………………………………………………………S3

S2. Interaction Prevalence Data……………………………………………………………………S5

S3. Mean Geometries for σ-Hole Interactions…………………………………………………………S9

S4. Geometric Results…………………………………………………………………………………S11

S4.1 σ-Hole Interactions with N_{sp^3} Acceptor Groups……………………………………………S11

S4.2 σ-Hole Interactions with N_{sp^2} Acceptor Groups……………………………………………S13

S4.3 σ-Hole Interactions with N_{sp} Acceptor Groups……………………………………………S16

S4.4 σ-Hole Donor Substituent Effects……………………………………………………………S18

S4.5 σ-Hole Interaction Energies and Geometries……………………………………………………S20

S5. SAPT σ-Hole Angle Scans……………………………………………………………………S22

S6. SAPT Lone Pair Angle Scans…………………………………………………………………S34

S7. References……………………………………………………………………………………S41

S1. METHODS

S1.1 Geometric Data Retrieval from the CSD and Data Analysis

In the Cambridge Structural database (CSD),[S1] each crystal structure is identified by a unique REFCODE. Searches of the CSD were used to retrieve the REFCODE of each structure meeting certain criteria (*vide infra*) used to define the presence of a σ-hole interaction alongside the required geometric data (distance and angles) associated with any σ-hole interactions of the type specified in a given search (Figure S1).

To search the Cambridge Structural Database (CSD), queries were created using the ConQuest[S2] interface and then saved as Connser format files to be used in searches of the database directed by the CSD Python API version 5.44.[S3] General search constraints applied were: crystallographic R-factor < 0.1, no disorder, no errors, no polymeric structures, and 3D coordinates determined. Interactions (E−X···N, E−Ch···N, E−Pn ···N and E−Tt···N) were considered if the interaction distance (*d*) was shorter than the sum of van der Waals (vdW) radii + 0.2 Å (i.e. $d < r_\sigma + r_N + 0.2$ Å), to account for longer range interactions. Interactions that were so short as to be classified as single bonds (i.e. hypervalency at the σ-hole donor) have not been included in our analyses. The measured interaction distance was then normalised with respect to the vdW radii of the donor and acceptor:

$$d_{norm} = \frac{d}{r_\sigma + r_N}$$

where d_{norm} is the normalised interaction distance, *d* is the measured interatomic distance, r_σ is the vdW radius of the σ-hole donor and r_N is the vdW radius of nitrogen. The vdW radii determined by Alvarez[S4] were used. Angle restrictions were imposed to define σ-hole interactions in the search queries, such that the σ-hole angle (θ_σ) and lone-pair angle (θ_{LP}), as defined in Figures 2 and 3, lie in the ranges $110° \le \theta_\sigma \le 180°$ and $110° \le \theta_{LP} \le 180°$, respectively. For each class of σ-hole donors, only those which satisfied their saturated valence, *i.e.*, only monovalent halogens, divalent chalcogens etc., were considered.

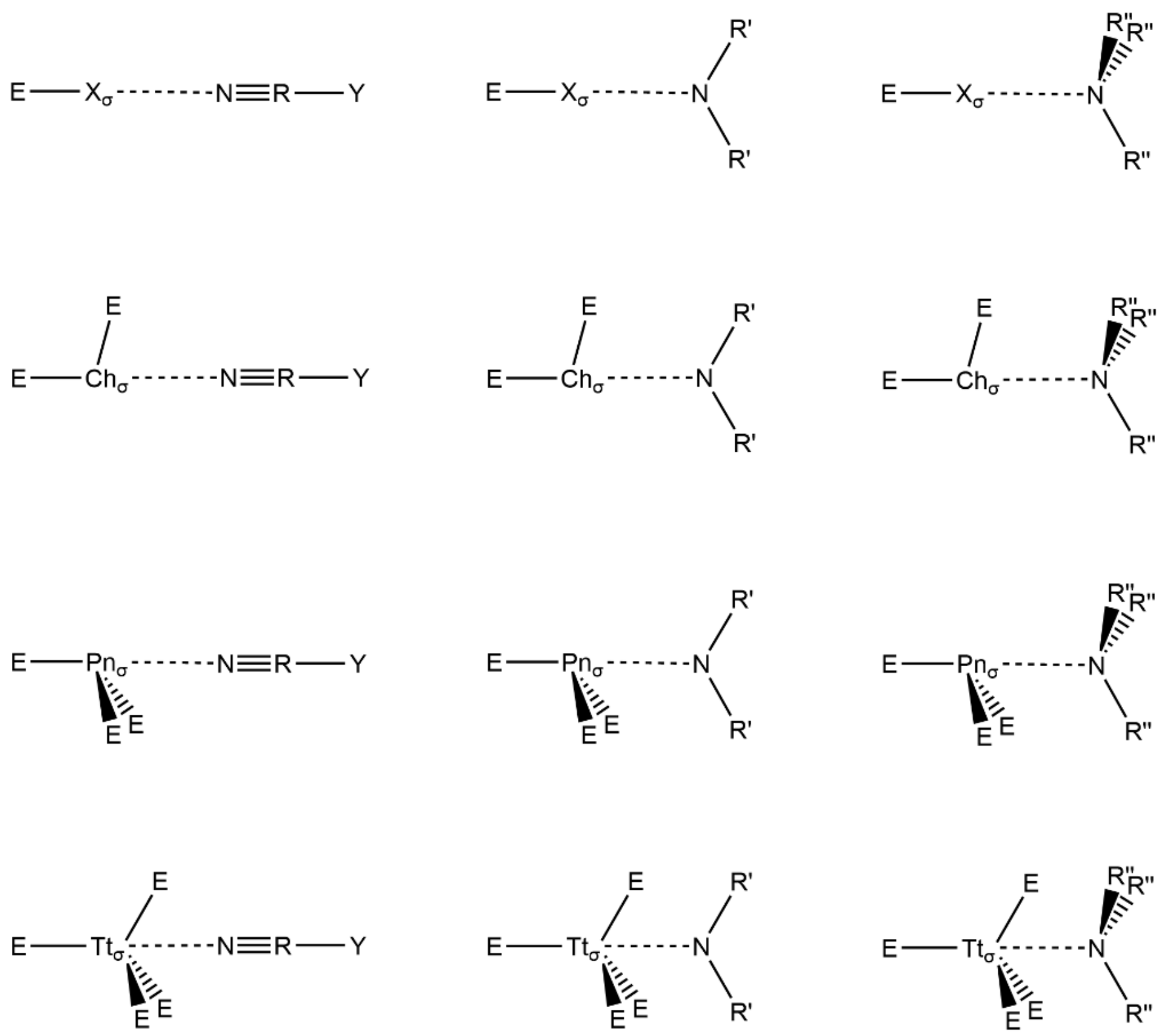

Figure S1. *Representation of the CSD queries used to find σ-hole interactions to nitrogen Lewis bases as the acceptor group. X_σ = Cl, Br, I; Ch_σ = S, Se, Te; Pn_σ = P, As, Sb, Bi; Tt_σ = Si, Ge, Sn, Pb; E = C, Si, Ge, N, P, As, Sb, O, S, Se, Te, F, Cl, Br, I; R = C, N, O, Si, P, S; R' = H, C, N, O, Si, P, S, B, Te, Cl, Br, I; R" = any non-metal element; Y = any element.*

A visual representation of the CSD search queries for halogen, chalcogen, pnictogen, and tetrel bond donor moieties interacting with N_{sp}, N_{sp^2}, and N_{sp^3} acceptor moieties is presented in Figure S1. For queries involving N_{sp} acceptor groups, the N−R bond was permitted to be either a triple or double bond and the R−Y bond was allowed to be of any bond type. For queries involving N_{sp^2} acceptor groups, the N−R' bond types were allowed to be double, aromatic or delocalised in the CSD classification. For queries involving N_{sp^3} acceptor groups, the N−R'' (R'' = non-metal) bond types were defined as single. For all queries, the bonds between σ-hole donor atom and their substituents (E) were defined to be single. To ensure that searches involving N_{sp^2} did not return structures with linear species (e.g. R−N=N=N) that would typically be considered sp-hybridised, species with R'−N−R' angle greater than 165 ° were removed. A similar precaution was taken in searches for N_{sp^3} acceptor groups results to avoid inclusion of planar trivalent N_{sp^2} species by excluding results in which the sum of the three R''−N− R'' angles exceeds 345°. To ensure accurate carbon hybridisation states were identified (Figure 13), the *Add-Hydrogens* function was applied using the CSD Python API to add hydrogen atoms omitted during the crystal structure determination process. Finally, where multiple contacts associated with the same REFCODE had identical θ_{LP} angle, only the shortest contact was retained.

To assess the prevalence of particular σ-hole interactions, the structural probability approach of Allen *et al.*[S5] was followed. Thus, the number of crystal structures that contain the appropriate σ-hole donor and acceptor moieties to form an interaction (S_{poss}) and the number of crystal structures in which that interaction occurs within our geometric criteria (S_{obs}) was determined, giving a prevalence (or probability of formation) as $P_S(\%) = 100 \times S_{obs}/S_{poss}$. To establish the prevalences for each type of σ-hole interaction a search of the CSD for crystal structures that contain the appropriate σ-hole donor and acceptor moieties to form the required interaction was conducted (S_{poss} = no. of REFCODEs). The S_{obs} values were taken from previous geometrically restricted searches. These values were then modified by retaining only one REFCODE from REFCODE families in which for some structures the six-letter REFCODE has a two-digit suffix to identify redeterminations or structures determined under different ambient conditions (p,T). The same post processing of N_{sp^2} and N_{sp^3} substituent angles (vide supra) to remove potential N_{sp} and N_{sp^2} structures, respectively, was also applied to these results for consistency. Where more than one σ-hole interaction type was present in a crystal structure, this was accounted for to avoid inaccuracies arising from multiple counting of structures which would otherwise arise in some of the statistics presented.

S1.2 Computational Methods

S1.2.1 Geometric optimisations and interaction energies

For all intermolecular complexes (42 σ-hole-donor/ σ-hole-acceptor pairs) used to establish trends in interaction energies, geometries were freely optimised using the M06-2X exchange correlation functional,[S6] including the D3(0) dispersion correction,[S7] and the def2-TZVPD basis set[S8,S9] using *ORCA* version 5.0.4 with the default defgrid2 integration grid.[S10] The geometry optimisations involving pnictogen and tetrel bonding complexes with acetonitrile were carried out using the *Molpro* package,[S11,S12] at the same level of theory, and were partially constrained in their optimisation with the C≡N···Pn/Tt angle fixed to 180°. Optimised geometries of all 42 intermolecular complexes are provided as a zipped archive via the University of Sheffield ORDA research data repository at http://doi.org/10.15131/shef.data.32270448. This density functional was selected as it has previously been shown to perform well in evaluating the strength of σ-hole interactions in the SH250×10 dataset.[S13]

Counterpoise-corrected interaction energies were then calculated at these geometries using the explicitly correlated local coupled-cluster method PNO-LCCSD(T*)-F12b[S14-S16] with the cc-pVDZ(-PP)-F12 basis set.[S17,S18] This level of theory is referred to as LCCSD(T*)-F12/cc-pVDZ-F12 herein, and the (T*) indicates that the triples correlation energy has been scaled by the ratio between MP2-F12 and F12, as evaluated for the interacting complexes.[S19,S20] All of these coupled-cluster calculations were carried out using the *Molpro* package of *ab initio* programs (version 2023.2). The heavier *p*-block elements (Br, I, Se, Te, As, Sb, Bi, Ge, Sn, and Pb) used the cc-pVDZ-PP-F12 basis along with small-core relativistic pseudo-potentials. The matching auxiliary basis sets of the JKFit, MP2Fit, and OptRI varieties (using the Molpro naming conventions) were also used.[S18,S21-S25] Tight domains were used in the local correlation treatment,[S15] and the complementary auxiliary basis set (CABS) singles correction, evaluated without local approximations, was added to the Hartree-Fock energies.[S26] The 3*A(FIX) Ansatz was used in the explicit

correlation, where the (n – 1)d electrons were included in the correlation treatment (a geminal exponent of 1.0 a_0^{-1} was used for the valence-valence pairs and 1.4 a_0^{-1} for all other pairs).[S27,S28]

S1.2.2 SAPT interaction energy analysis

The underlying nature of the intermolecular interactions in terms of electrostatic, induction, exchange-repulsion, and dispersion contributions to the overall interaction energy was evaluated using symmetry-adapted perturbation theory (SAPT).[S29] In particular, the SAPT2 level of theory was used, along with the def2-TZVPD basis set, using the Psi4 version 1.6.1 package.[S30] For the selected σ-hole donors and acceptors, SAPT energies were calculated along a scan of the σ-hole angle (θ_σ), ranging from 95-180°, with the donors and acceptors held fixed in their respective interacting geometries. Separate scans, over the same ranges, were carried out for lone-pair angles (θ_{LP}). Angle scans were completed for the same range of angles in all cases, for comparative purposes, even if the resulting structures have some close intermolecular distances.

S1.2.3 – Molecular Electrostatic Surface Potential Maps

The molecular electrostatic potential maps of Figure 1 were created in WebMO (version 25.0.008e)[S31] using the interface to Gaussian 16 (version C.01).[S32] The M06-2X exchange-correlation functional was used in the required DFT calculations. As an all-electron basis set was required to obtain physically reasonable values of the potential, the DZVP basis set was used for all elements.[S33]

S2. Interaction Prevalence Data

Table S1. Numbers of contacts and interaction prevalence data for halogen, chalcogen, pnictogen and tetrel bonds as a function of nitrogen atom hybridisation of σ-hole acceptor

**Number of interactions**				
	**N_{sp}**	**N_{sp^2}**	**N_{sp^3}**	**All N**
**Total**	3873	11556	927	16356
**% of total**	23.68	70.65	5.67	100
**X**	1571	6002	707	8280
**Ch**	2155	5302	153	7610
**Pn**	41	52	25	118
**Tt**	106	200	42	348
**Number of structures containing σ-hole donor and acceptor moieties (S_{poss})**				
	**N_{sp}**	**N_{sp^2}**	**N_{sp^3}**	**All N**
**Total**	9848	42191	9574	56797
**X**	5519	27776	6340	36400
**Ch**	3968	13562	2277	18225
**Pn**	236	855	406	1453
**Tt**	738	2843	1056	4418
**Number of unique structures containing σ-hole interactions (S_{obs})**				
	**N_{sp}**	**N_{sp^2}**	**N_{sp^3}**	**All N**
**Total**	2249	6934	675	9689
**X**	1032	4055	499	5540
**Ch**	1157	2853	135	4031
**Pn**	29	33	15	75
**Tt**	74	124	30	226
**Prevalence (%)**				
	**N_{sp}**	**N_{sp^2}**	**N_{sp^3}**	**All N**
**Total**	22.84	16.43	7.05	17.06
**X**	18.70	14.60	7.87	15.22
**Ch**	29.16	21.04	5.93	22.12
**Pn**	12.29	3.86	3.69	5.16
**Tt**	10.03	4.36	2.84	5.12

Table S2. Numbers of contacts and interaction prevalence data for halogen bonds, listed halogen-bond donor atoms Cl, Br, I as a function of nitrogen atom hybridisation of σ-hole acceptor

**Number of interactions**				
	**N_{sp}**	**N_{sp^2}**	**N_{sp^3}**	**All N**
**Total (X)**	1571	6002	707	8280
**% of total**	18.97	72.49	8.54	100
**Cl**	776	2764	263	3803
**Br**	455	1172	153	1780
**I**	340	2066	291	2697
**Number of structures containing halogen-bond donor and acceptor moieties (S_{poss})**				
	**N_{sp}**	**N_{sp^2}**	**N_{sp^3}**	**All N**
**Total (X)**	5519	27776	6340	36400
**Cl**	3378	18662	4060	23871
**Br**	1808	7511	1984	10432
**I**	482	2614	480	3364
**Number of structures containing halogen-bond interactions (S_{obs})**				
	**N_{sp}**	**N_{sp^2}**	**N_{sp^3}**	**All N**
**Total (X)**	1032	4055	499	5540
**Cl**	521	1959	217	2670
**Br**	326	863	125	1305
**I**	188	1265	159	1603

Prevalence (%)				
	N_{sp}	N_{sp}^2	N_{sp}^3	All N
Total (X)	18.70	14.60	7.87	15.22
Cl	15.42	10.50	5.34	11.19
Br	18.03	11.49	6.30	12.51
I	39.00	48.39	33.13	47.65

Table S3. Numbers of contacts and interaction prevalence data for chalcogen bonds, listed chalcogen-bond donor atoms S, Se, Te as a function of nitrogen atom hybridisation of σ-hole acceptor

Number of interactions				
	N_{sp}	N_{sp}^2	N_{sp}^3	All N
Total (Ch)	2155	5302	153	7610
% of total	28.32	69.67	2.01	100
S	1899	4545	141	6585
Se	238	586	7	831
Te	18	171	5	194
Number of structures containing chalcogen-bond donor and acceptor moieties (S_{poss})				
	N_{sp}	N_{sp}^2	N_{sp}^3	All N
Total (Ch)	3968	13562	2277	18225
S	3691	12814	2128	17124
Se	294	669	143	1033
Te	34	136	19	183
Number of structures containing chalcogen-bond interactions (S_{obs})				
	N_{sp}	N_{sp}^2	N_{sp}^3	All N
Total (Ch)	1157	2853	135	4031
S	1025	2466	127	3527
Se	126	313	6	427
Te	13	94	2	108
Prevalence (%)				
	N_{sp}	N_{sp}^2	N_{sp}^3	All N
Total (Ch)	29.16	21.04	5.93	22.12
S	27.77	19.24	5.97	20.60
Se	42.86	46.79	4.20	41.34
Te	38.24	69.12	10.53	59.02

Table S4. Numbers of contacts and interaction prevalence data for pnictogen bonds, listed pnictogen-bond donor atoms P, As, Sb, Bi as a function of nitrogen atom hybridisation of σ-hole acceptor

Number of interactions				
	N_{sp}	N_{sp}^2	N_{sp}^3	All N
Total (Pn)	41	52	25	118
% of total	34.75	44.07	21.19	100
P	17	12	15	44
As	10	8	1	19
Sb	6	23	8	37
Bi	8	9	1	18
Number of structures containing pnictogen-bond donor and acceptor moieties (S_{poss})				
	N_{sp}	N_{sp}^2	N_{sp}^3	All N
Total (Pn)	236	855	406	1453
P	204	755	321	1245
As	17	36	18	67
Sb	11	41	46	95
Bi	8	23	24	53
Number of structures containing pnictogen-bond interactions (S_{obs})				
	N_{sp}	N_{sp}^2	N_{sp}^3	All N
Total (Pn)	29	33	15	75
P	14	9	12	34
As	5	7	1	12

**Sb**	5	11	1	17
**Bi**	5	6	1	12
**Prevalence (%)**				
	**N_{sp}**	**N_{sp^2}**	**N_{sp^3}**	**All N**
**Total (Pn)**	12.29	3.86	3.69	5.16
**P**	6.86	1.19	3.74	2.73
**As**	29.41	19.44	5.56	17.91
**Sb**	45.45	26.83	2.17	17.89
**Bi**	62.50	26.09	4.17	22.64

Table S5. Numbers of contacts and interaction prevalence data for tetrel bonds, listed tetrel-bond donor atoms Si, Ge, Sn, Pb as a function of nitrogen atom hybridisation of σ-hole acceptor

**Number of interactions**				
	**N_{sp}**	**N_{sp^2}**	**N_{sp^3}**	**All N**
**Total (Tt)**	106	200	42	348
**% of total**	30.46	57.47	12.07	100
**Si**	65	70	30	165
**Ge**	11	1	4	16
**Sn**	26	66	8	100
**Pb**	4	63	0	67
**Number of structures containing tetrel-bond donor and acceptor moieties (S_{poss})**				
	**N_{sp}**	**N_{sp^2}**	**N_{sp^3}**	**All N**
**Total (Tt)**	738	2843	1056	4418
**Si**	654	2452	884	3785
**Ge**	40	53	58	150
**Sn**	38	300	117	445
**Pb**	15	60	8	80
**Number of structures containing tetrel-bond interactions (S_{obs})**				
	**N_{sp}**	**N_{sp^2}**	**N_{sp^3}**	**All N**
**Total (Tt)**	74	124	30	226
**Si**	45	44	21	110
**Ge**	7	1	3	11
**Sn**	18	49	6	71
**Pb**	4	30	0	34
**Prevalence (%)**				
	**N_{sp}**	**N_{sp^2}**	**N_{sp^3}**	**All N**
**Total (Tt)**	10.03	4.36	2.84	5.12
**Si**	6.88	1.79	2.38	2.91
**Ge**	17.50	1.89	5.17	7.33
**Sn**	47.37	16.33	5.13	15.96
**Pb**	26.67	50.00	0.00	42.50

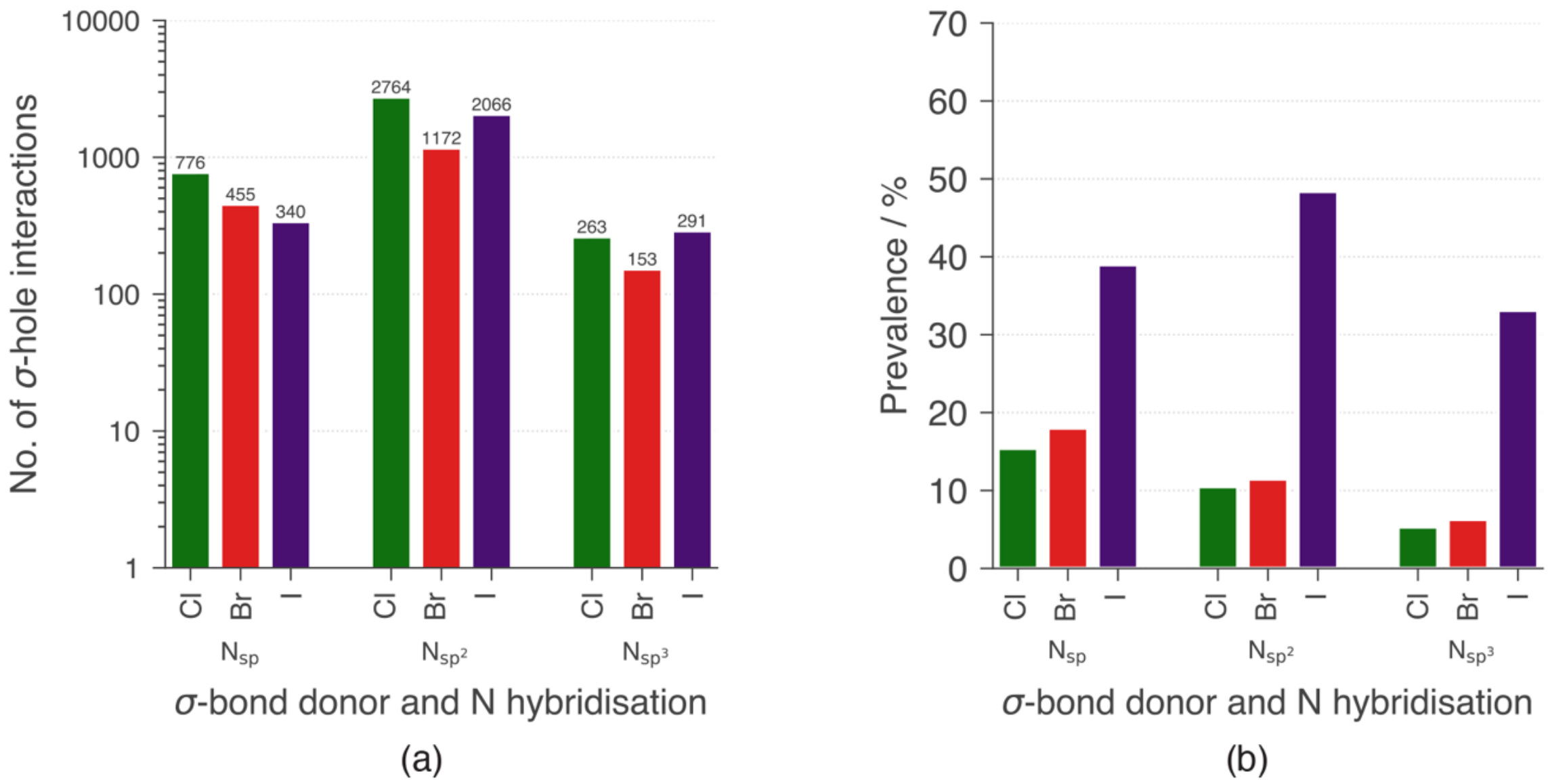

Figure S2. (a) Number of σ-hole interactions (logarithmic scale) for each halogen-bond donor group as a function of nitrogen atom hybridisation of σ-hole acceptor. (b) Prevalence of crystal structures with a halogen-bond interaction as proportion of crystal structures that contain the necessary moieties to form such an interaction.

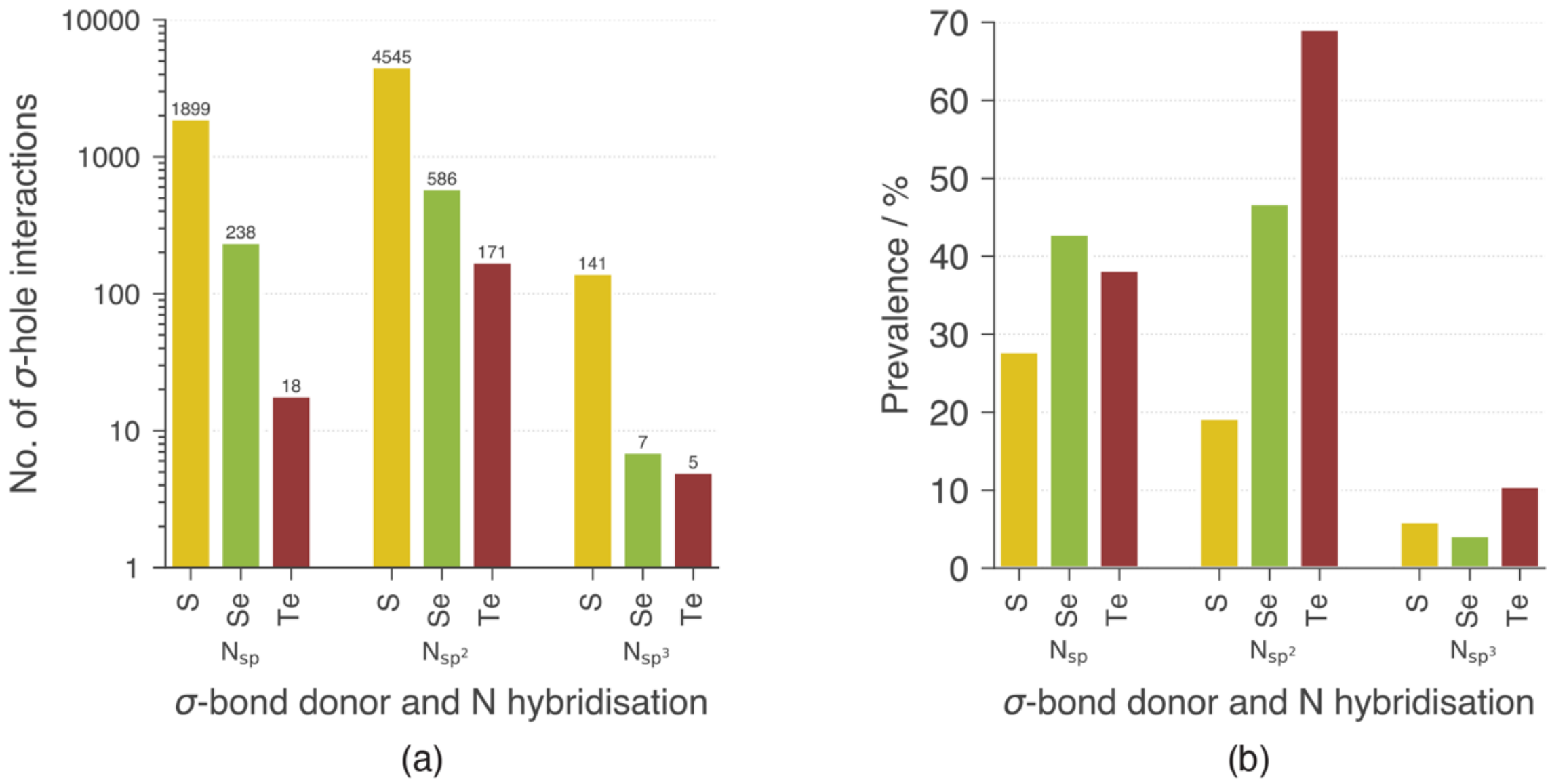

Figure S3. (a) Number of σ-hole interactions (logarithmic scale) for each chalcogen-bond donor group as a function of nitrogen atom hybridisation of σ-hole acceptor. (b) Prevalence of crystal structures with a chalcogen-bond interaction as proportion of crystal structures that contain the necessary moieties to form such an interaction.

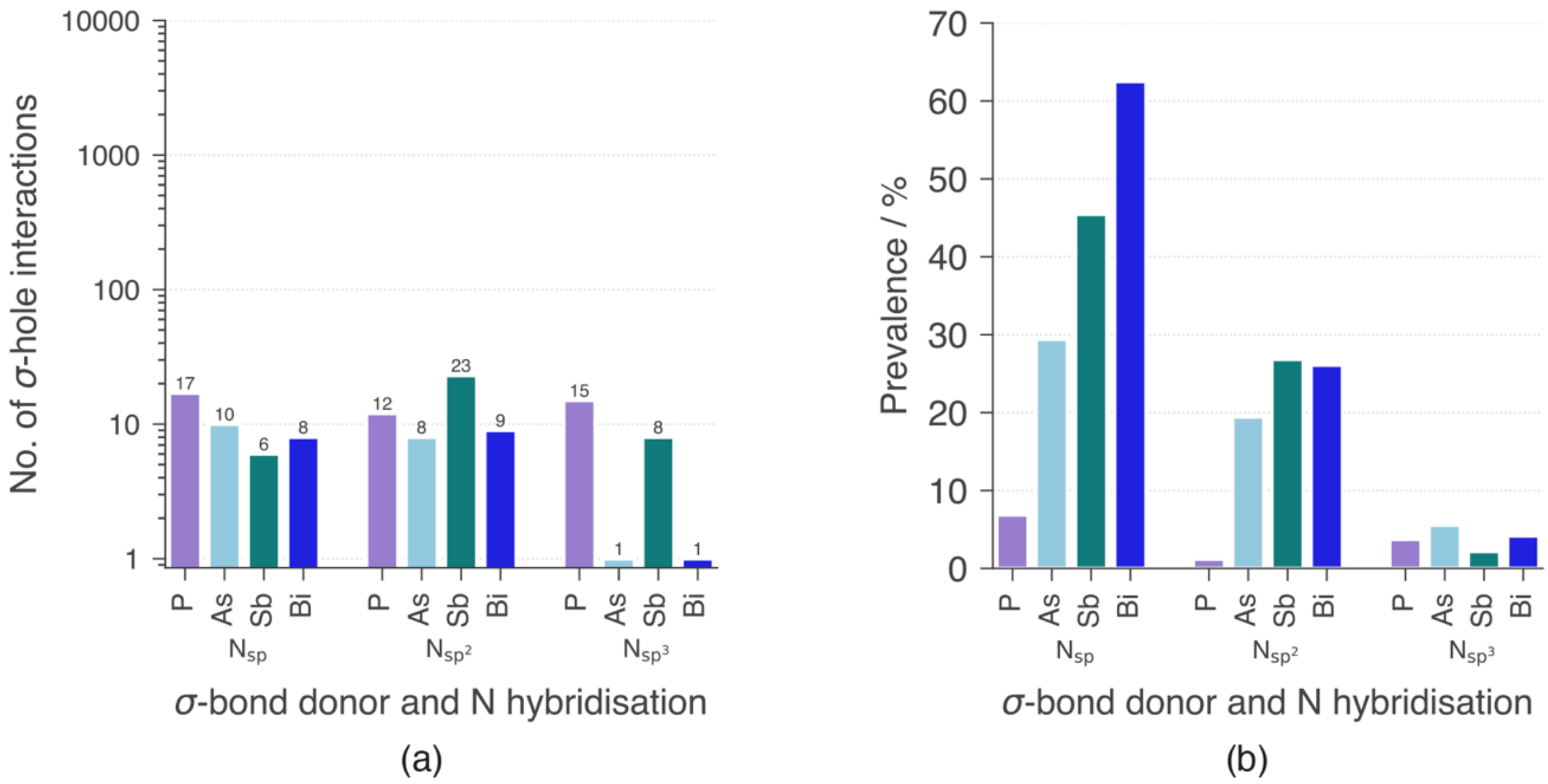

Figure S4. (a) Number of σ-hole interactions (logarithmic scale) for each pnictogen-bond donor group as a function of nitrogen atom hybridisation of σ-hole acceptor. (b) Prevalence of crystal structures with a pnictogen-bond interaction as proportion of crystal structures that contain the necessary moieties to form such an interaction.

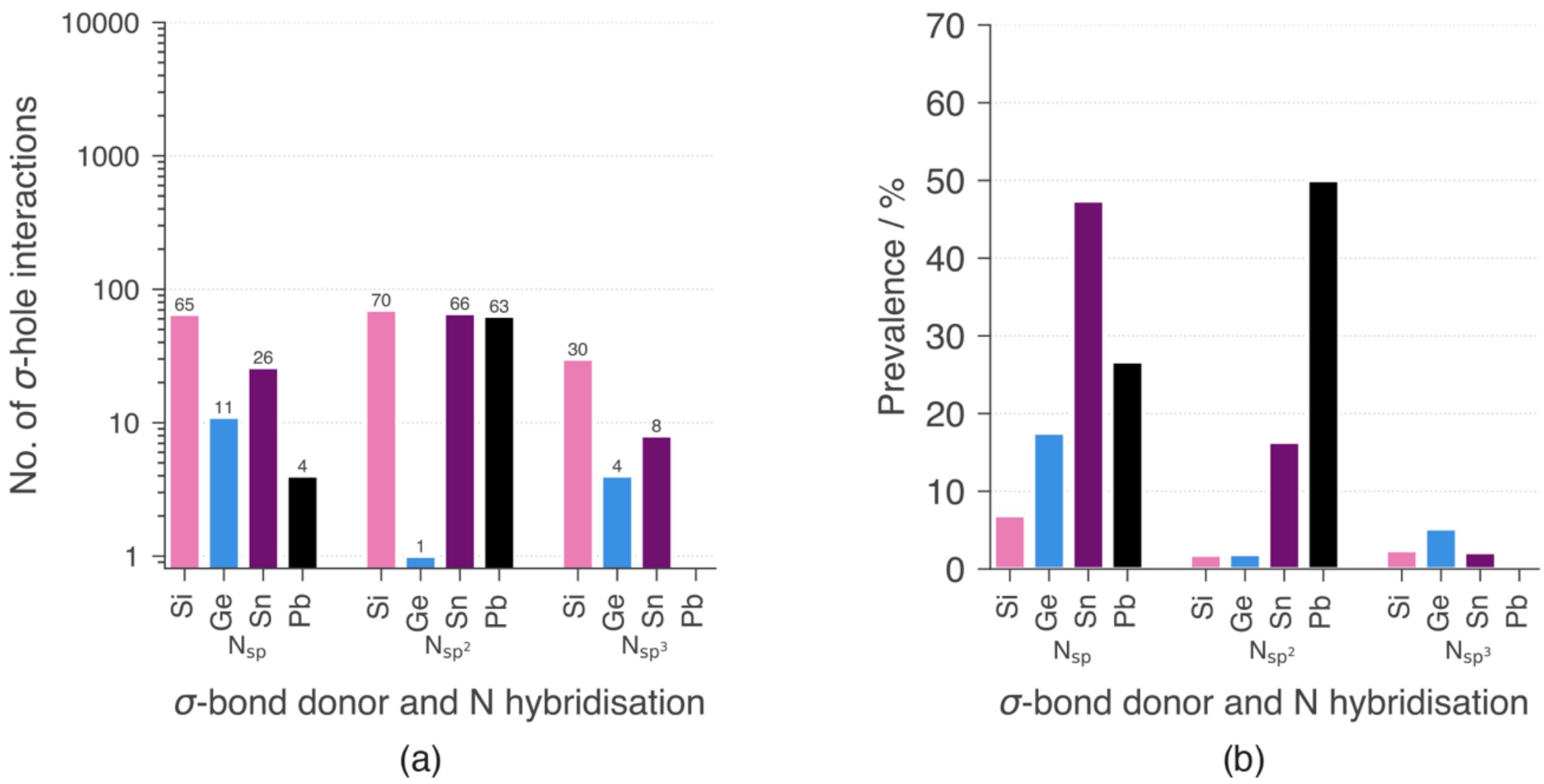

Figure S5. (a) Number of σ-hole interactions (logarithmic scale) for each tetrel-bond donor group as a function of nitrogen atom hybridisation of σ-hole acceptor. (b) Prevalence of crystal structures with a tetrel-bond interaction as proportion of crystal structures that contain the necessary moieties to form such an interaction.

S3. Mean Geometries for σ-Hole Interactions

Table S6. Mean geometries for σ-hole interactions involving N_{sp^3} acceptor groups, including σ-hole donor atoms where there are fewer than 5 observations (red font).

Donor – Acceptor ($D_σ$···N)	Mean d_{norm}	Mean $D_σ$···N distance (Å)	Mean $θ_σ$ angle (°)	Mean $θ_{LP}$ angle (°)	Number of observations
Cl···N_{sp^3}	0.991	3.449	144	145	263
Br···N_{sp^3}	0.918	3.232	157	154	153
I···N_{sp^3}	0.791	2.927	170	165	291

S···N_{sp^3}	0.996	3.535	147	141	141
Se···N_{sp^3}	0.886	3.084	170	149	7
Te···N_{sp^3}	0.796	2.907	160	168	5
P···N_{sp^3}	1.001	3.562	154	149	15
As···N_{sp^3}	0.802	2.839	169	156	1
Sb···N_{sp^3}	0.854	3.527	157	136	8
Bi···N_{sp^3}	1.029	4.321	150	132	1
Si···N_{sp^3}	1.004	3.865	140	157	30
Ge···N_{sp^3}	1.009	3.984	156	131	4
Sn···N_{sp^3}	0.860	3.510	161	150	8
Pb···N_{sp^3}	–	–	–	–	0

Table S7. Mean geometries for σ-hole interactions involving N_{sp^2} acceptor groups, including σ-hole donor atoms where there are fewer than 5 observations (red font).

Donor – Acceptor ($D_σ$···N)	Mean d_{norm}	Mean $D_σ$···N distance (Å)	Mean $θ_σ$ angle (°)	Mean $θ_{LP}$ angle (°)	Number of observations
Cl···N_{sp^2}	0.976	3.379	149	140	2764
Br···N_{sp^2}	0.934	3.288	158	146	1172
I···N_{sp^2}	0.815	3.017	170	161	2066
S···N_{sp^2}	0.955	3.392	152	139	4545
Se···N_{sp^2}	0.890	3.097	159	145	586
Te···N_{sp^2}	0.841	3.071	160	150	171
P···N_{sp^2}	1.012	3.603	155	138	12
As···N_{sp^2}	0.943	3.337	159	146	8
Sb···N_{sp^2}	0.812	3.352	162	147	23
Bi···N_{sp^2}	0.883	3.707	162	147	9
Si···N_{sp^2}	1.005	3.868	145	153	70
Ge···N_{sp^2}	1.019	4.027	152	155	1
Sn···N_{sp^2}	0.790	3.225	162	158	66
Pb···N_{sp^2}	0.766	3.262	153	134	63

Table S8. Mean geometries for σ-hole interactions involving N_{sp} acceptor groups, including σ-hole donor atoms where there are fewer than 5 observations (red font).

Donor – Acceptor ($D_σ$···N)	Mean d_{norm}	Mean $D_σ$···N distance (Å)	Mean $θ_σ$ angle (°)	Mean $θ_{LP}$ angle (°)	Number of observations
Cl···N_{sp}	0.950	3.305	155	136	776
Br···N_{sp}	0.929	3.270	159	136	455
I···N_{sp}	0.884	3.271	161	140	340
S···N_{sp}	0.948	3.365	155	136	1899
Se···N_{sp}	0.920	3.203	163	144	238
Te···N_{sp}	0.912	3.327	156	137	18
P···N_{sp}	0.958	3.409	162	140	17
As···N_{sp}	0.845	2.993	166	154	10
Sb···N_{sp}	0.759	3.134	161	147	6
Bi···N_{sp}	0.803	3.260	164	132	8

Si···N_{sp}	0.925	3.563	171	138	65
Ge···N_{sp}	0.873	3.447	160	153	11
Sn···N_{sp}	0.800	3.266	163	148	26
Pb···N_{sp}	0.763	3.252	149	119	4

S4. Geometric Results

S4.1 σ-Hole Interactions with N_{sp}^3 Acceptor Groups

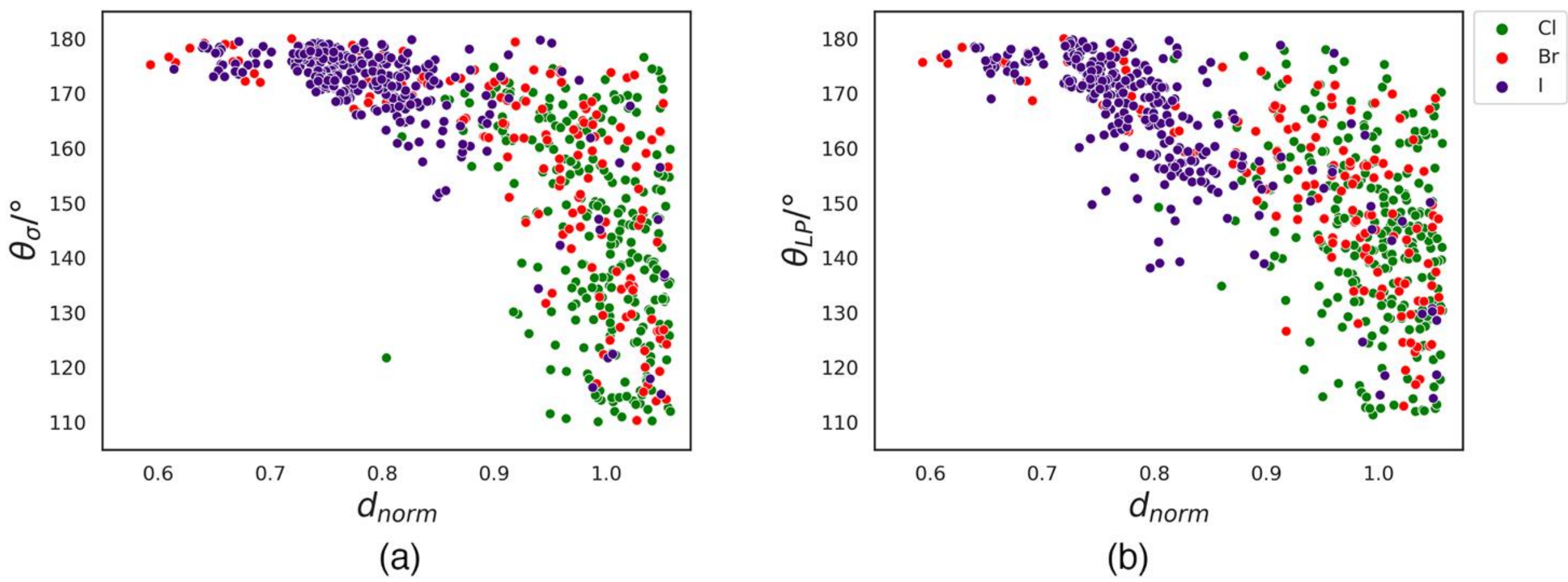

Figure S6. Distribution of geometries for halogen bonds involving N_{sp}^3 acceptor groups. (a) Normalised X···N distance (d_{norm}) vs. E−X···N σ-hole angle, θ_{σ}. (b) Normalised X···N distance(d_{norm}) vs. N_{sp}^3 lone-pair angle, θ_{LP}. Data points are colour-coded by halogen-bond donor atom.

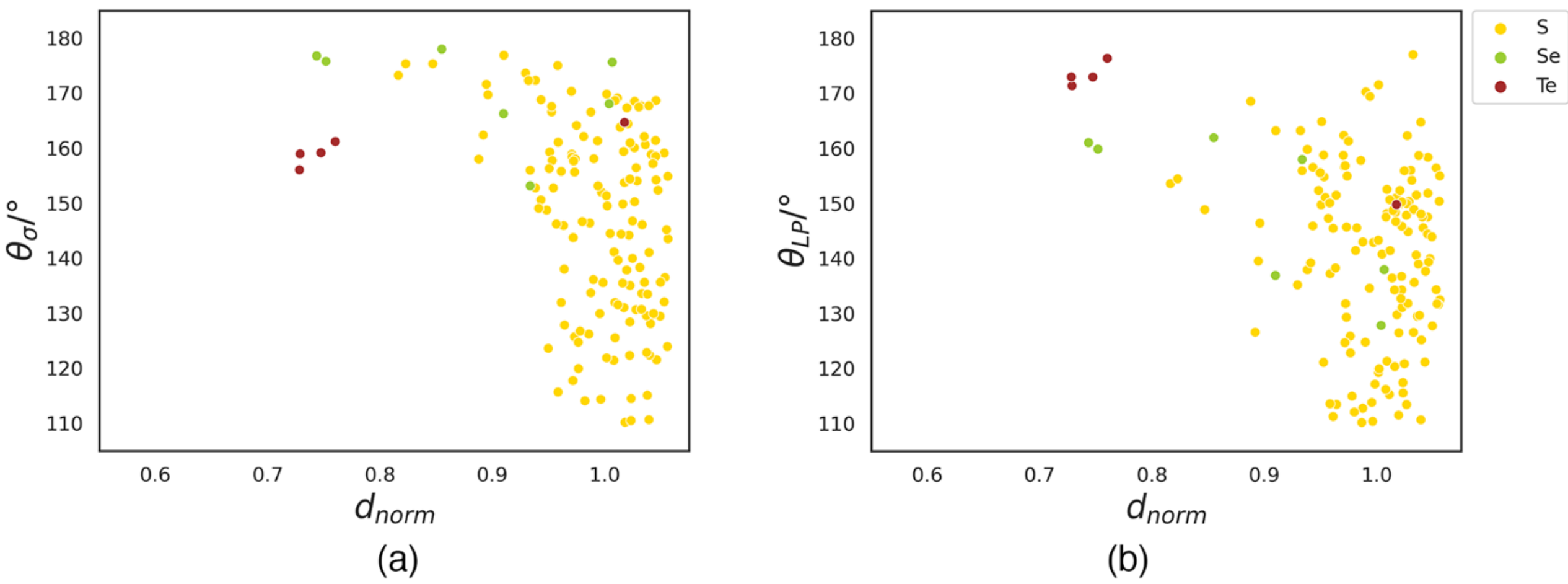

Figure S7. Distribution of geometries for chalcogen bonds involving N_{sp}^3 acceptor groups. (a) Normalised Ch···N distance (d_{norm}) vs. E−Ch···N σ-hole angle, θ_{σ}. (b) Normalised Ch···N distance (d_{norm}) vs. N_{sp}^3 lone-pair angle, θ_{LP}. Data points are colour-coded by chalcogen-bond donor atom.

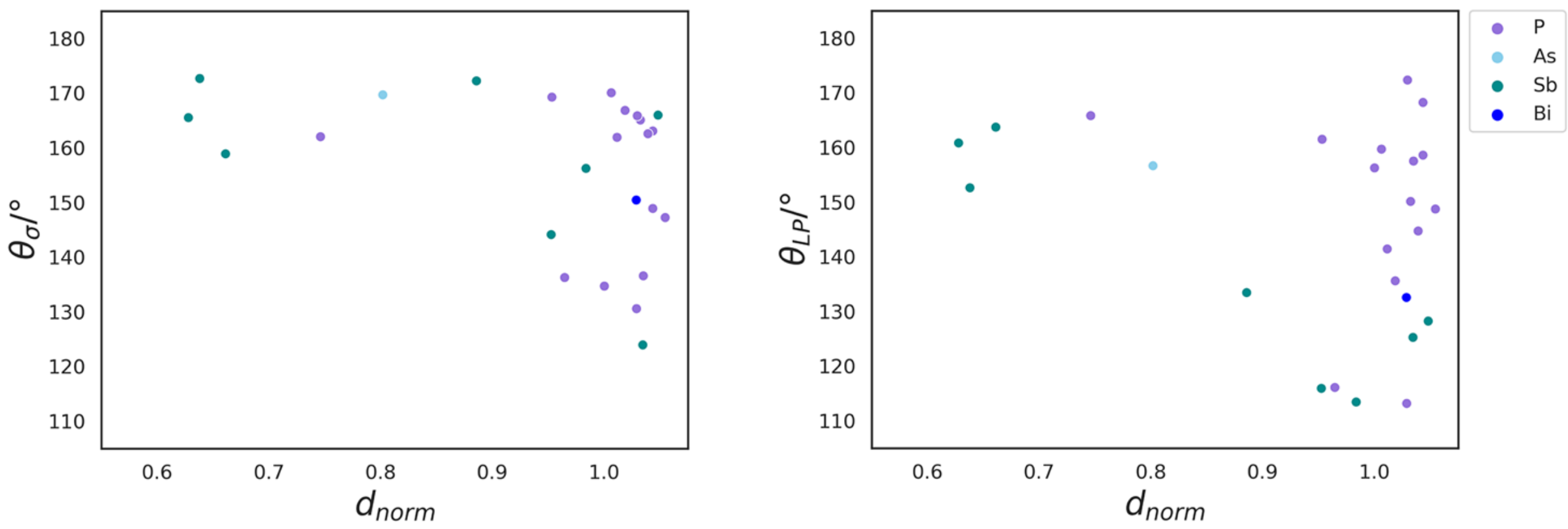

(a) (b)

Figure S8. Distribution of geometries for pnictogen bonds involving N_{sp^3} acceptor groups. (a) Normalised Pn···N distance (d_{norm}) vs. E−Pn···N σ-hole angle, θ_{σ}. (b) Normalised Pn···N distance (d_{norm}) vs. N_{sp^3} lone-pair angle, θ_{LP}. Data points are colour-coded by pnictogen-bond donor atom.

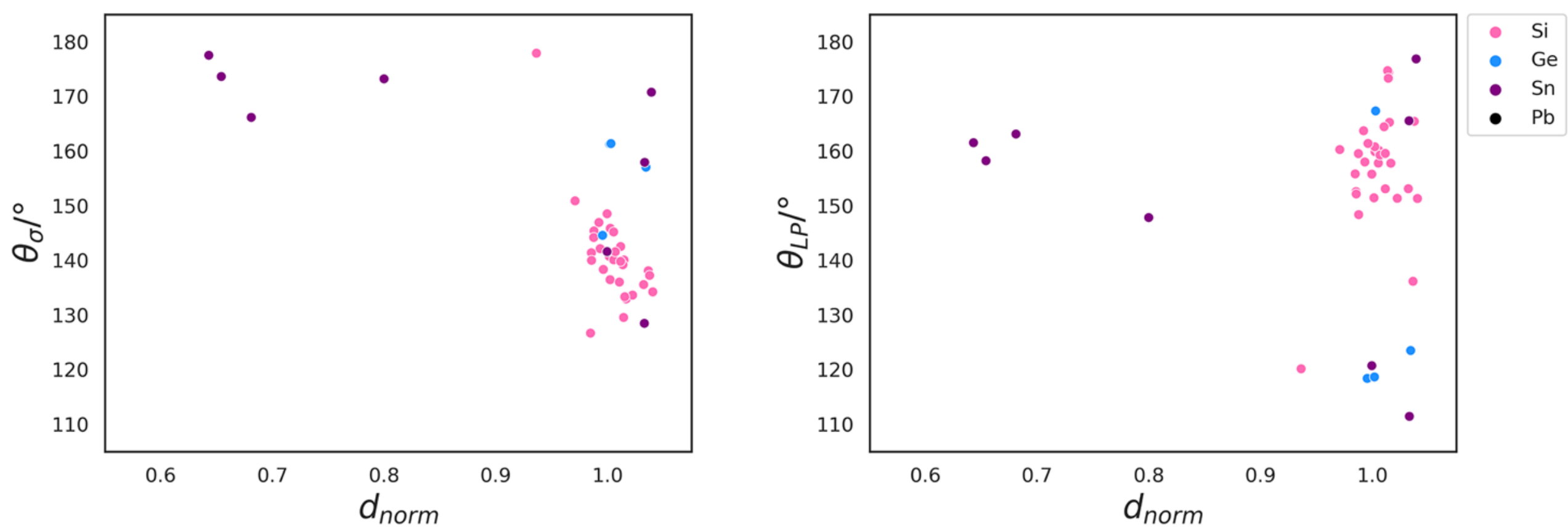

(a) (b)

Figure S9. Distribution of geometries for tetrel bonds involving N_{sp^3} acceptor groups. (a) Normalised Tt···N distance (d_{norm}) vs. E−Tt···N σ-hole angle, θ_{σ}. (b) Normalised Tt···N distance (d_{norm}) vs. N_{sp^3} lone-pair angle, θ_{LP}. Data points are colour-coded by tetrel-bond donor atom.

S4.2 σ-Hole Interactions with N_{sp}^2 Acceptor Groups

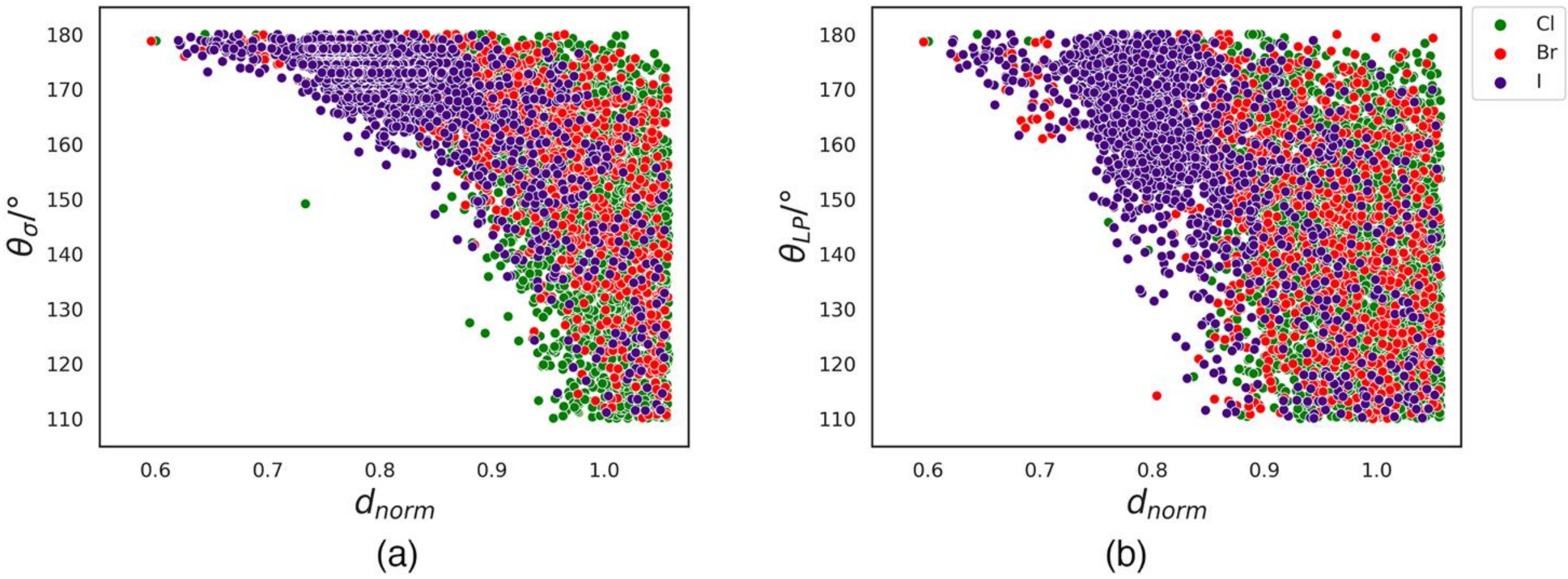

Figure S10. Distribution of geometries for halogen bonds involving N_{sp}^2 acceptor groups. (a) Normalised X···N distance (d_{norm}) vs. E−X···N σ-hole angle, θ_σ. (b) Normalised X···N distance(d_{norm}) vs. N_{sp}^2 lone-pair angle, θ_{LP}. Data points are colour-coded by halogen-bond donor atom.

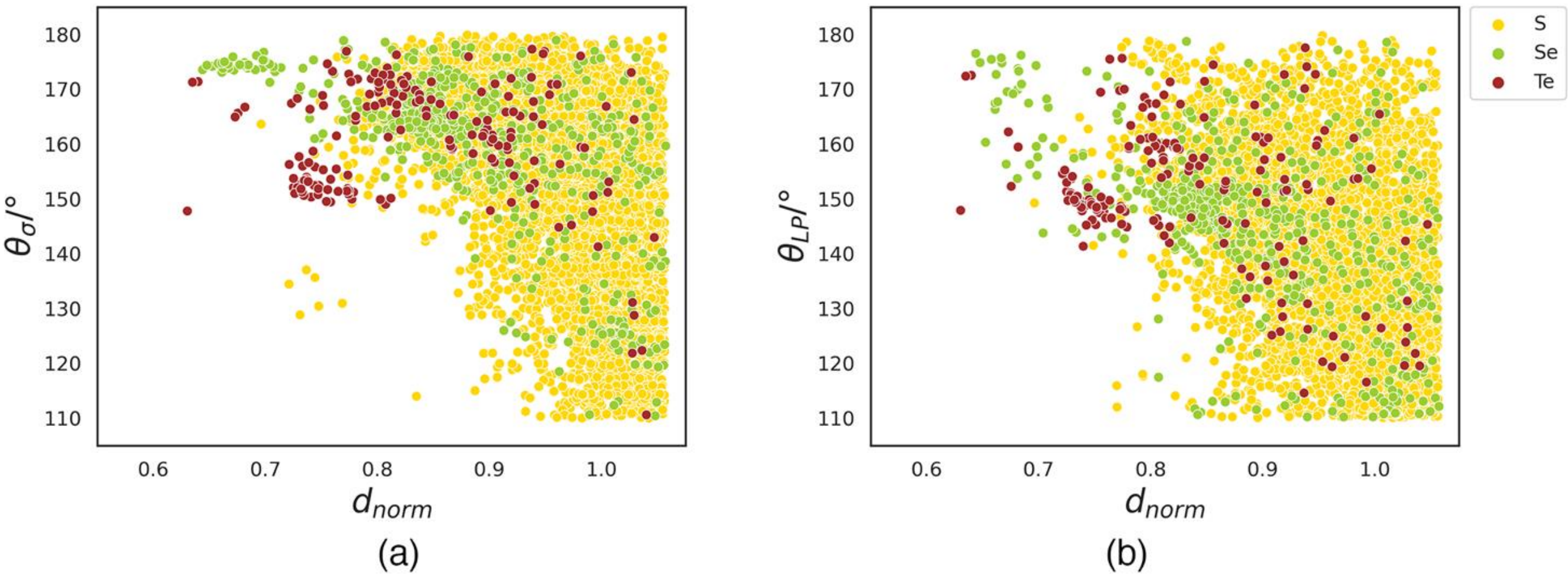

Figure S11. Distribution of geometries for chalcogen bonds involving N_{sp}^2 acceptor groups. (a) Normalised Ch···N distance (d_{norm}) vs. E−Ch···N σ-hole angle, θ_σ. (b) Normalised Ch···N distance (d_{norm}) vs. N_{sp}^2 lone-pair angle, θ_{LP}. Data points are colour-coded by chalcogen-bond donor atom.

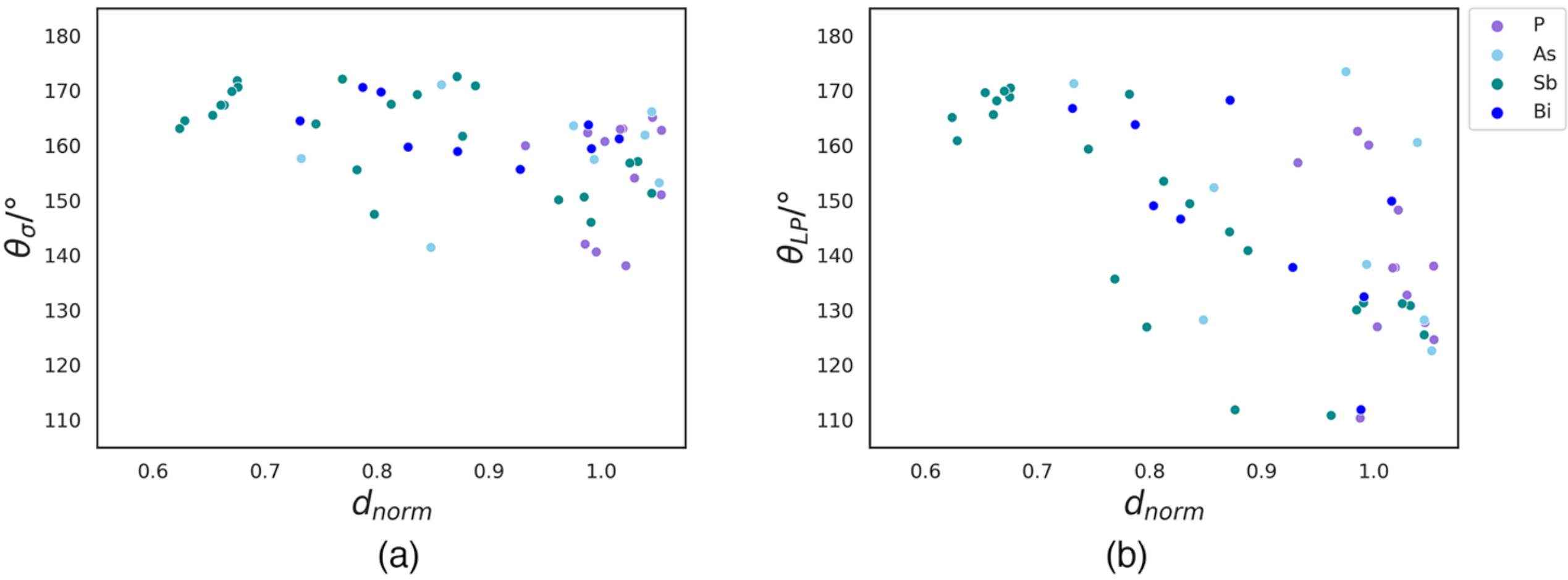

Figure S12. Distribution of geometries for pnictogen bonds involving N_{sp}^2 acceptor groups. (a) Normalised Pn···N distance (d_{norm}) vs. E−Pn···N σ-hole angle, θ_σ. (b) Normalised Pn···N distance (d_{norm}) vs. N_{sp}^2 lone-pair angle, θ_{LP}. Data points are colour-coded by pnictogen-bond donor atom.

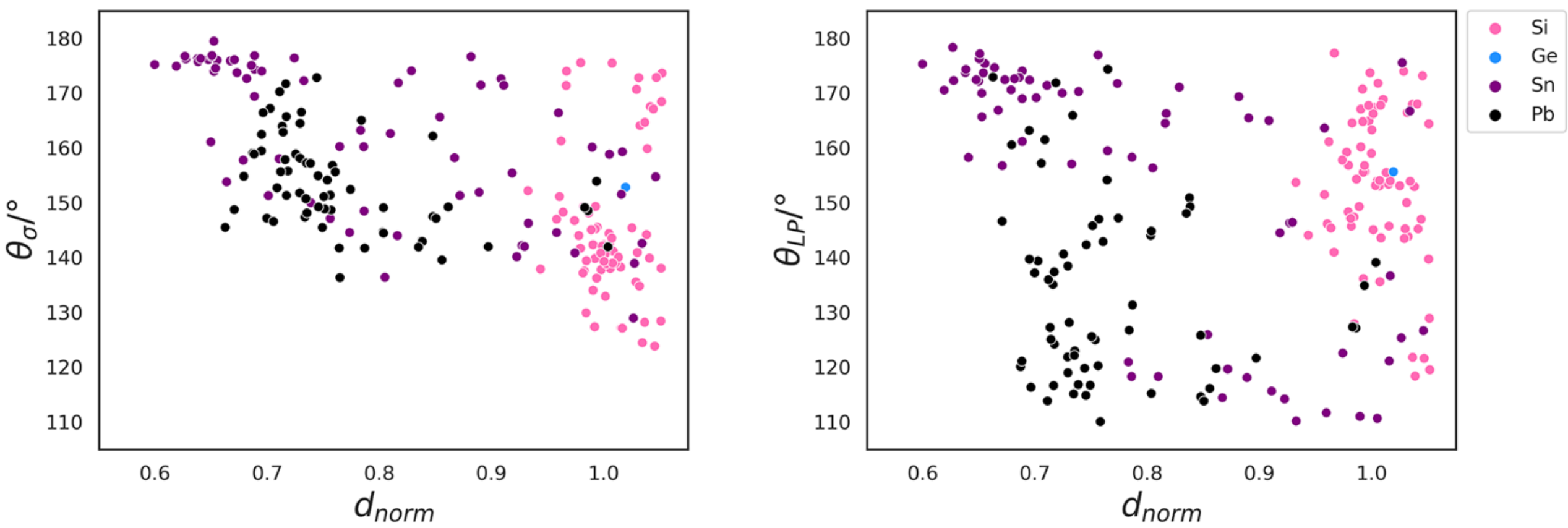

(a) (b)

Figure S13. Distribution of geometries for tetrel bonds involving N_{sp^2} acceptor groups. (a) Normalised Tt···N distance (d_{norm}) vs. E−Tt···N σ-hole angle, θ_σ. (b) Normalised Tt···N distance (d_{norm}) vs. N_{sp^2} lone-pair angle, θ_{LP}. Data points are colour-coded by tetrel-bond donor atom.

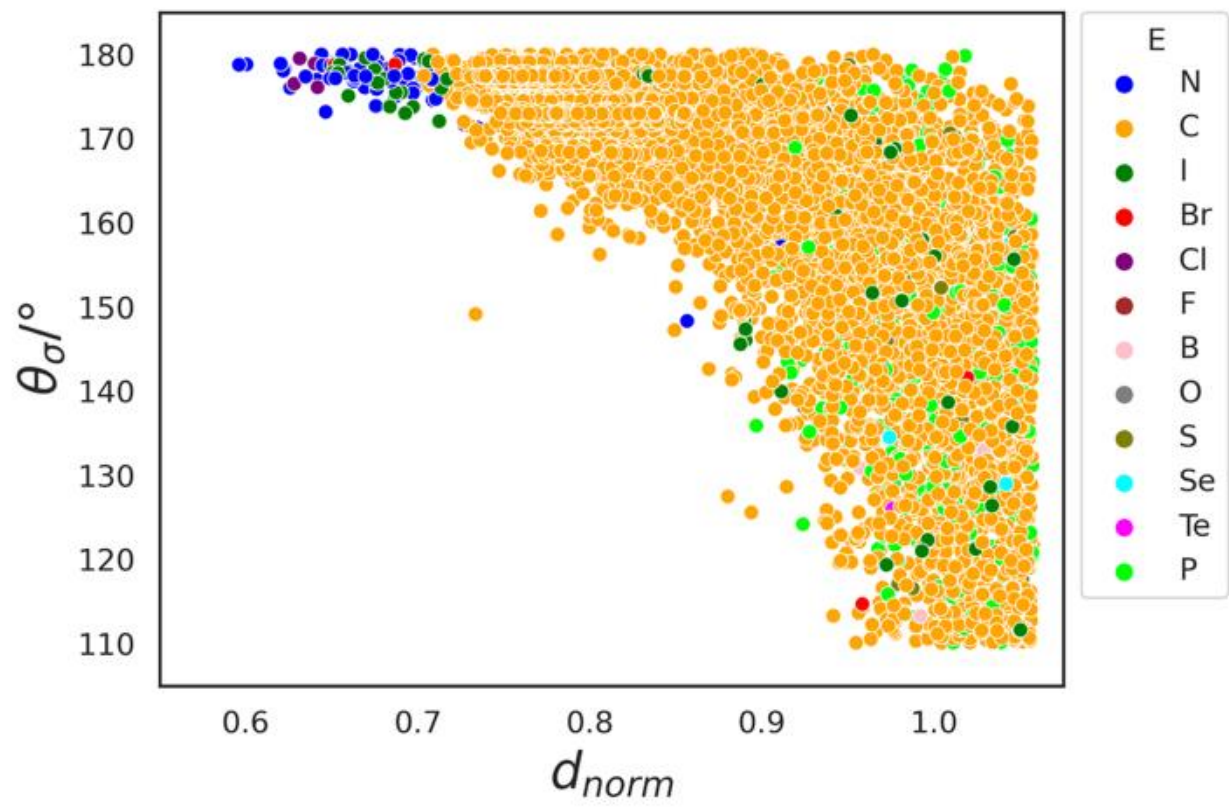

Figure S14. Distribution of geometries for halogen bonds involving N_{sp^2} acceptor groups represented as normalised X···N distance (d_{norm}) vs. E−X···N σ-hole angle, θ_σ. Data points are colour-coded by substituent (E) on the halogen-bond donor atom.

Table S6. Number of halogen-bond and chalcogen-bond contacts involving N_{sp^2} acceptor groups in N-heterocycles

N-heterocycle ring size	No. of halogen-bonding interactions	No. of chalcogen-bonding interactions
3	2	1
4	7	1
5	1704	3782
6	3487	1064
7	27	38
8	35	34
9	0	0
10	6	1
11	0	0
12	11	1
Other	281	197
**Total**	**5560**	**5119**

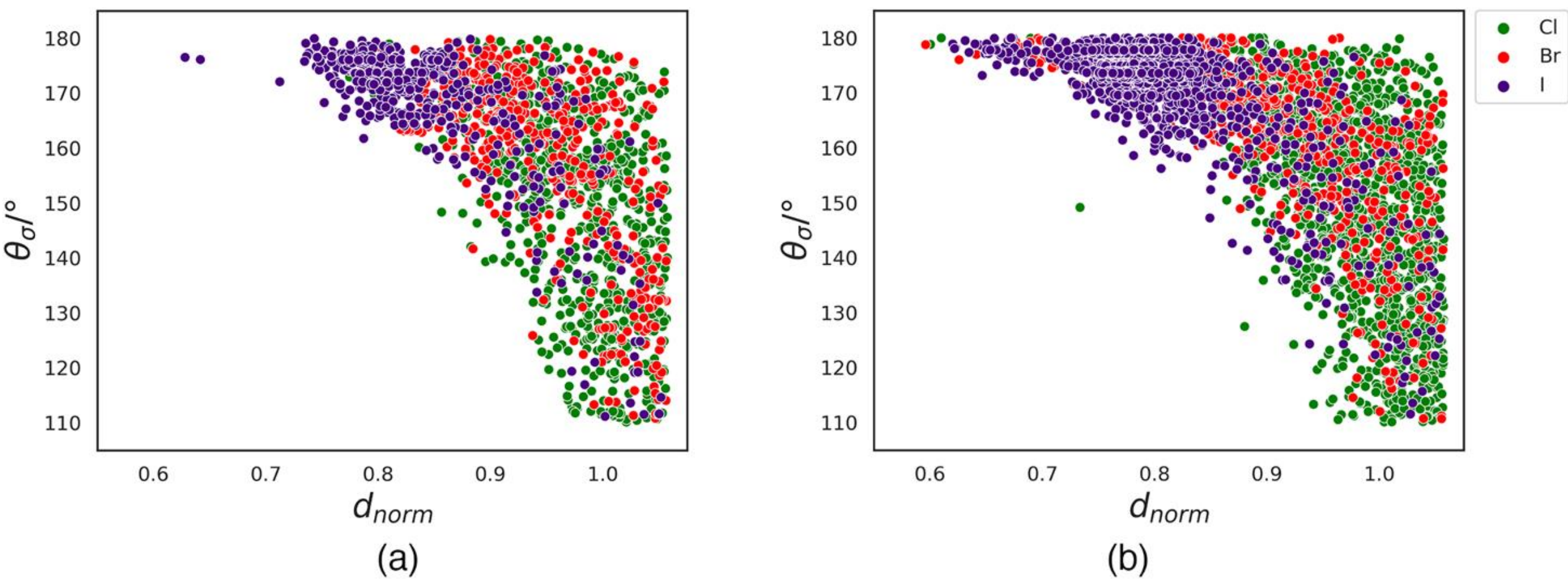

Figure S15. Distribution of geometries for halogen bonds involving N_{sp^2} acceptor groups (a) in 5-membered rings and (b) in 6-membered rings. Normalised X···N distance (d_{norm}) vs. E−X···N σ-hole angle, θ_σ. Data points are colour-coded by halogen-bond donor atom.

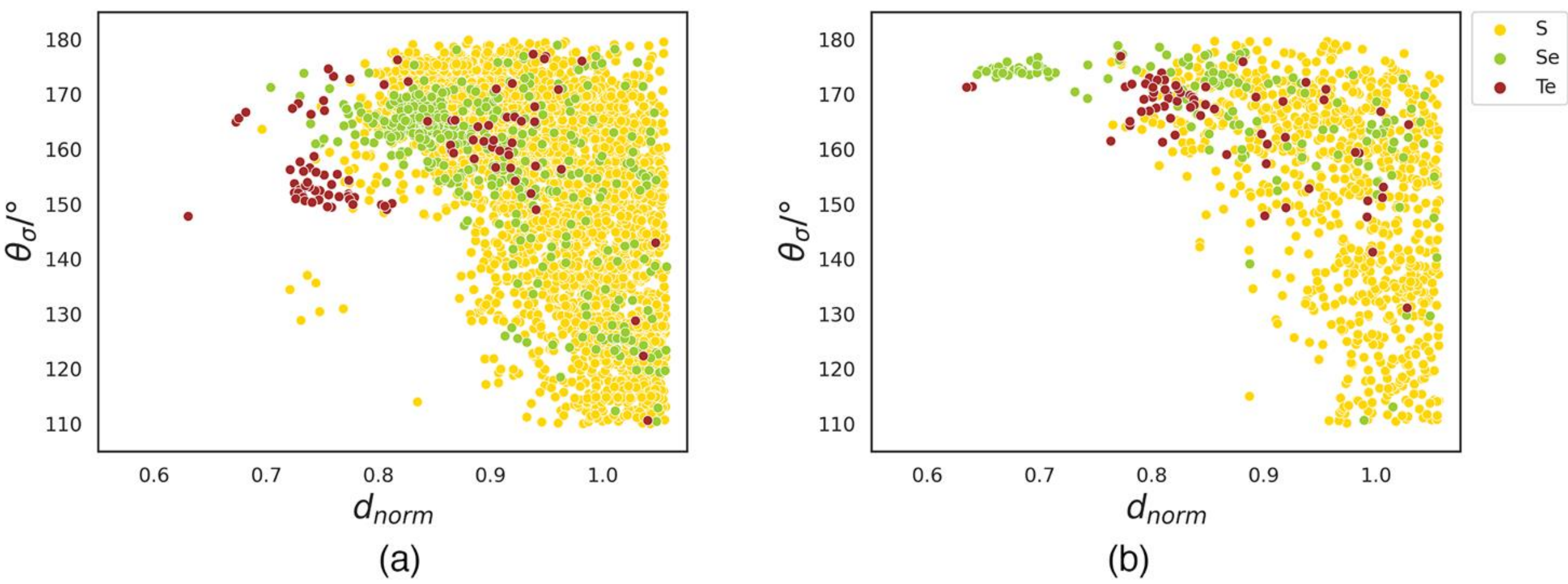

Figure S16. Distribution of geometries for chalcogen bonds involving N_{sp^2} acceptor groups (a) in 5-membered rings and (b) in 6-membered rings. Normalised X···N distance (d_{norm}) vs. E−X···N σ-hole angle, θ_σ. Data points are colour-coded by chalcogen-bond donor atom.

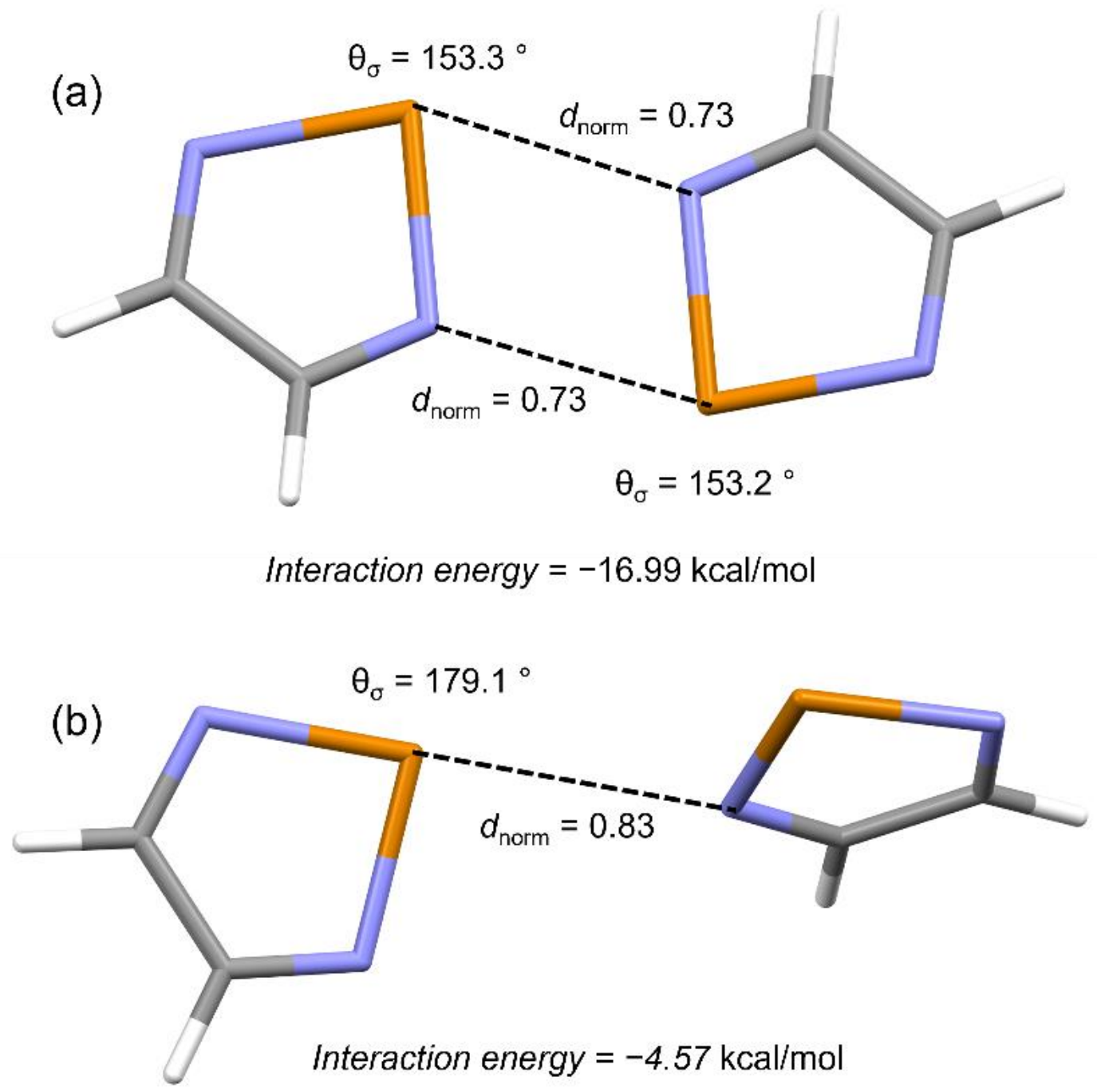

Figure S17. Geometry optimisation (DFT – M06-2X-D3(0)/def2-TZVPD level) and intermolecular interaction energy calculations (PNO-LCCSD(T*)-F12b/cc-pVDZ-F12) for the dimer of 1,2,5-telluradiazole, optimised for interaction through (a) two N−Te··· N_{sp^2} chalcogen bonds and (b) one N−Te··· N_{sp^2} chalcogen bond. It is apparent that any small loss of interaction energy that may arise from deviation from linearity of θ_σ angle(s) required to accommodate simultaneously two chalcogen bonds (e.g. see SAPT calculations for in Figure S35) is more than compensated by the additional stabilisation afforded by having the two chalcogen bonds.

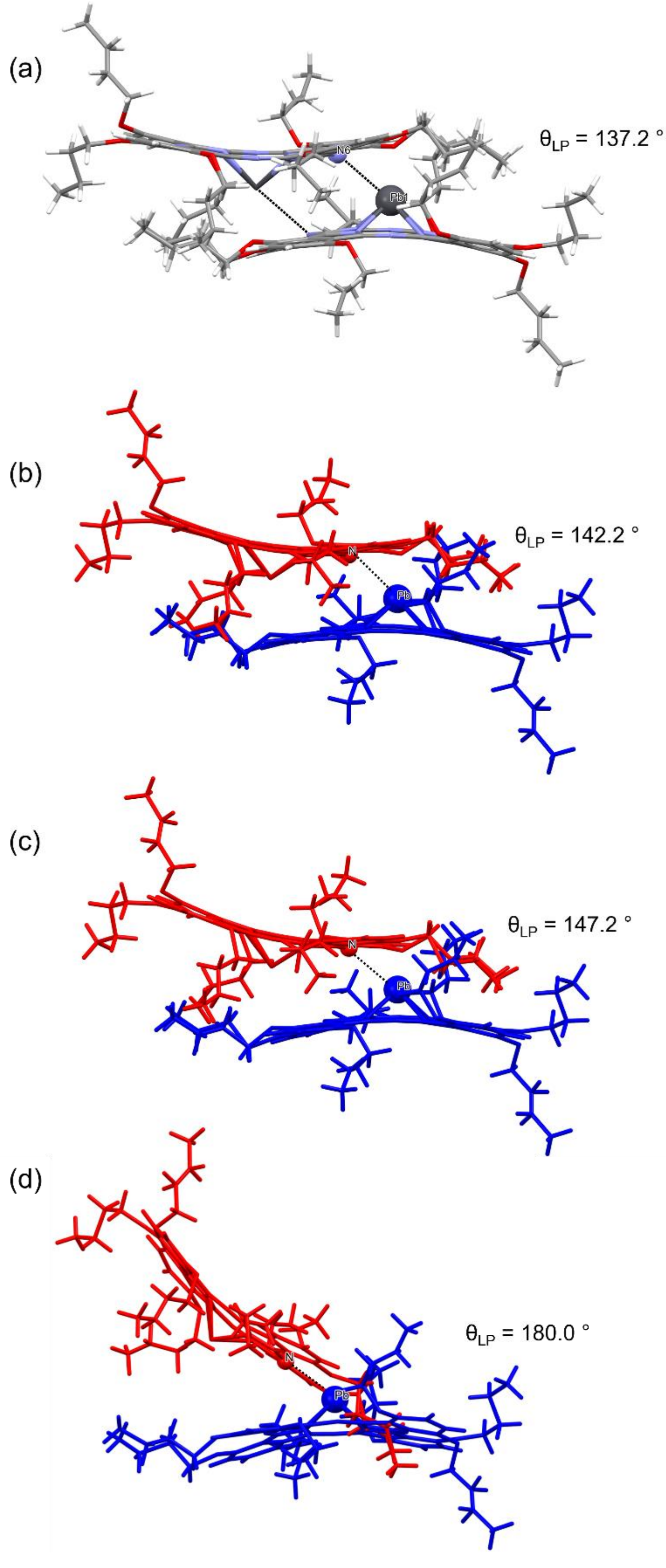

Figure S18. (a) Crystal structure of (1,4,8,11,15,18,22,25-octabutoxyphthalocyanine)-lead(II) (CSD refcode: AJOGOW)[S34] showing face-to-face π-stacking arrangement of the hemidirected Pb(II) complexes, which incorporate two N−Pb··· N_{sp^2} tetrel bonds in which the θ_{LP} angle deviates substantially from linearity (137.2°). In panels (b)-(d) the upper molecule of the pair (colour in red) has been rotated about the tetrel-bond-acceptor N_{sp^2} atom to increase the θ_{LP} angle, while maintaining the Pb··· N_{sp^2} separation (2.980 Å, d_{norm} = 0.70) and σ-hole angle (θ_σ = 147.1°) for that interaction. These panels illustrate the increasing steric clash, seen at its most extreme in panel (d) where θ_{LP} = 180°. This sequence of images provides the structural evidence to demonstrate why tetrel bonds formed by hemidirected Pb(II) complexes often have θ_{LP} angle that deviate substantially from linearity to avoid the steric clash and to maximise π-stacking and/or dispersion interactions involving the main coordination face of the complex. Colour scheme in panel (a): C, light grey; H, white; N, blue; O, red; Pb, dark grey.

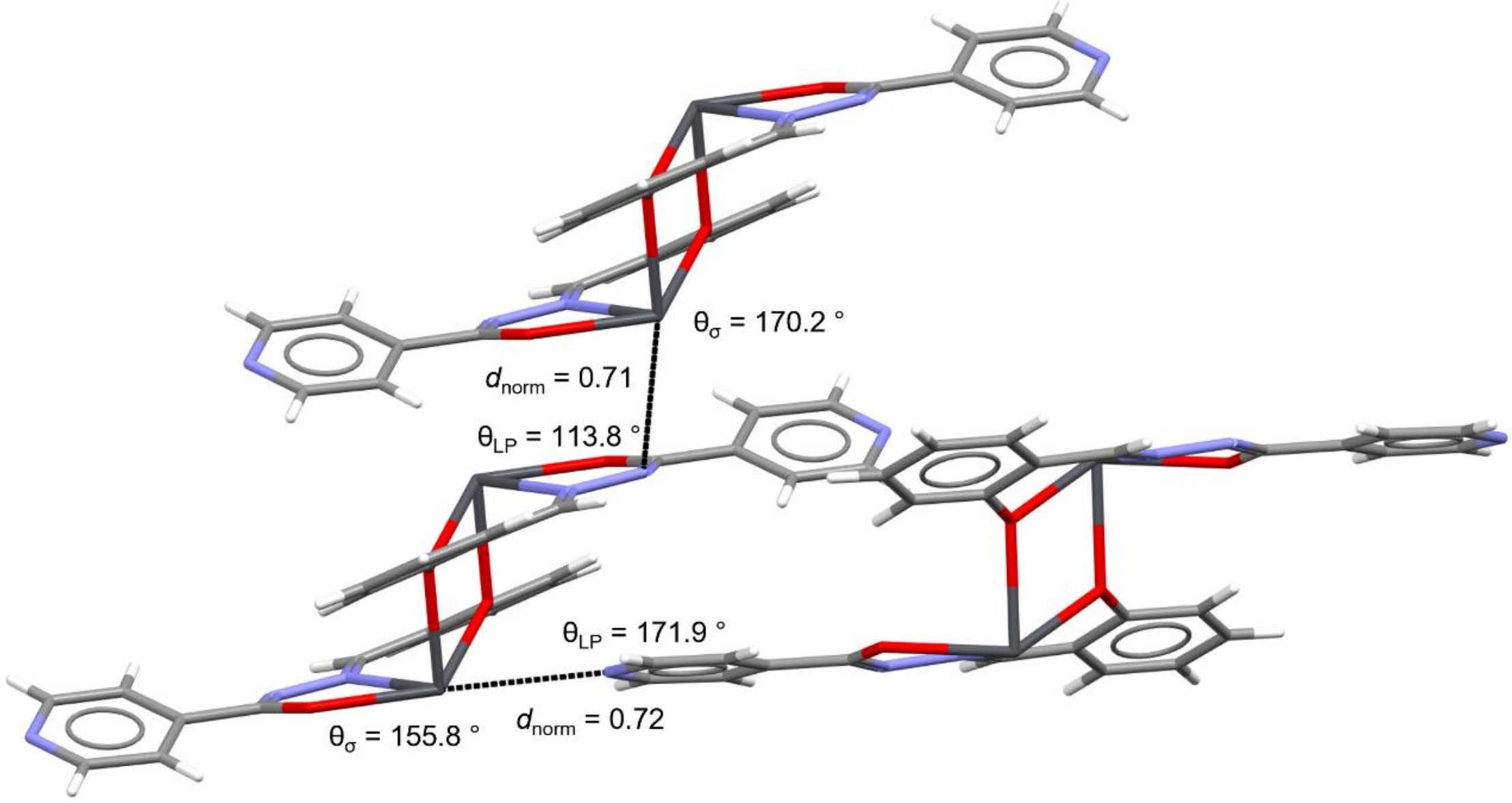

Figure S19. Crystal structure of bis(μ2-N-isonicotinamidosalicylaldiminato)-dilead((II) ethanol solvate (CSD refcode: CIBSOW)[S35] showing O−Pb··· N_{sp^2} tetrel bonds that can accommodate near-linear θ_σ or θ_{LP} angles in the absence of a large coordination face for the hemidirected Pb(II) environment or through the presence of a pendant group (here pyridyl) that can provide access to the Pb(II) centre. The ethanol solvent molecule is not shown.

S4.3 σ-Hole Interactions with N_{sp} Acceptor Groups

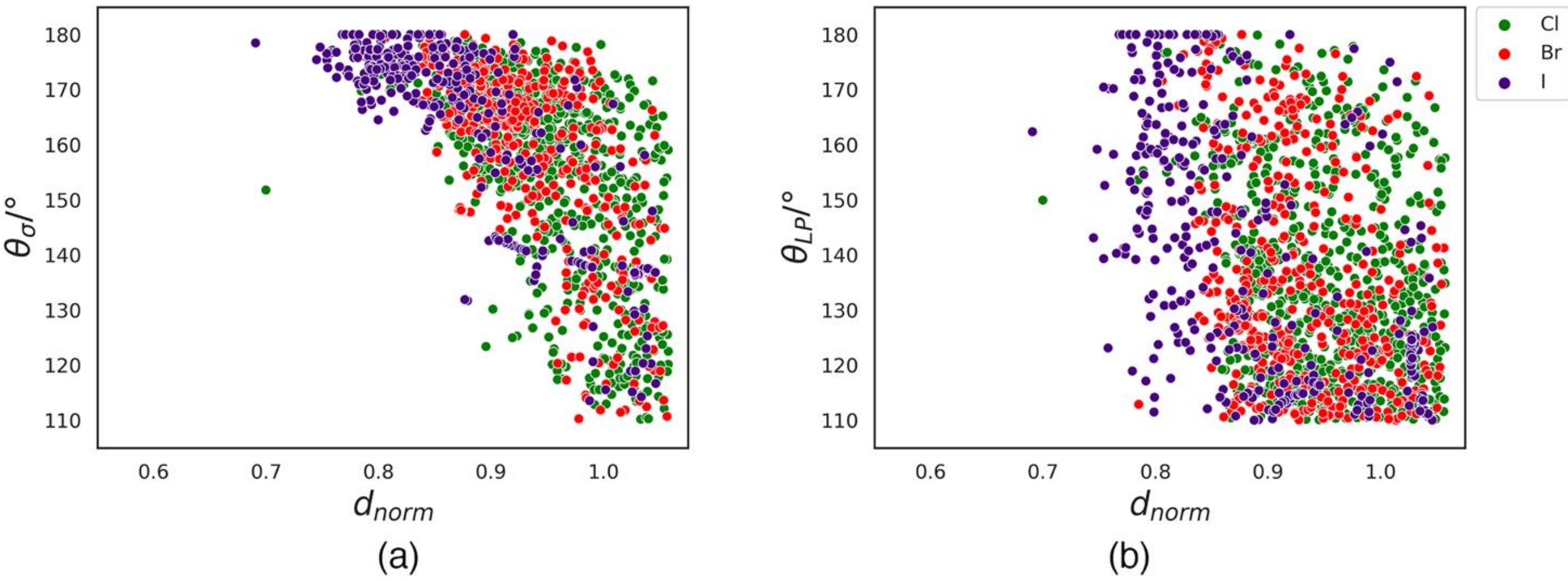

Figure S20. Distribution of geometries for halogen bonds involving N_{sp} acceptor groups. (a) Normalised X···N distance (d_{norm}) vs. E−X···N σ-hole angle, $θ_σ$. (b) Normalised X···N distance(d_{norm}) vs. N_{sp} lone-pair angle, $θ_{LP}$. Data points are colour-coded by halogen-bond donor atom.

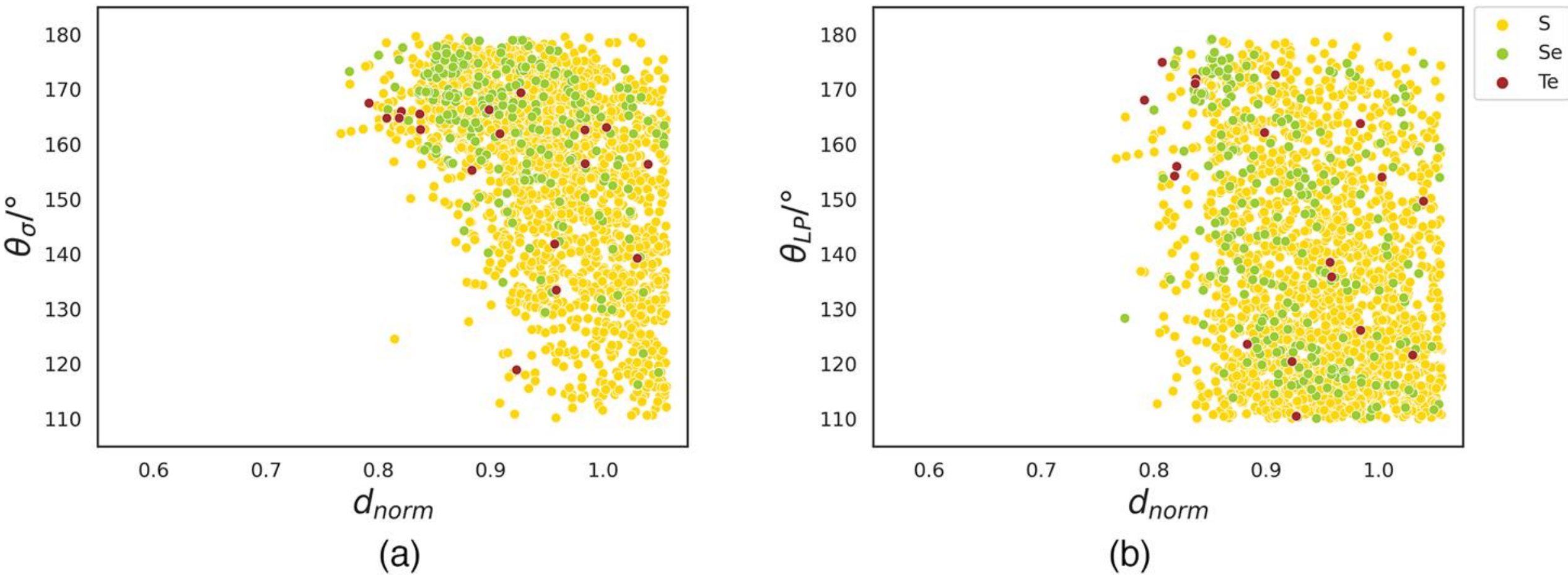

Figure S21. Distribution of geometries for chalcogen bonds involving N_{sp} acceptor groups. (a) Normalised Ch···N distance (d_{norm}) vs. E−Ch···N σ-hole angle, $θ_σ$. (b) Normalised Ch···N distance (d_{norm}) vs. N_{sp} lone-pair angle, $θ_{LP}$. Data points are colour-coded by chalcogen-bond donor atom.

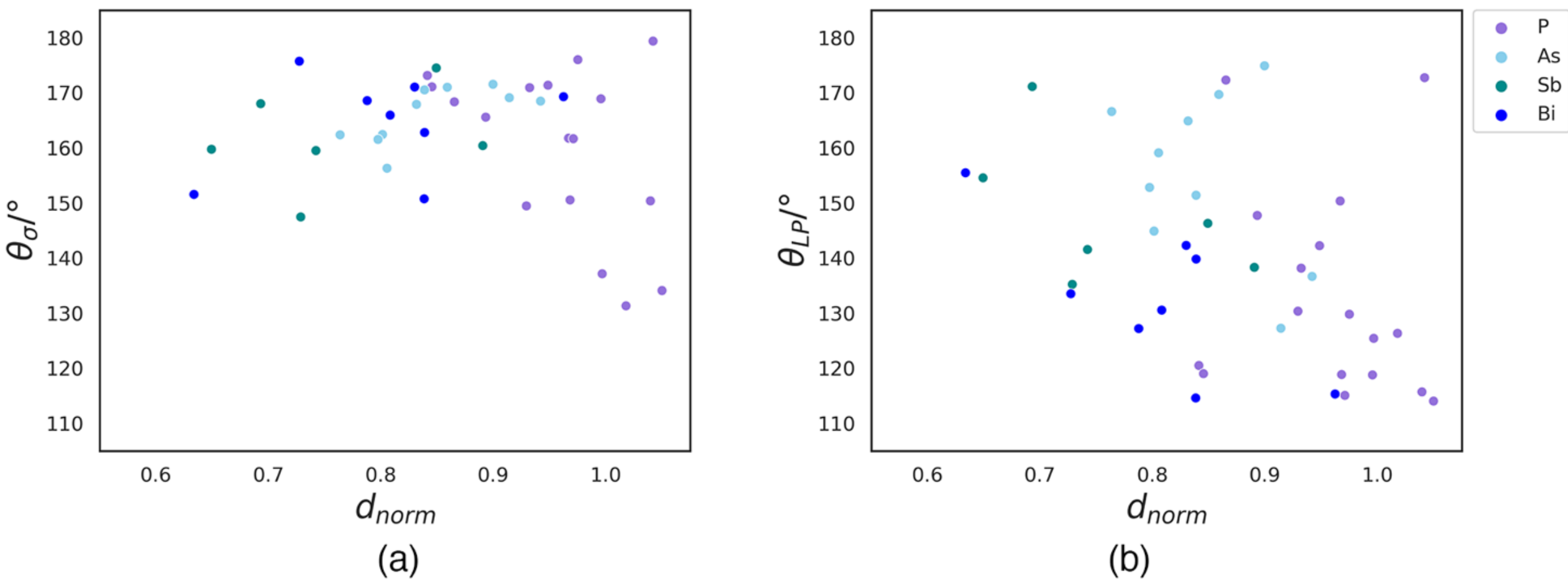

Figure S22. Distribution of geometries for pnictogen bonds involving N_{sp} acceptor groups. (a) Normalised Pn···N distance (d_{norm}) vs. E−Pn···N σ-hole angle, $θ_σ$. (b) Normalised Pn···N distance (d_{norm}) vs. N_{sp} lone-pair angle, $θ_{LP}$. Data points are colour-coded by pnictogen-bond donor atom.

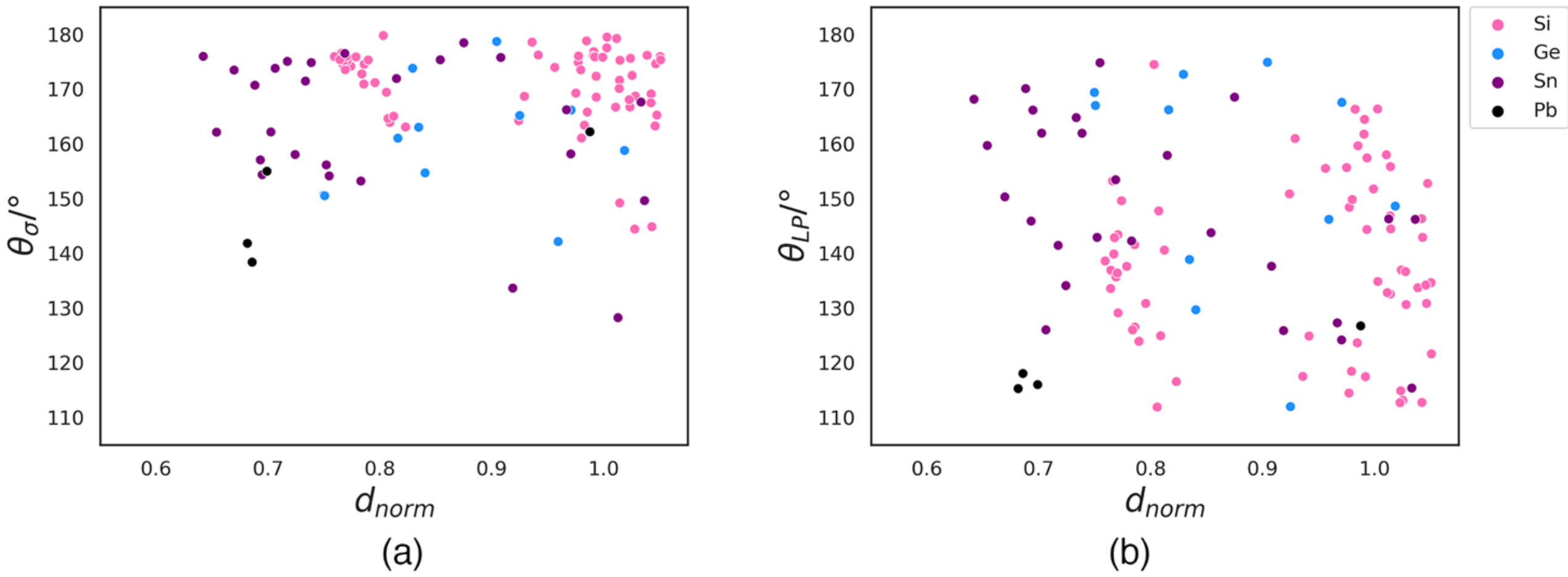

Figure S23. Distribution of geometries for tetrel bonds involving N_{sp} acceptor groups. (a) Normalised Tt···N distance (d_{norm}) vs. E−Tt···N σ-hole angle, θ_{σ}. (b) Normalised Tt···N distance (d_{norm}) vs. N_{sp} lone-pair angle, θ_{LP}. Data points are colour-coded by tetrel-bond donor atom.

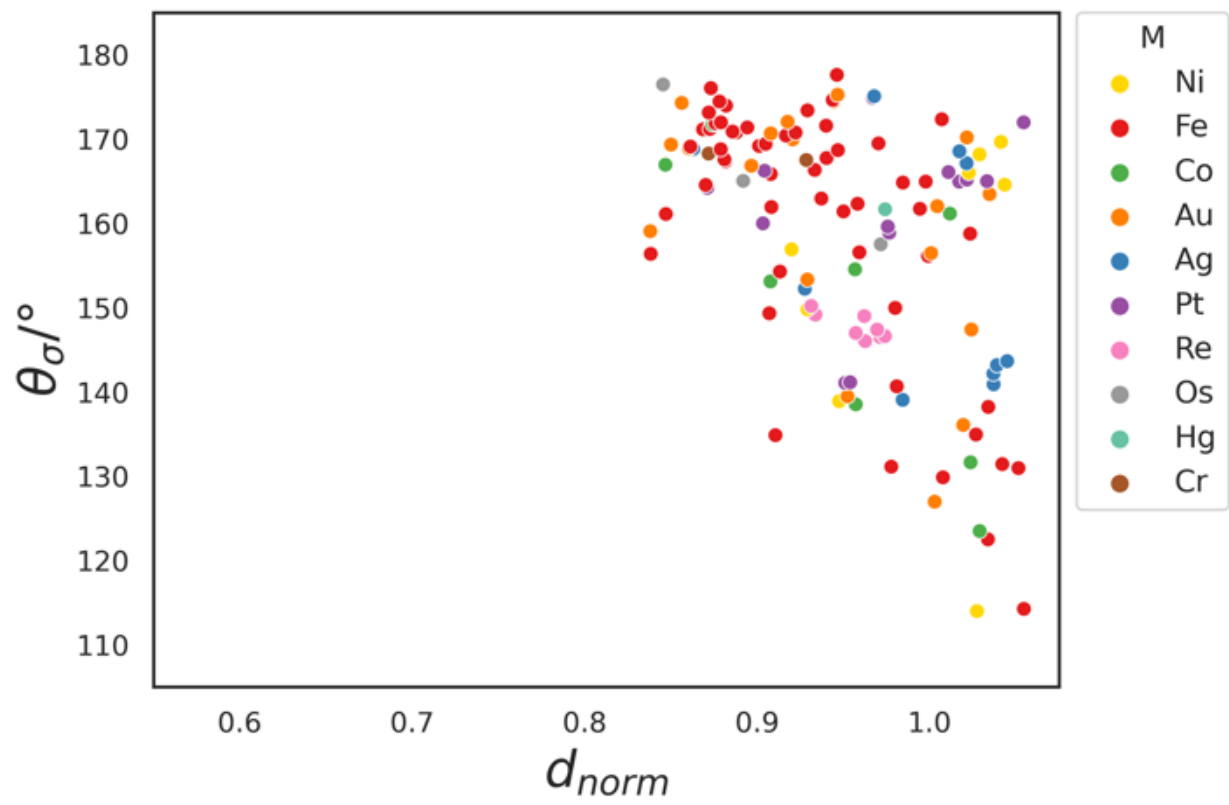

Figure S24. Distribution of normalised Ch_{σ}···N distance (d_{norm}) vs. E−Ch_{σ}···N σ-hole angle (θ_{σ}) for chalcogen bonds to N≡C−M moieties (predominately involving cyanometallates). Data points colour coded by metal (M) species.

S4.4 σ-Hole Donor Substituent Effects

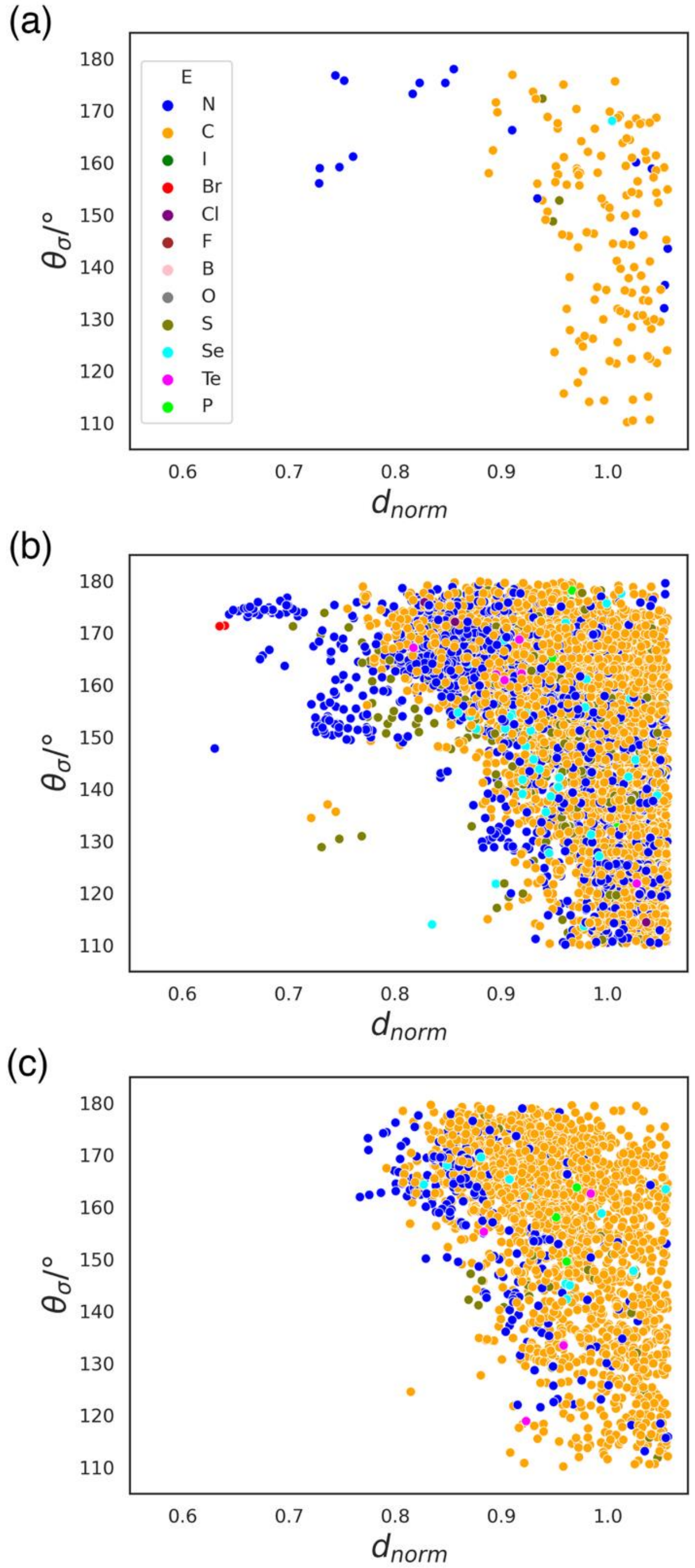

Figure S25. Distribution of geometries for chalcogen bonds involving (a) N_{sp^3}, (b) N_{sp^2} and (c) N_{sp} acceptor groups represented as normalised $Ch_{\sigma}\cdots N$ distance (d_{norm}) vs. $E-Ch_{\sigma}\cdots N$ σ-hole angle, θ_{σ}. Data points are colour-coded by substituent (E) on the chalcogen-bond donor atom.

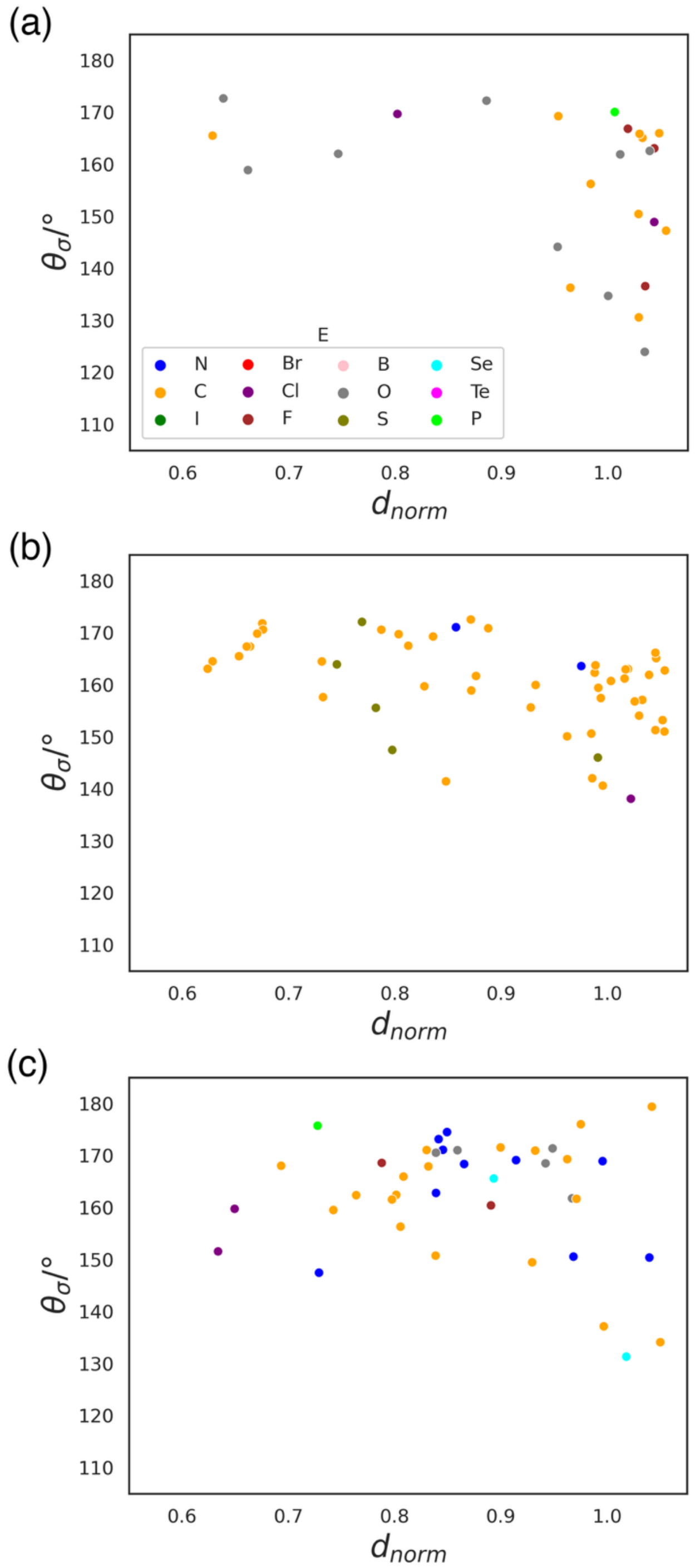

Figure S26. Distribution of geometries for pnictogen bonds involving (a) N_{sp^3}, (b) N_{sp^2} and (c) N_{sp} acceptor groups represented as normalised Pn_σ···N distance (d_{norm}) vs. E−Pn_σ···N σ-hole angle, θ_σ. Data points are colour-coded by substituent (E) on the pnictogen-bond donor atom.

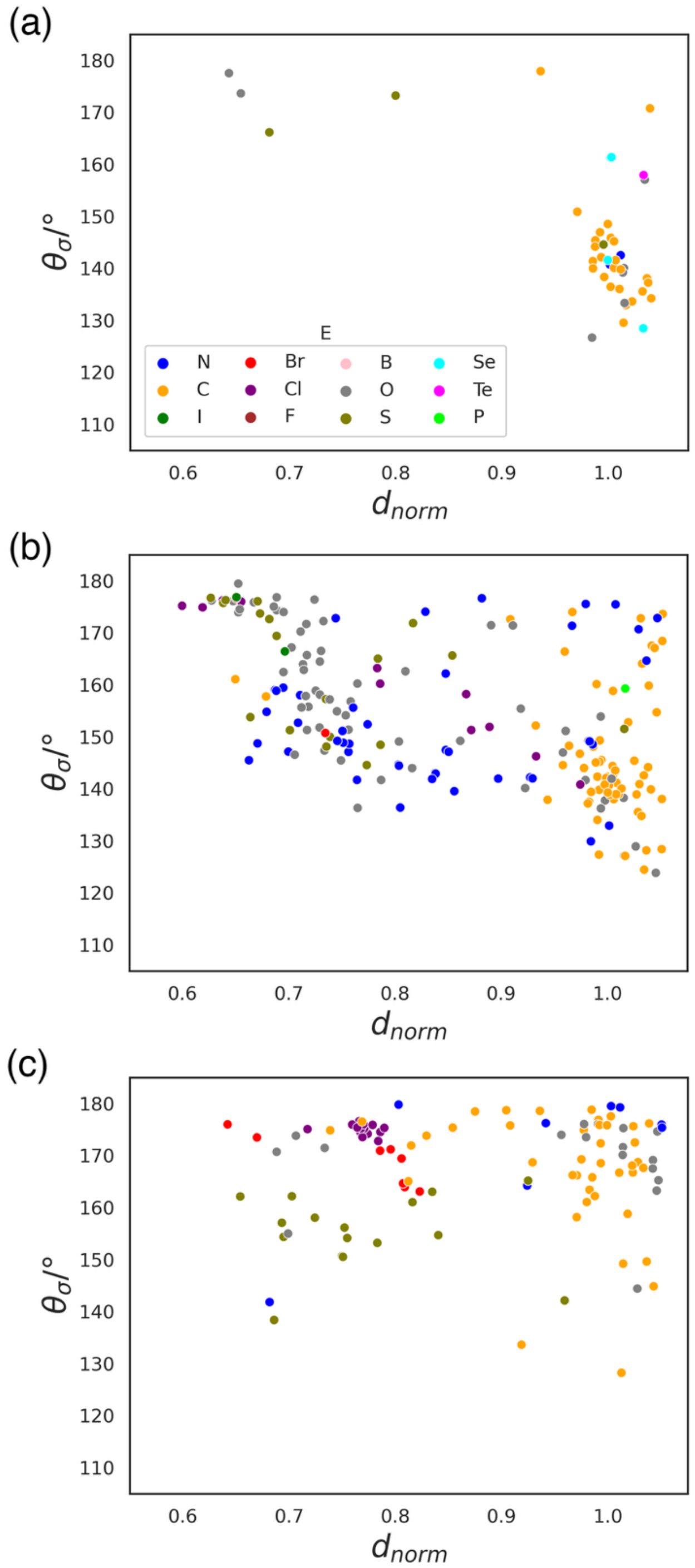

Figure S27. Distribution of geometries for tetrel bonds involving (a) N_{sp}^3, (b) N_{sp}^2 and (c) N_{sp} acceptor groups represented as normalised $Tt_\sigma\cdots N$ distance (d_{norm}) vs. $E{-}Tt_\sigma\cdots N$ σ-hole angle, θ_σ. Data points are colour-coded by substituent (E) on the tetrel-bond donor atom.

S4.5 σ-Hole Interaction Energies and Geometries

Figure S28 enables comparison of trends in interactions energies, calculated for model σ-hole intermolecular complexes (see Figure 14) with trends in experimental interaction geometries, represented here as the mean values of d_{norm}, θ_σ and θ_{LP}.

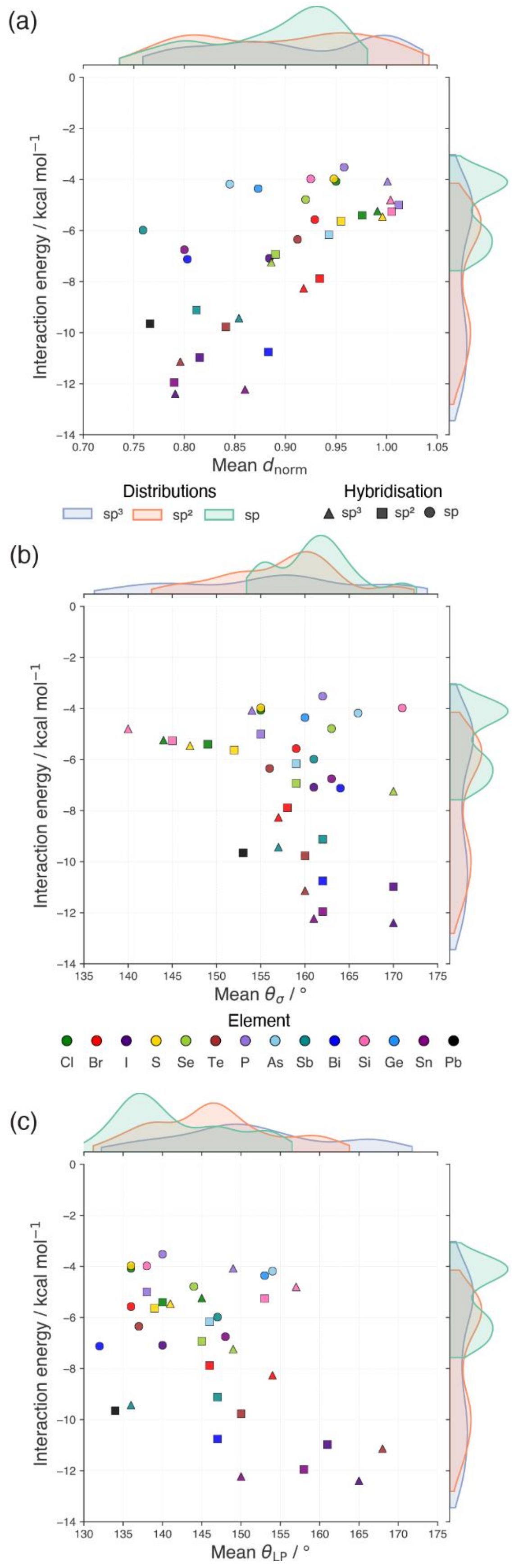

Figure S28. Distribution of σ-hole interaction energies calculated for model intermolecular complexes (see Figure 14) plotted as a function of mean geometric parameters (a) d_{norm}, (b) θ_σ and (c) θ_{LP} for analogous σ-hole interaction (from CSD crystallographic data). Smoothed histograms for energetic and geometric data are grouped by N hybridisation state.

S5. SAPT σ-Hole Angle Scans

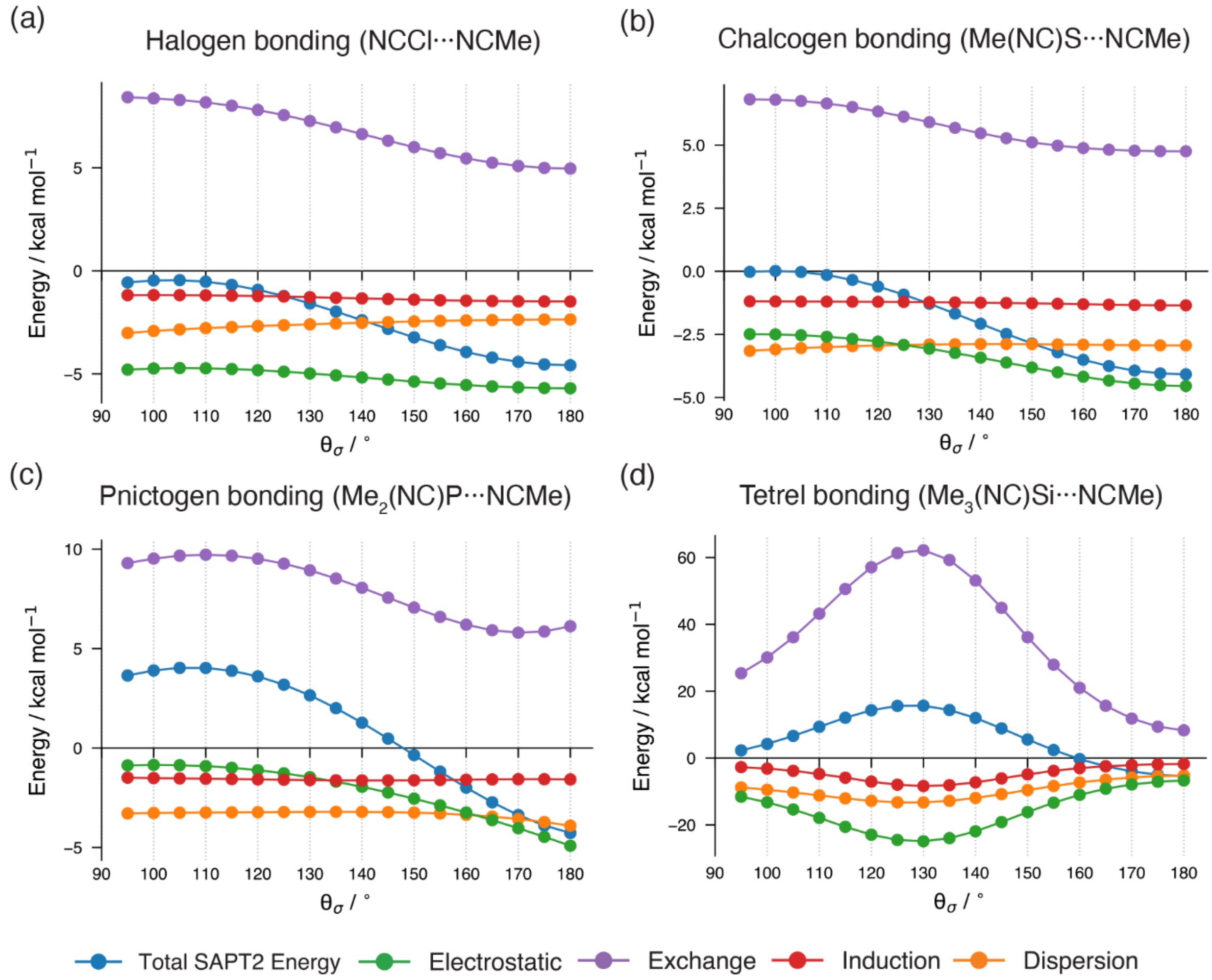

Figure S29. SAPT2/def2-TZVPD energetic components as a function of the σ-hole angle (θ_σ) for: (a) halogen bonding between ClCN and NCMe, (b) chalcogen bonding between S(CN)Me and NCMe, (c) pnictogen bonding between $P(CN)Me_2$ and NCMe, and (d) tetrel bonding between $Si(CN)Me_3$ and NCMe. The total SAPT2 interaction energy is abbreviated as "Total SAPT2 Energy".

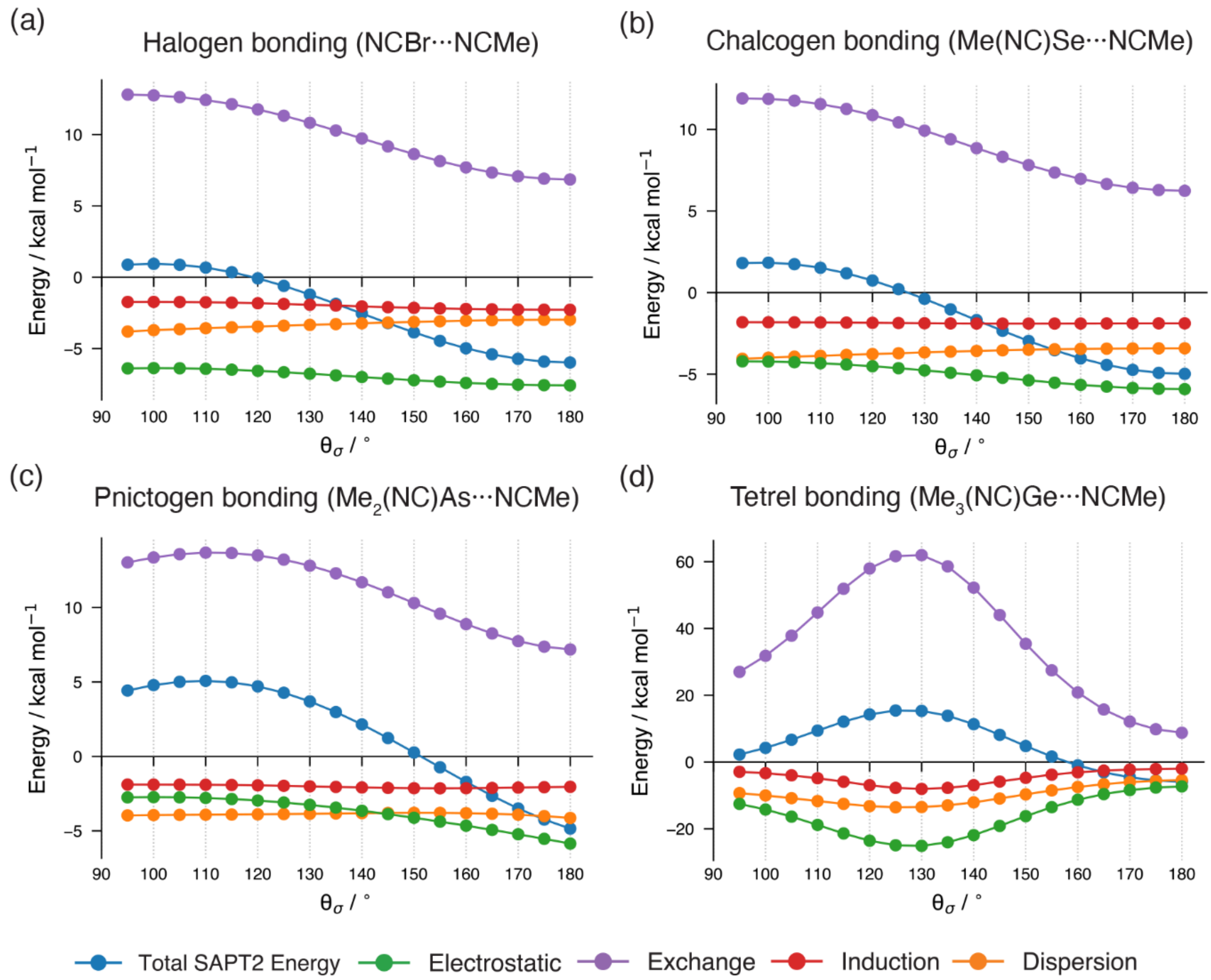

Figure S30. SAPT2/def2-TZVPD energetic components as a function of the σ-hole angle (θ_σ) for: (a) halogen bonding between BrCN and NCMe, (b) chalcogen bonding between Se(CN)Me and NCMe, (c) pnictogen bonding between $As(CN)Me_2$ and NCMe, and (d) tetrel bonding between $Ge(CN)Me_3$ and NCMe. The total SAPT2 interaction energy is abbreviated as “Total SAPT2 Energy”.

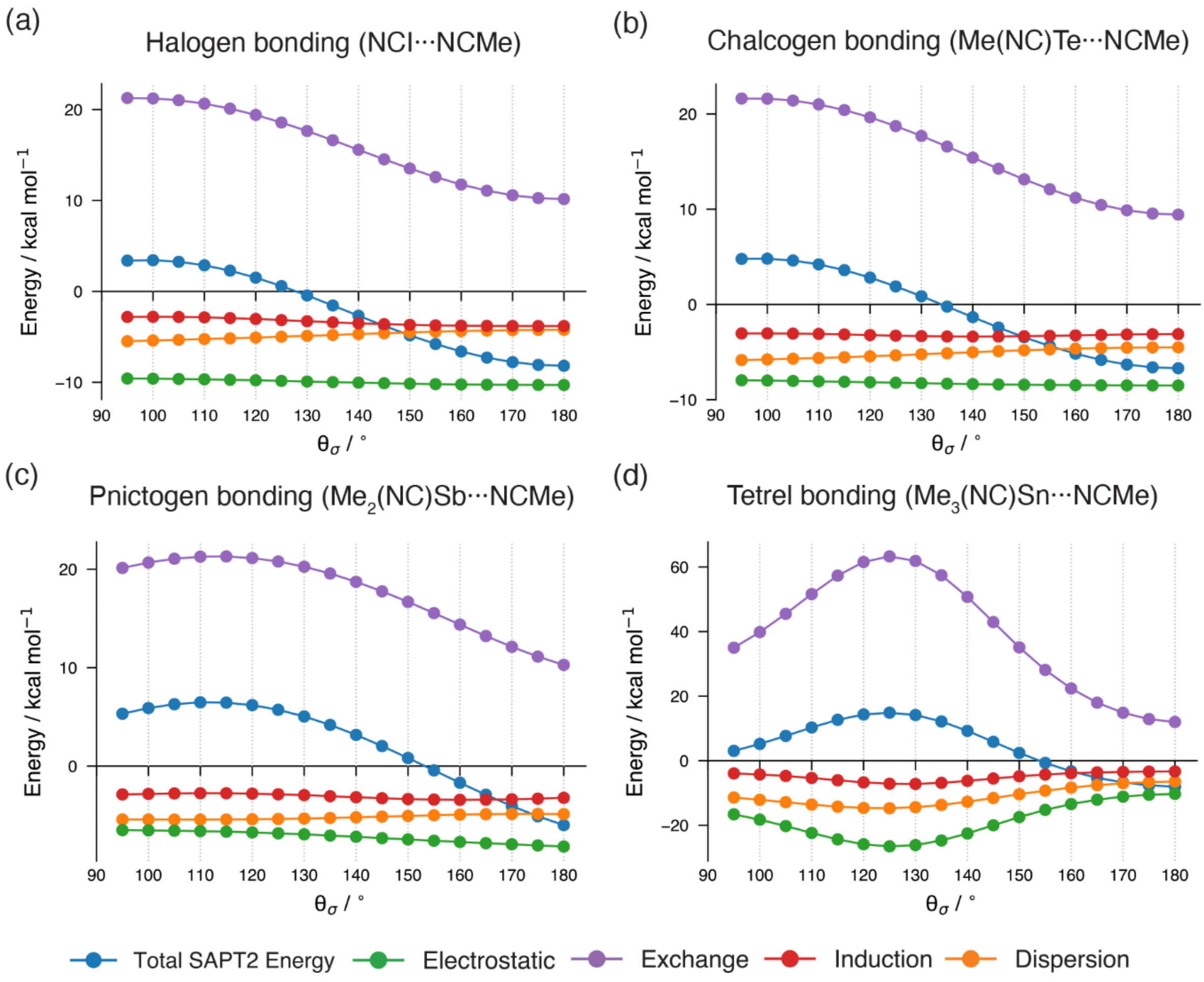

Figure S31. SAPT2/def2-TZVPD energetic components as a function of the σ-hole angle (θ_σ) for: (a) halogen bonding between ICN and NCMe, (b) chalcogen bonding between Te(CN)Me and NCMe, (c) pnictogen bonding between $Sb(CN)Me_2$ and NCMe, and (d) tetrel bonding between $Sn(CN)Me_3$ and NCMe. The total SAPT2 interaction energy is abbreviated as "Total SAPT2 Energy".

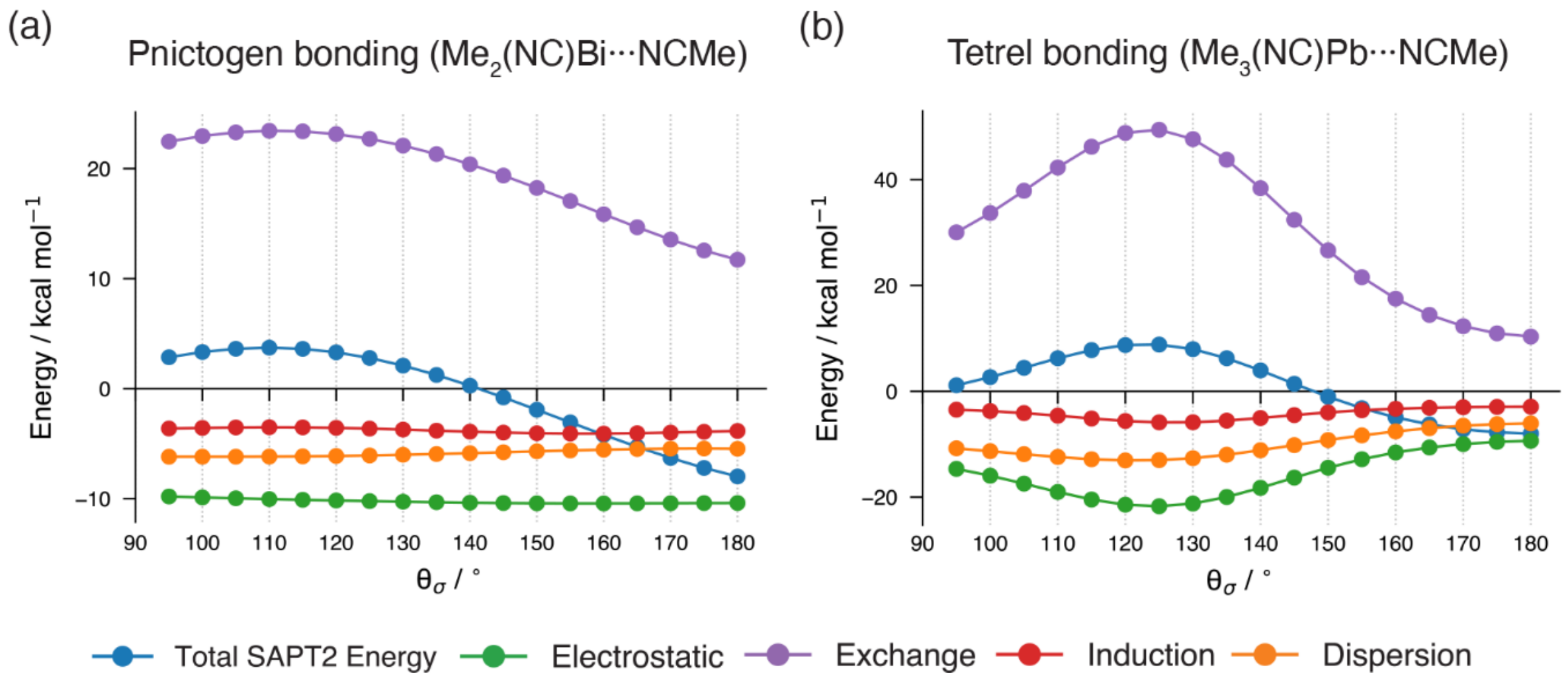

Figure S32. SAPT2/def2-TZVPD energetic components as a function of the σ-hole angle (θ_σ) for: (a) pnictogen bonding between $Bi(CN)Me_2$ and NCMe, and (b) tetrel bonding between $Pb(CN)Me_3$ and NCMe. The total SAPT2 interaction energy is abbreviated as “Total SAPT2 Energy”.

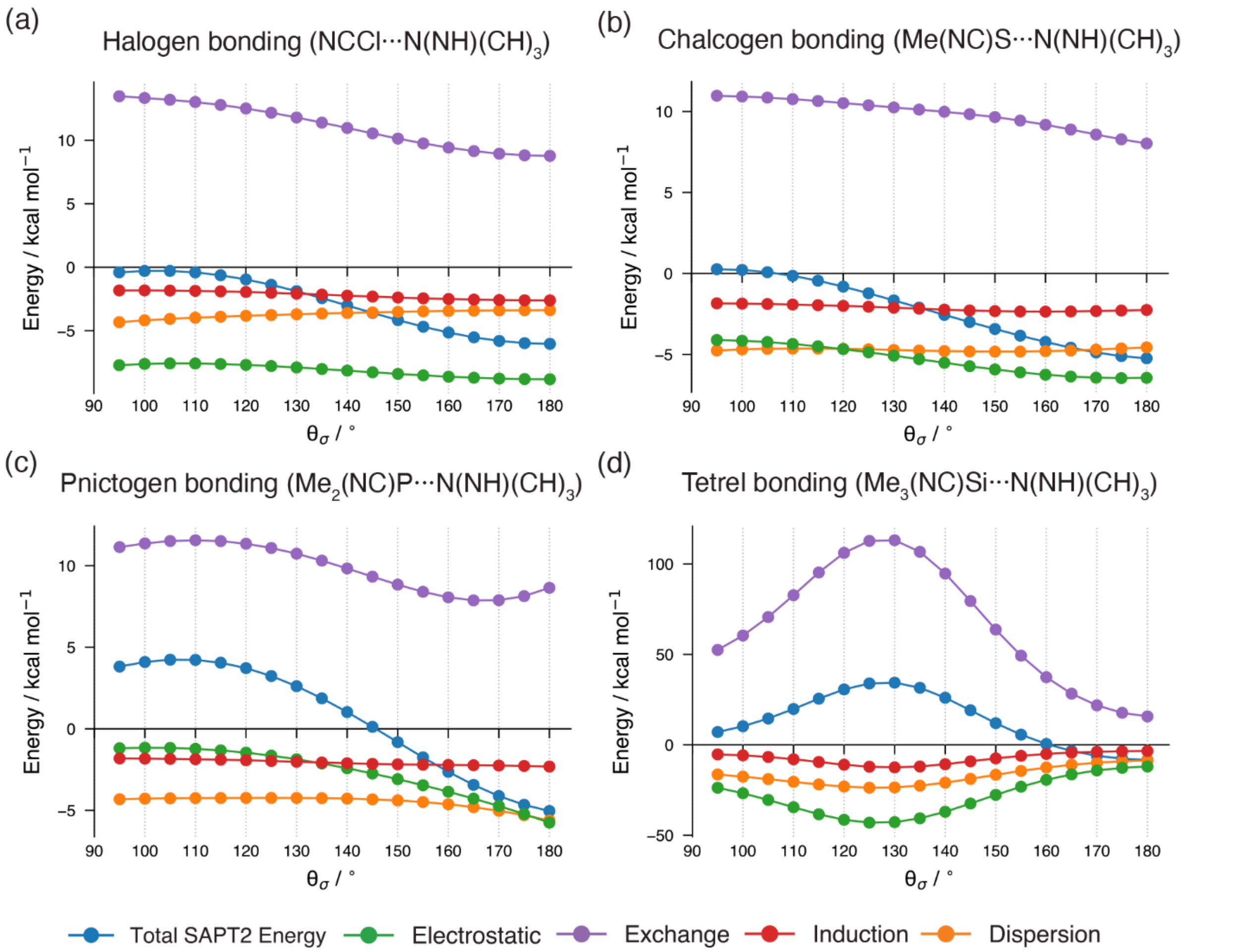

Figure S33. SAPT2/def2-TZVPD energetic components as a function of the σ-hole angle (θ_σ) for: (a) halogen bonding between ClCN and imidazole, (b) chalcogen bonding between S(CN)Me and imidazole, (c) pnictogen bonding between $P(CN)Me_2$ and imidazole, and (d) tetrel bonding between $Si(CN)Me_3$ and imidazole. The total SAPT2 interaction energy is abbreviated as "Total SAPT2 Energy".

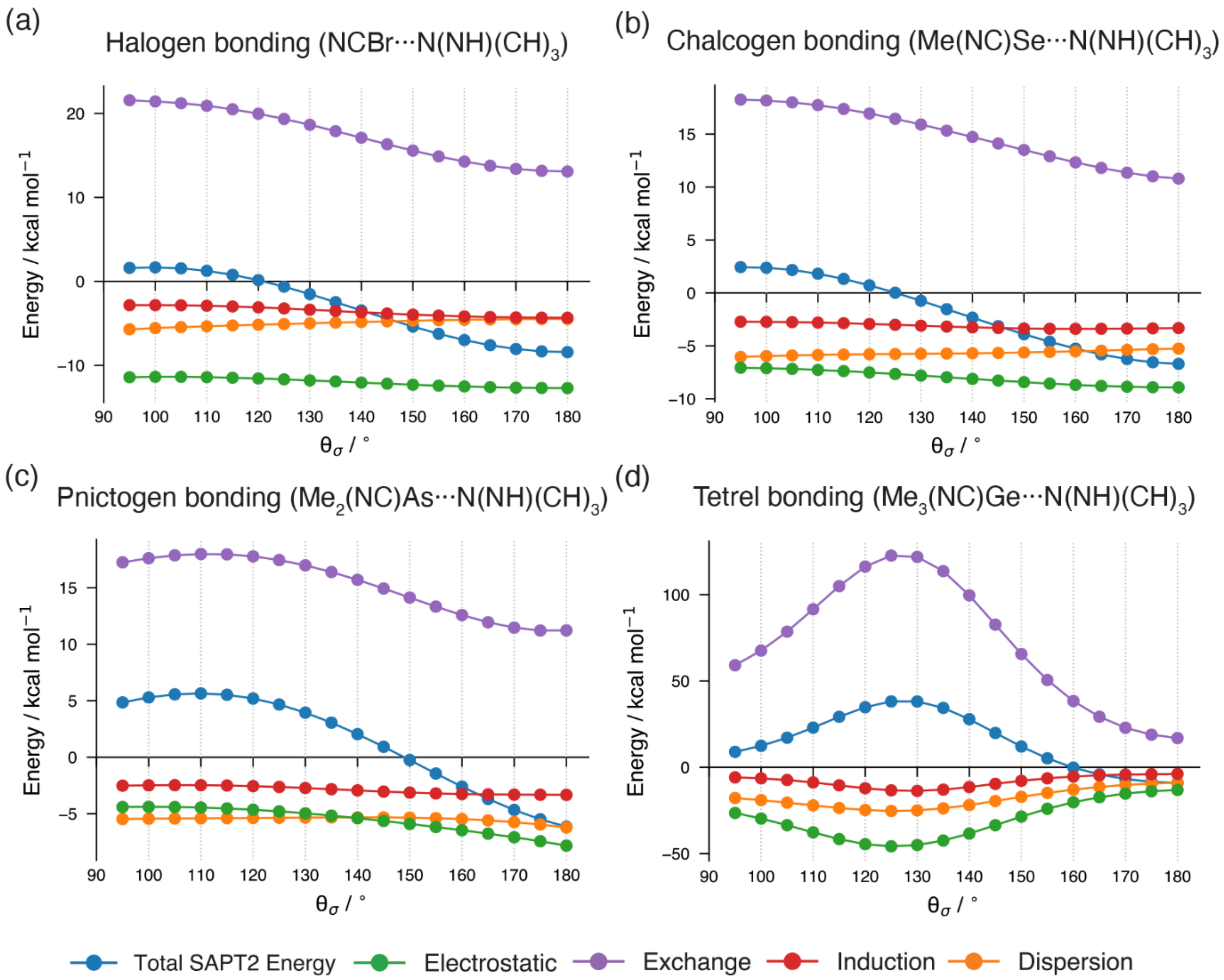

Figure S34. SAPT2/def2-TZVPD energetic components as a function of the σ-hole angle (θ_σ) for: (a) halogen bonding between BrCN and imidazole, (b) chalcogen bonding between Se(CN)Me and imidazole, (c) pnictogen bonding between As(CN)Me_2 and imidazole, and (d) tetrel bonding between Ge(CN)Me_3 and imidazole. The total SAPT2 interaction energy is abbreviated as “Total SAPT2 Energy”.

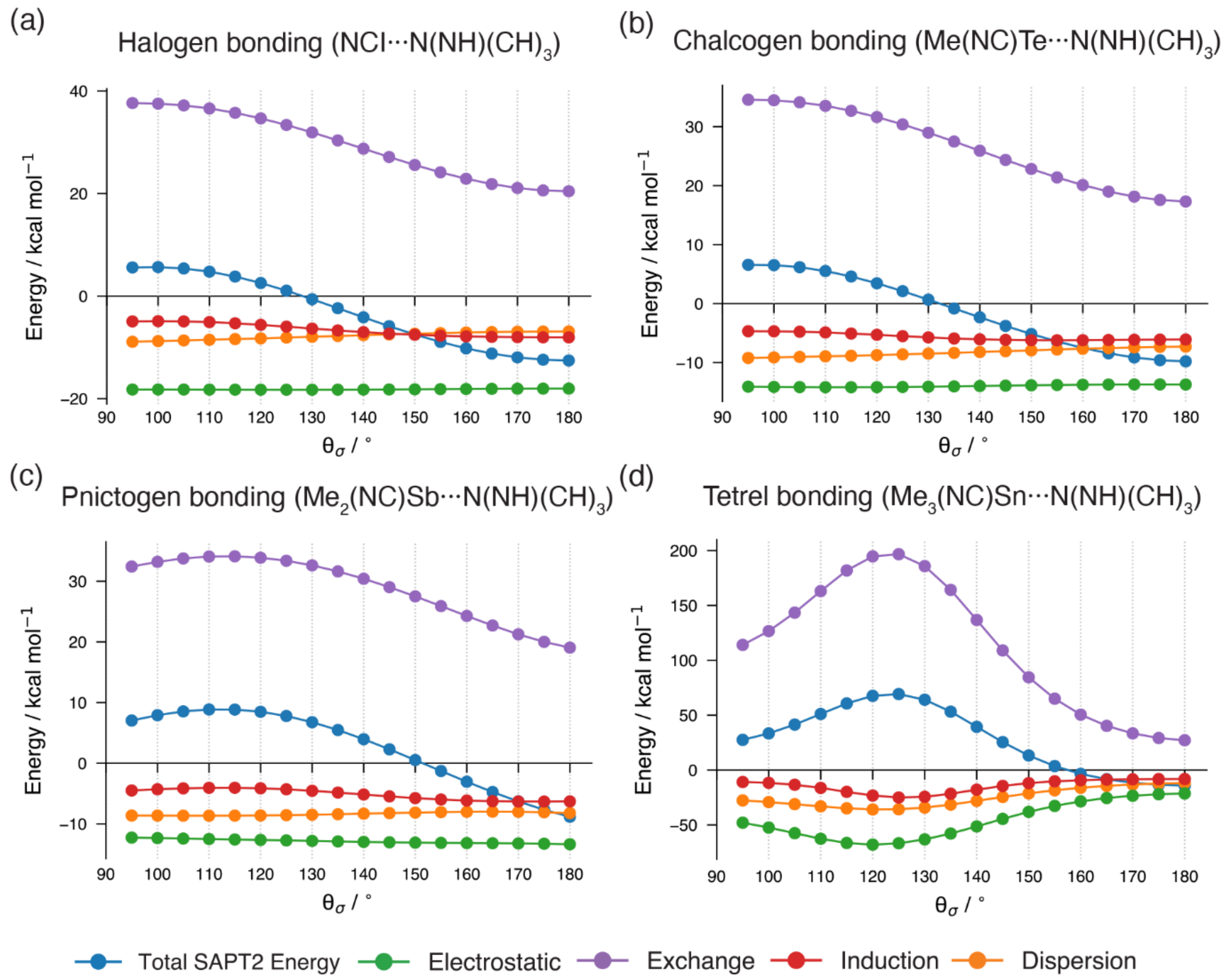

Figure S35. SAPT2/def2-TZVPD energetic components as a function of the σ-hole angle (θ_σ) for: (a) halogen bonding between ICN and imidazole, (b) chalcogen bonding between Te(CN)Me and imidazole, (c) pnictogen bonding between $Sb(CN)Me_2$ and imidazole, and (d) tetrel bonding between $Sn(CN)Me_3$ and imidazole. The total SAPT2 interaction energy is abbreviated as "Total SAPT2 Energy".

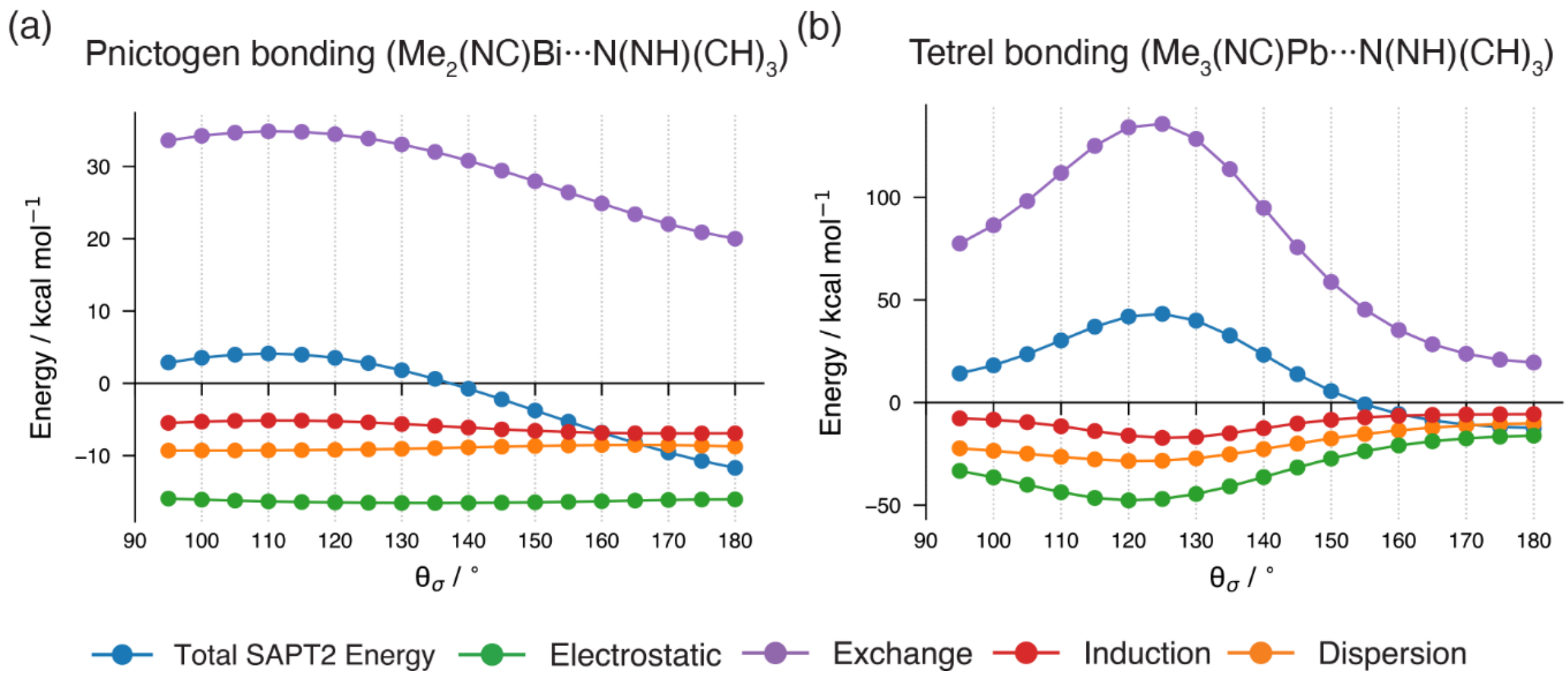

Figure S36. SAPT2/def2-TZVPD energetic components as a function of the σ-hole angle (θ_σ) for: (a) pnictogen bonding between $Bi(CN)Me_2$ and imidazole, and (b) tetrel bonding between $Pb(CN)Me_3$ and imidazole. The total SAPT2 interaction energy is abbreviated as "Total SAPT2 Energy".

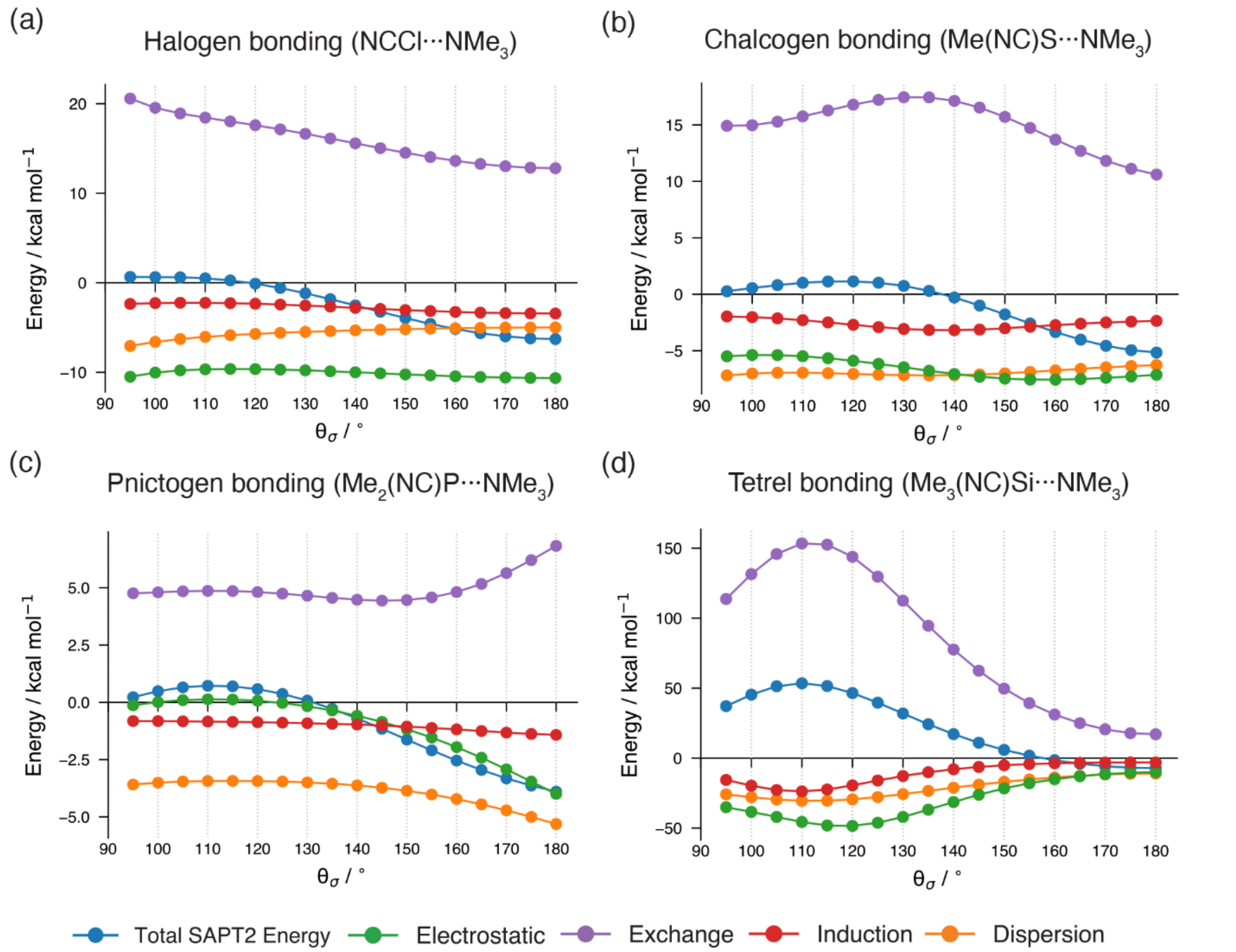

Figure S37. SAPT2/def2-TZVPD energetic components as a function of the σ-hole angle (θ_σ) for: (a) halogen bonding between ClCN and NMe_3, (b) chalcogen bonding between S(CN)Me and NMe_3, (c) pnictogen bonding between $P(CN)Me_2$ and NMe_3, and (d) tetrel bonding between $Si(CN)Me_3$ and NMe_3. The total SAPT2 interaction energy is abbreviated as “Total SAPT2 Energy”.

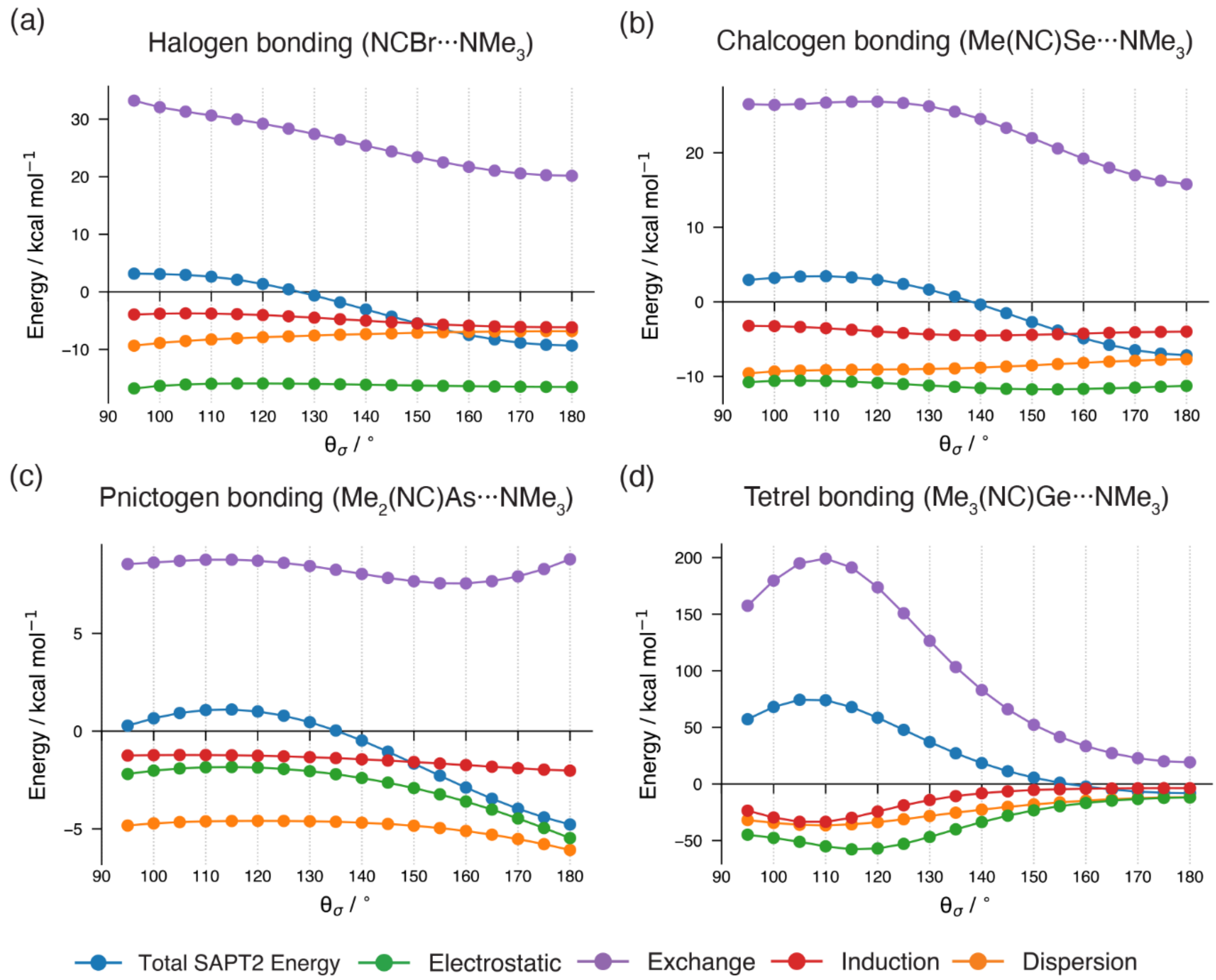

Figure S38. SAPT2/def2-TZVPD energetic components as a function of the σ-hole angle (θ_σ) for: (a) halogen bonding between BrCN and NMe_3, (b) chalcogen bonding between Se(CN)Me and NMe_3, (c) pnictogen bonding between $As(CN)Me_2$ and NMe_3, and (d) tetrel bonding between $Ge(CN)Me_3$ and NMe_3. The total SAPT2 interaction energy is abbreviated as “Total SAPT2 Energy”.

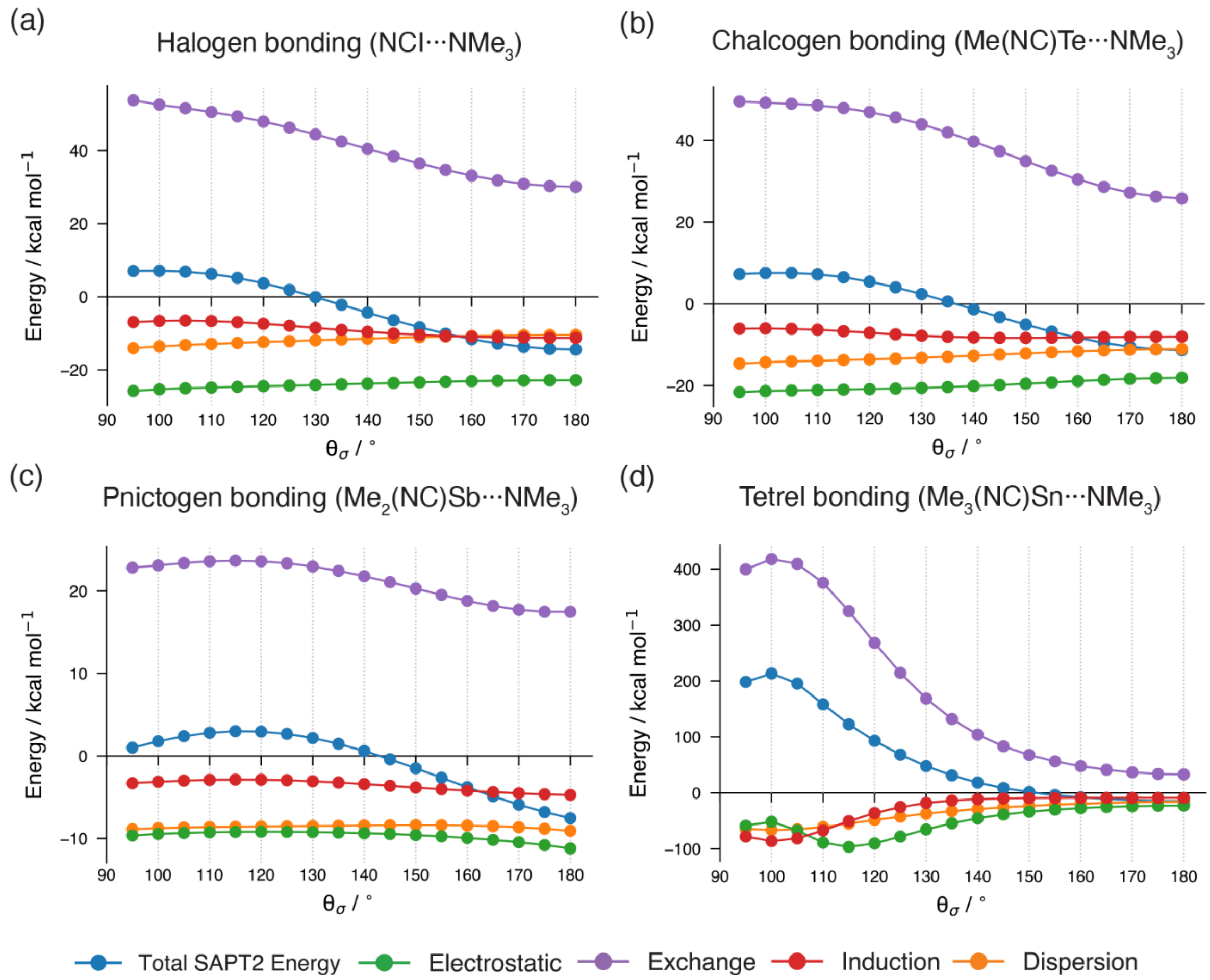

Figure S39. SAPT2/def2-TZVPD energetic components as a function of the σ-hole angle (θ_σ) for: (a) halogen bonding between ICN and NMe_3, (b) chalcogen bonding between Te(CN)Me and NMe_3, (c) pnictogen bonding between $Sb(CN)Me_2$ and NMe_3, and (d) tetrel bonding between $Sn(CN)Me_3$ and NMe_3. The total SAPT2 interaction energy is abbreviated as “Total SAPT2 Energy”.

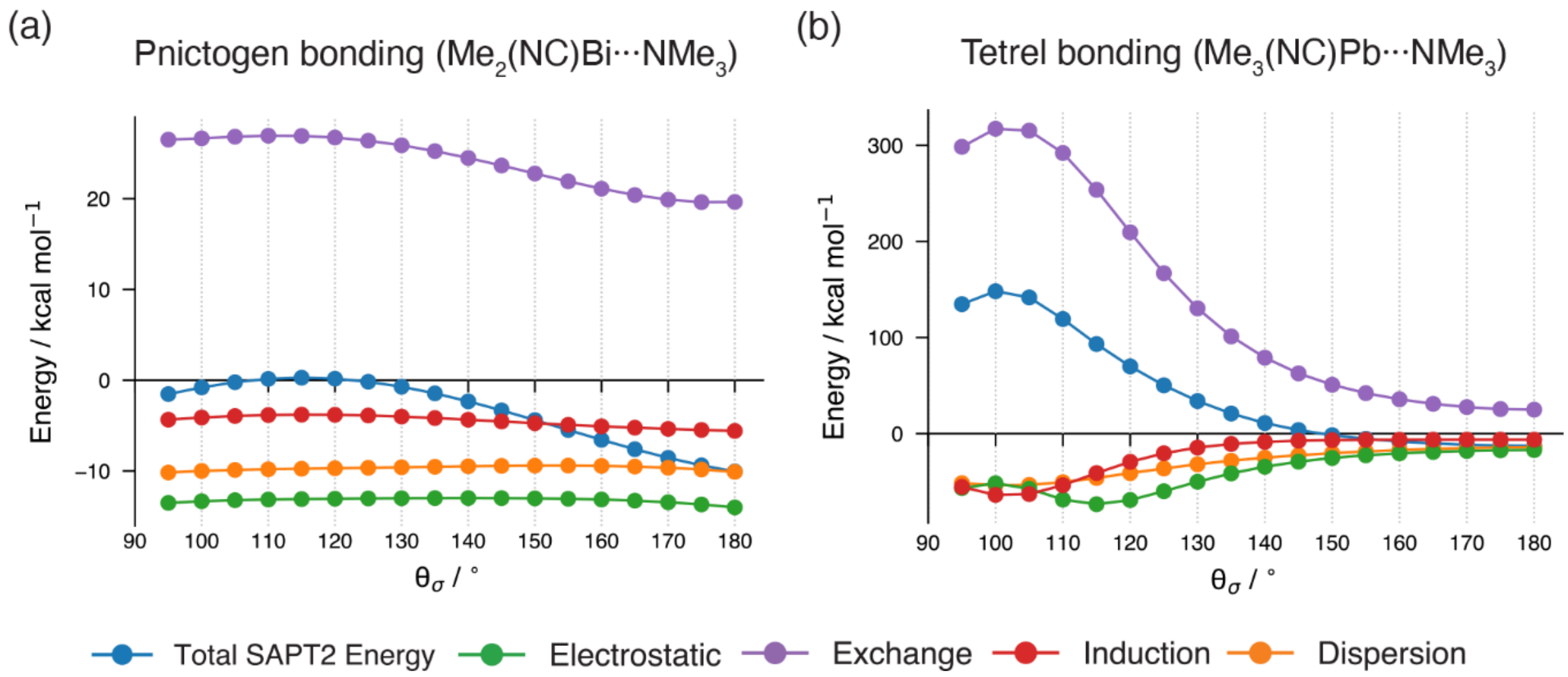

Figure S40. SAPT2/def2-TZVPD energetic components as a function of the σ-hole angle (θ_σ) for: (a) pnictogen bonding between Bi(CN)Me_2 and NMe_3, and (b) tetrel bonding between Pb(CN)Me_3 and NMe_3. The total SAPT2 interaction energy is abbreviated as "Total SAPT2 Energy".

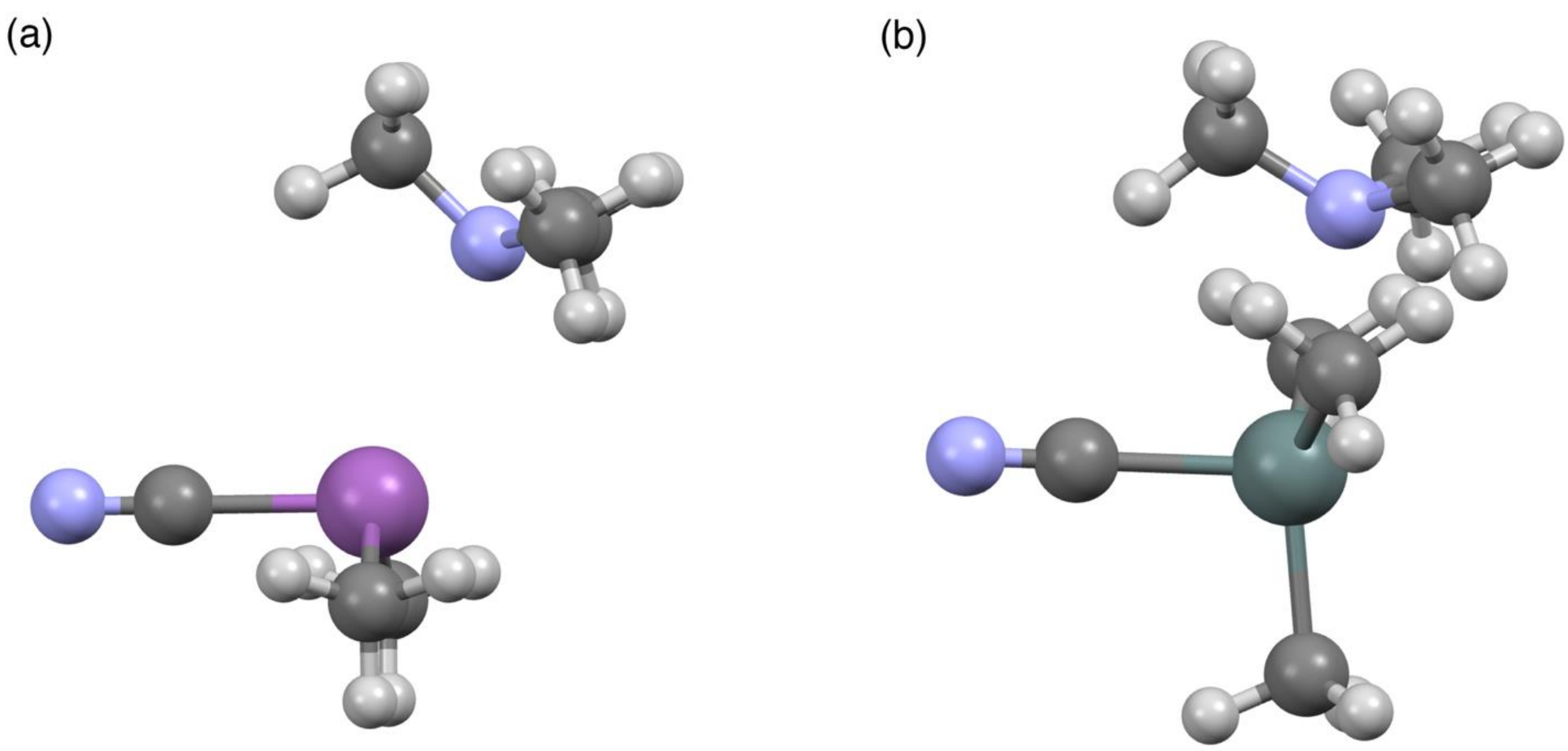

Figure S41. Geometries corresponding to the approximate maxima in the total SAPT2 interaction energy scans as a function of the σ-hole angle (θ_σ), shown in Figures 15 and S39. (a) The pnictogen bonding in Me_2(NC)Sb···NMe_3 with θ_σ = 120°, where the nitrogen lone pair of NMe_3 approximately aligns with the Sb lone pair. The tetrel bonding in Me_3(NC)Sn···NMe_3 with θ_σ = 100°, inducing steric clash of methyl groups on σ-hole donor and acceptor.

S6. SAPT Lone Pair Angle Scans

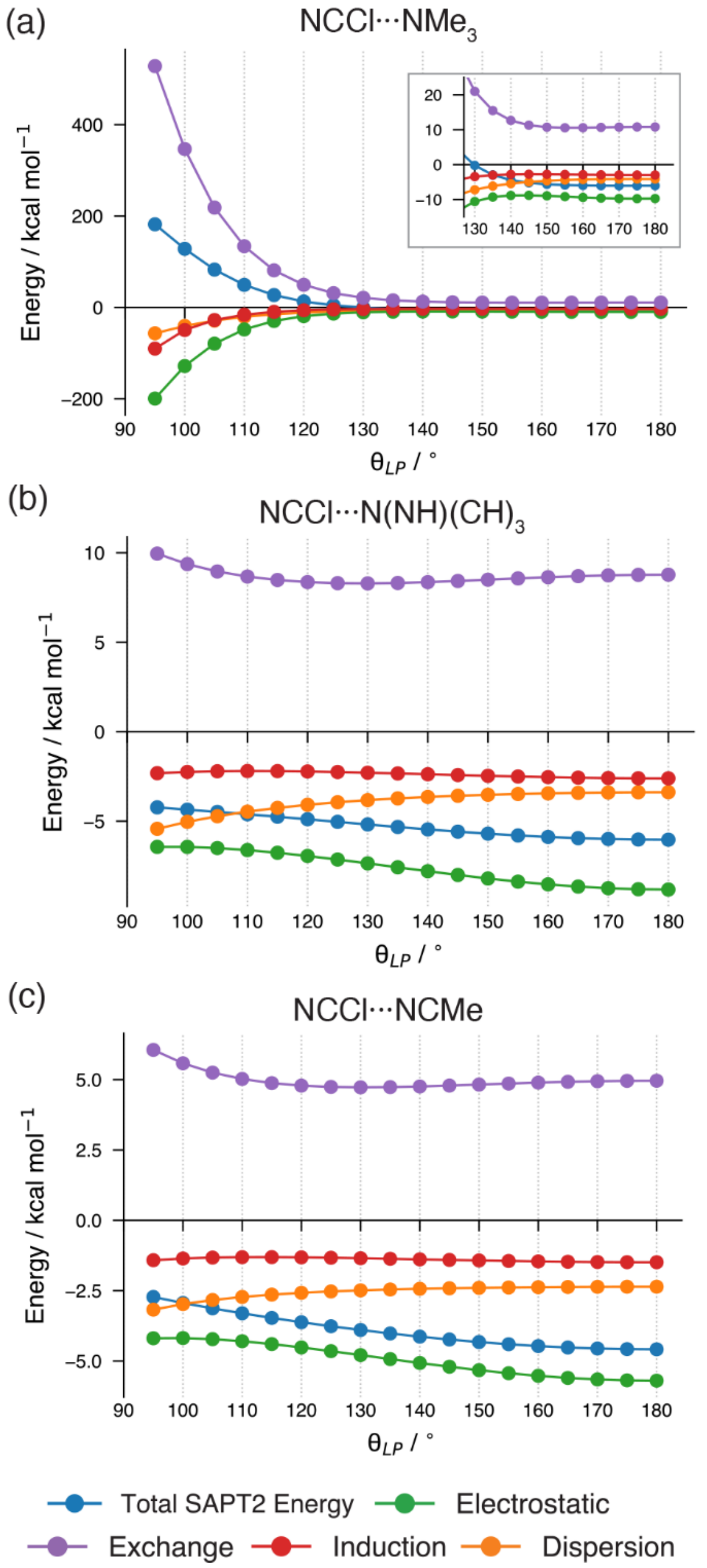

Figure S42. SAPT2/def2-TZVPD energy components as a function of the lone-pair angle (θ_{LP}) for (a) halogen bonding between ClCN and NMe_3, (b) halogen bonding between ClCN and imidazole, and (c) halogen bonding between ClCN and MeCN. The total SAPT2 interaction energy is abbreviated as "Total SAPT2 Energy". The inset to panel (a) shows the change in the energy components over the range θ_{LP} = 130 – 180°.

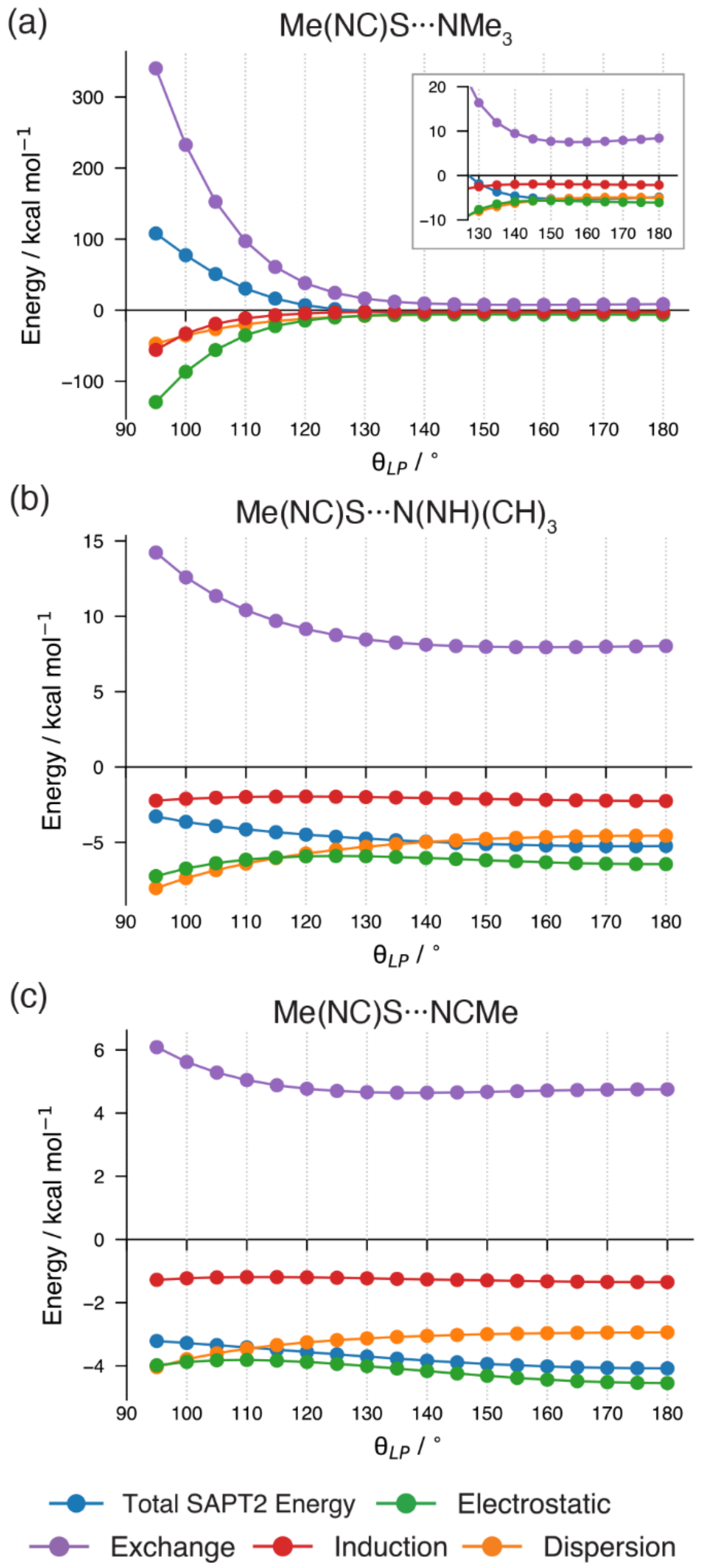

Figure S43. SAPT2/def2-TZVPD energy components as a function of the lone-pair angle (θ_{LP}) for (a) chalcogen bonding between S(CN)Me and NMe_3, (b) chalcogen bonding between S(CN)Me and imidazole, and (c) chalcogen bonding between S(CN)Me and MeCN. The total SAPT2 interaction energy is abbreviated as "Total SAPT2 Energy". The inset to panel (a) shows the change in the energy components over the range θ_{LP} = 130 – 180°.

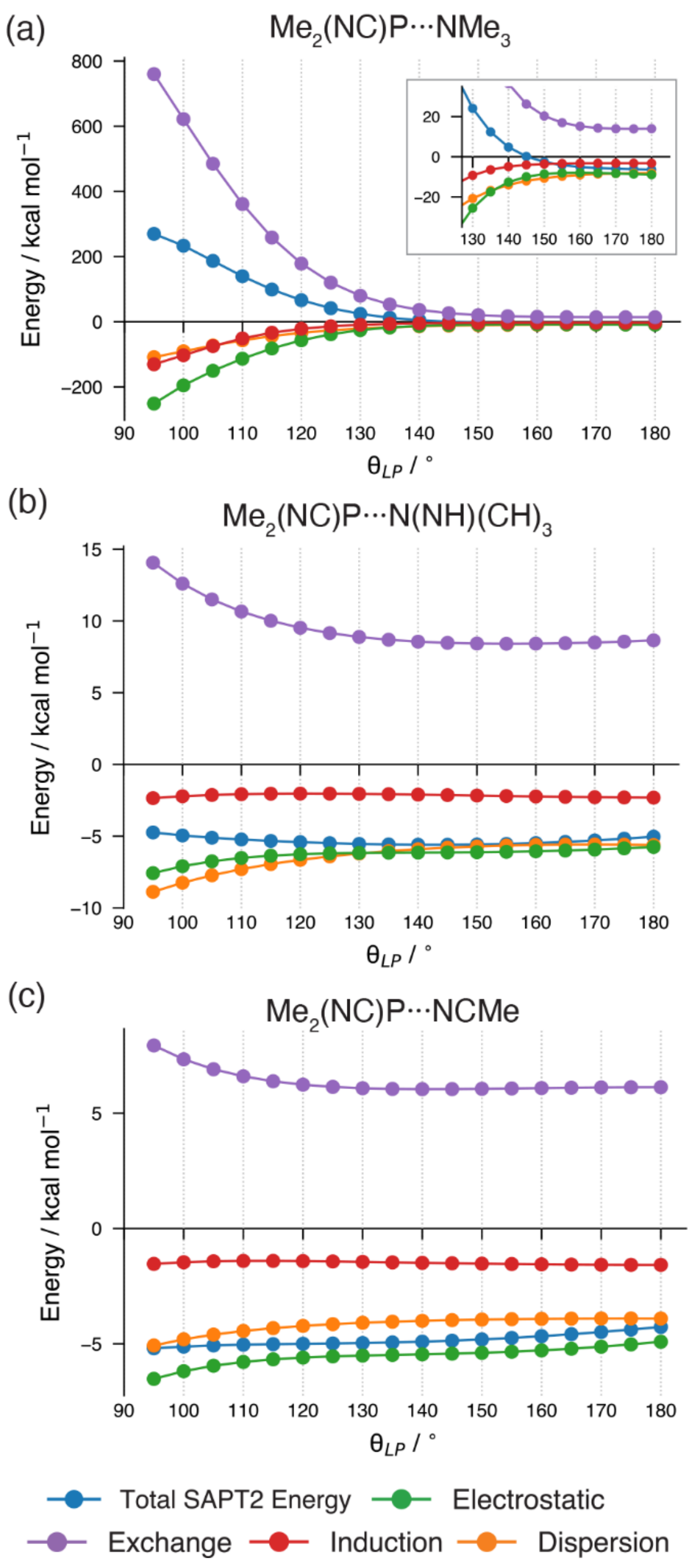

Figure S44. SAPT2/def2-TZVPD energy components as a function of the lone-pair angle (θ_{LP}) for (a) pnictogen bonding between $P(CN)Me_2$ and NMe_3, (b) pnictogen bonding between $P(CN)Me_2$ and imidazole, and (c) pnictogen bonding between $P(CN)Me_2$ and MeCN. The total SAPT2 interaction energy is abbreviated as "Total SAPT2 Energy". The inset to panel (a) shows the change in the energy components over the range θ_{LP} = 130 – 180°.

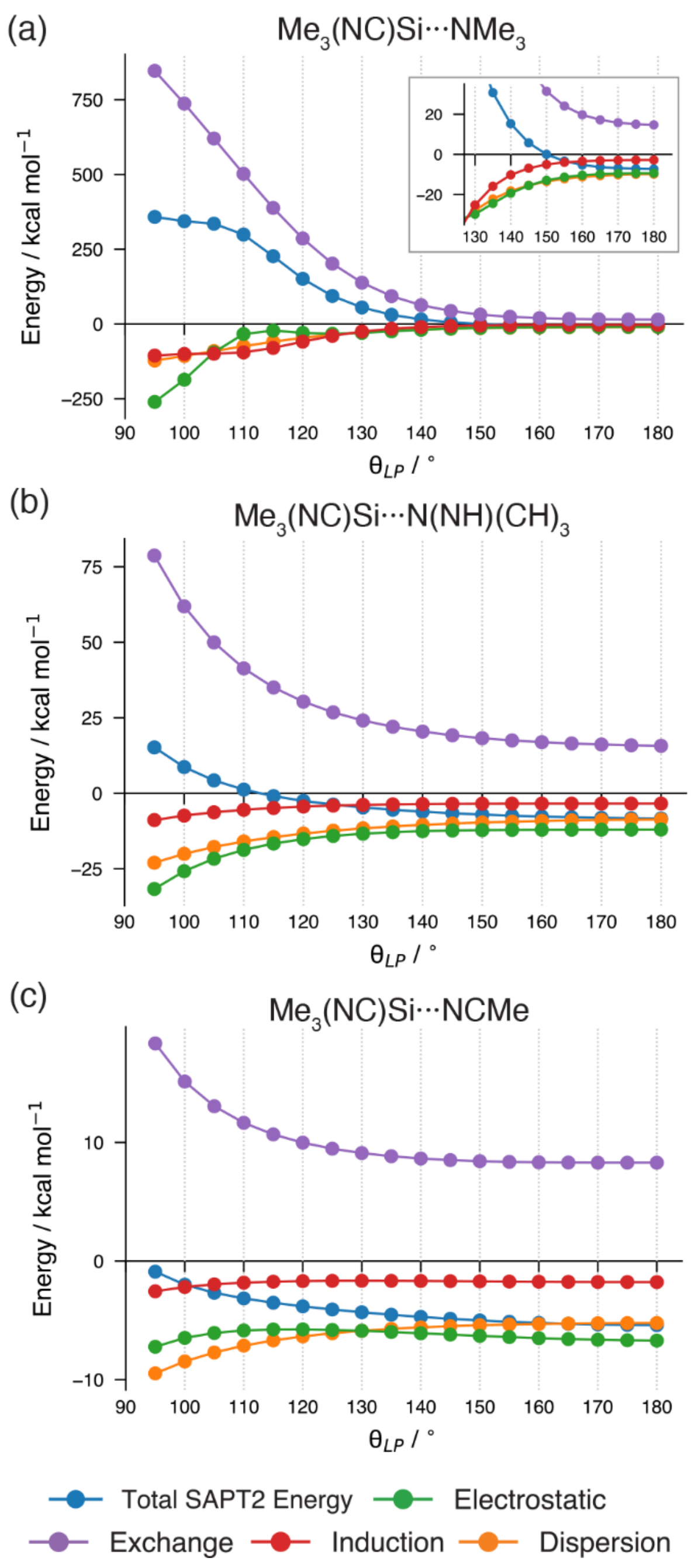

Figure S45. SAPT2/def2-TZVPD energy components as a function of the lone-pair angle (θ_{LP}) for (a) tetrel bonding between $Si(CN)Me_3$ and NMe_3, (b) tetrel bonding between $Si(CN)Me_3$ and imidazole, and (c) tetrel bonding between $Si(CN)Me_3$ and MeCN. The total SAPT2 interaction energy is abbreviated as "Total SAPT2 Energy". The inset to panel (a) shows the change in the energy components over the range θ_{LP} = 130 – 180°.

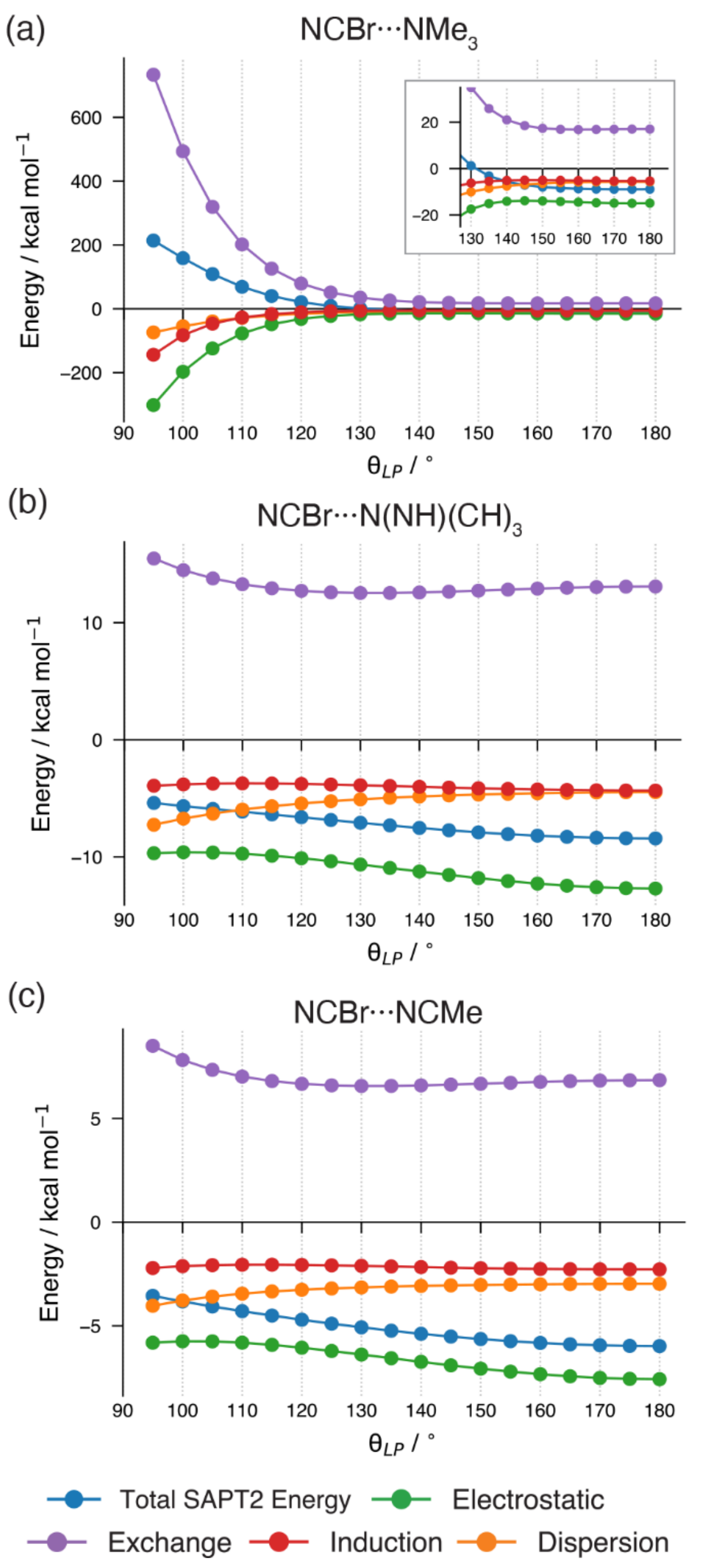

Figure S46. SAPT2/def2-TZVPD energy components as a function of the lone-pair angle (θ_{LP}) for (a) halogen bonding between BrCN and NMe_3, (b) halogen bonding between BrCN and imidazole, and (c) halogen bonding between BrCN and MeCN. The total SAPT2 interaction energy is abbreviated as "Total SAPT2 Energy". The inset to panel (a) shows the change in the energy components over the range θ_{LP} = 130 – 180°.

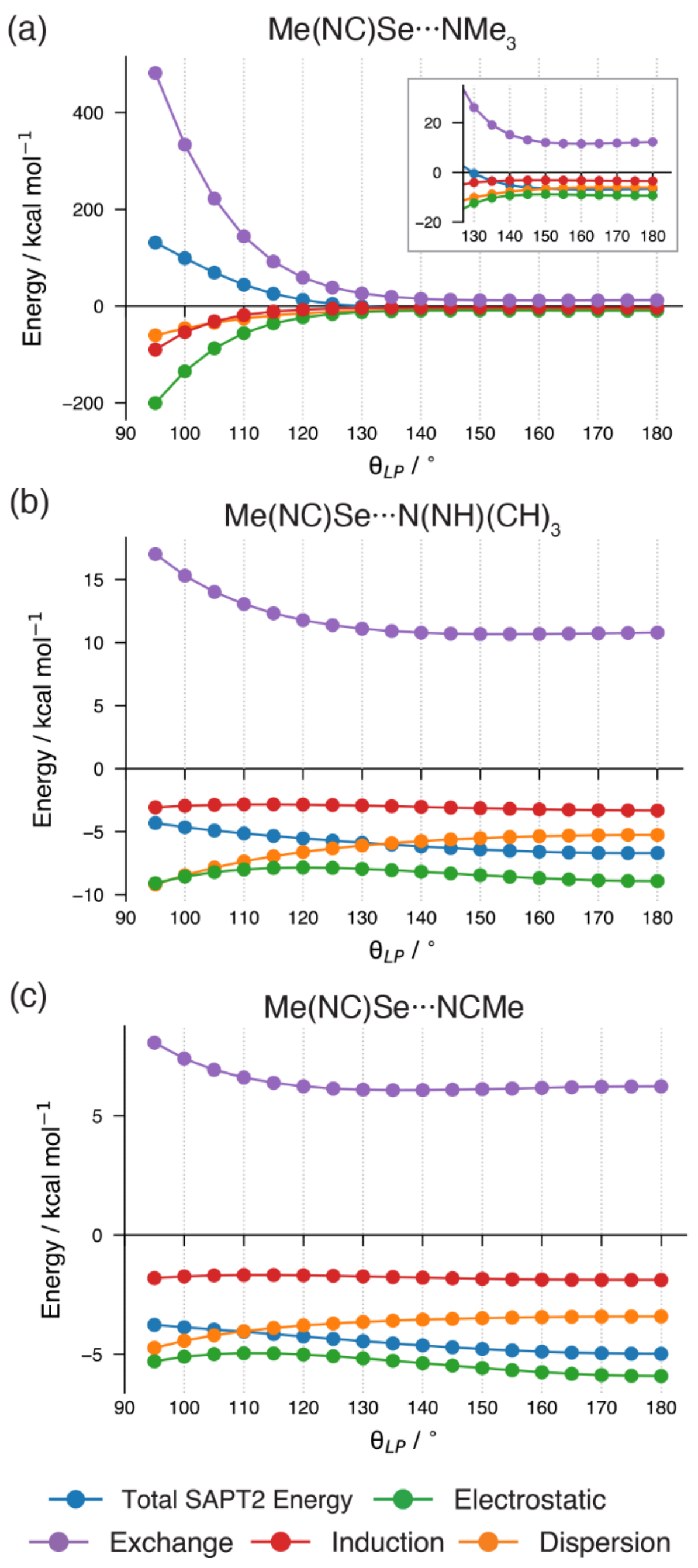

Figure S47. SAPT2/def2-TZVPD energy components as a function of the lone-pair angle (θ_{LP}) for (a) chalcogen bonding between Se(CN)Me and NMe_3, (b) chalcogen bonding between Se(CN)Me and imidazole, and (c) chalcogen bonding between Se(CN)Me and MeCN. The total SAPT2 interaction energy is abbreviated as "Total SAPT2 Energy". The inset to panel (a) shows the change in the energy components over the range θ_{LP} = 130 – 180°.

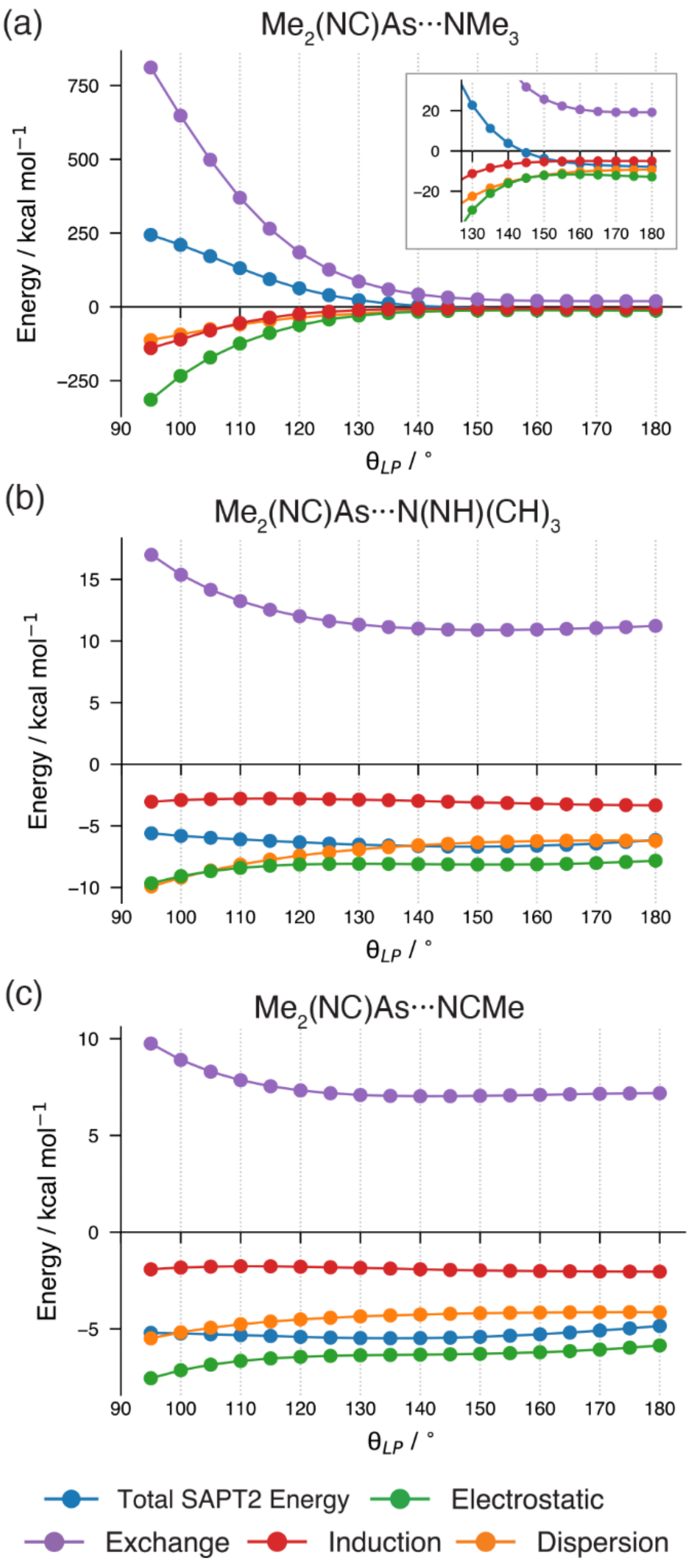

Figure S48 . SAPT2/def2-TZVPD energy components as a function of the lone-pair angle (θ_{LP}) for (a) pnictogen bonding between As(CN)Me_2 and NMe_3, (b) pnictogen bonding between As(CN)Me_2 and imidazole, and (c) pnictogen bonding between As(CN)Me_2 and MeCN. The total SAPT2 interaction energy is abbreviated as "Total SAPT2 Energy". The inset to panel (a) shows the change in the energy components over the range θ_{LP} = 130 – 180°.

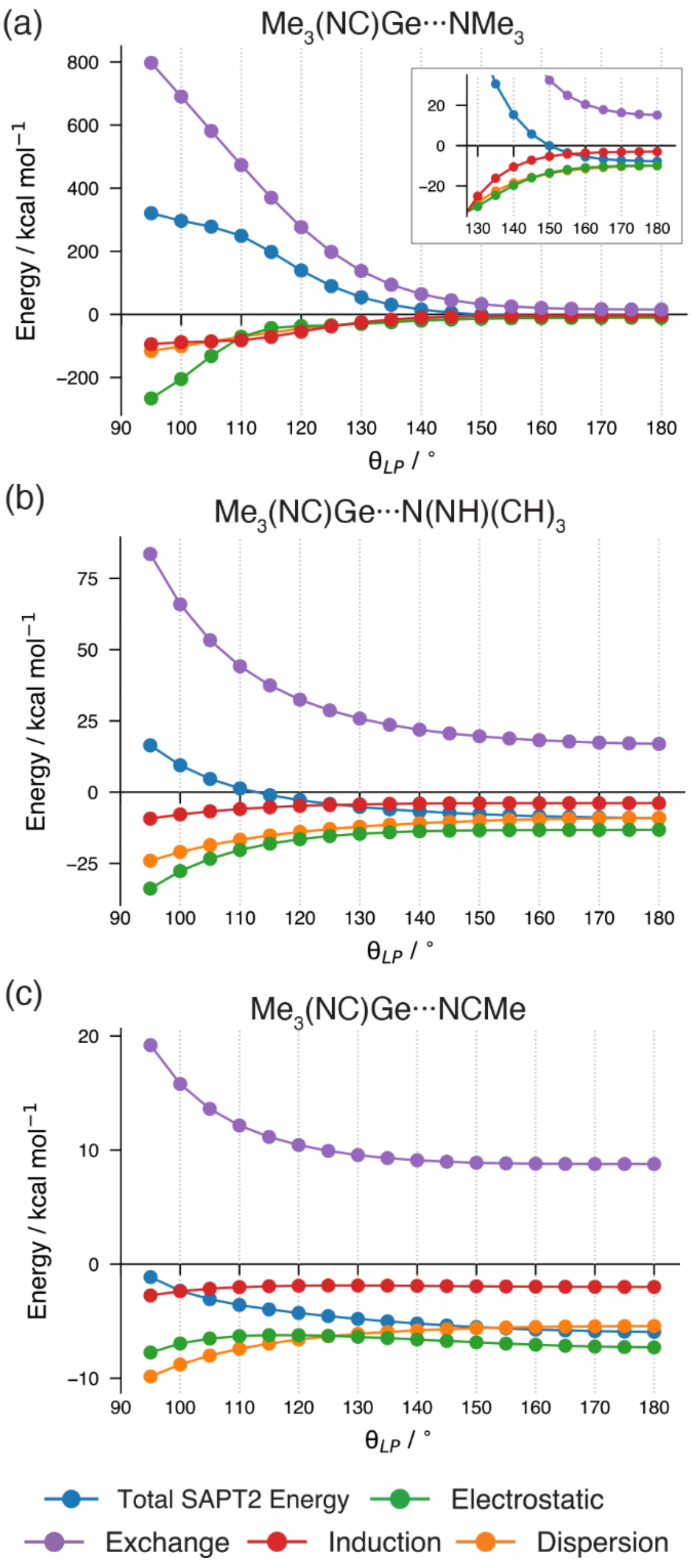

Figure S49. SAPT2/def2-TZVPD energy components as a function of the lone-pair angle (θ_{LP}) for (a) tetrel bonding between Ge(CN)$Me_3$and NMe_3, (b) tetrel bonding between Ge(CN)Me_3 and imidazole, and (c) tetrel bonding between Ge(CN)Me_3 and MeCN. The total SAPT2 interaction energy is abbreviated as "Total SAPT2 Energy". The inset to panel (a) shows the change in the energy components over the range θ_{LP} = 130 – 180°.

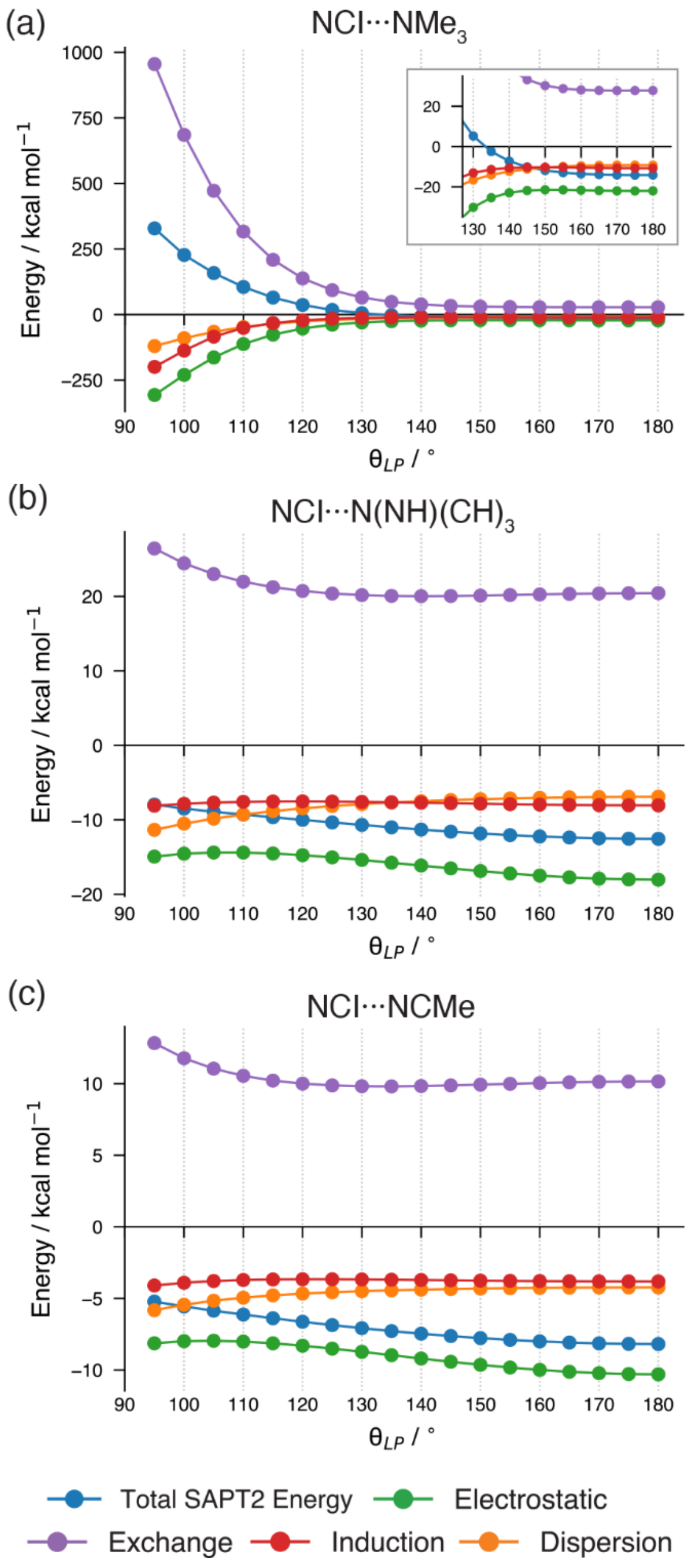

Figure S50. SAPT2/def2-TZVPD energy components as a function of the lone-pair angle (θ_{LP}) for (a) halogen bonding between ICN and NMe_3, (b) halogen bonding between ICN and imidazole, and (c) halogen bonding between ICN and MeCN. The total SAPT2 interaction energy is abbreviated as “Total SAPT2 Energy”. The inset to panel (a) shows the change in the energy components over the range θ_{LP} = 130 – 180°.

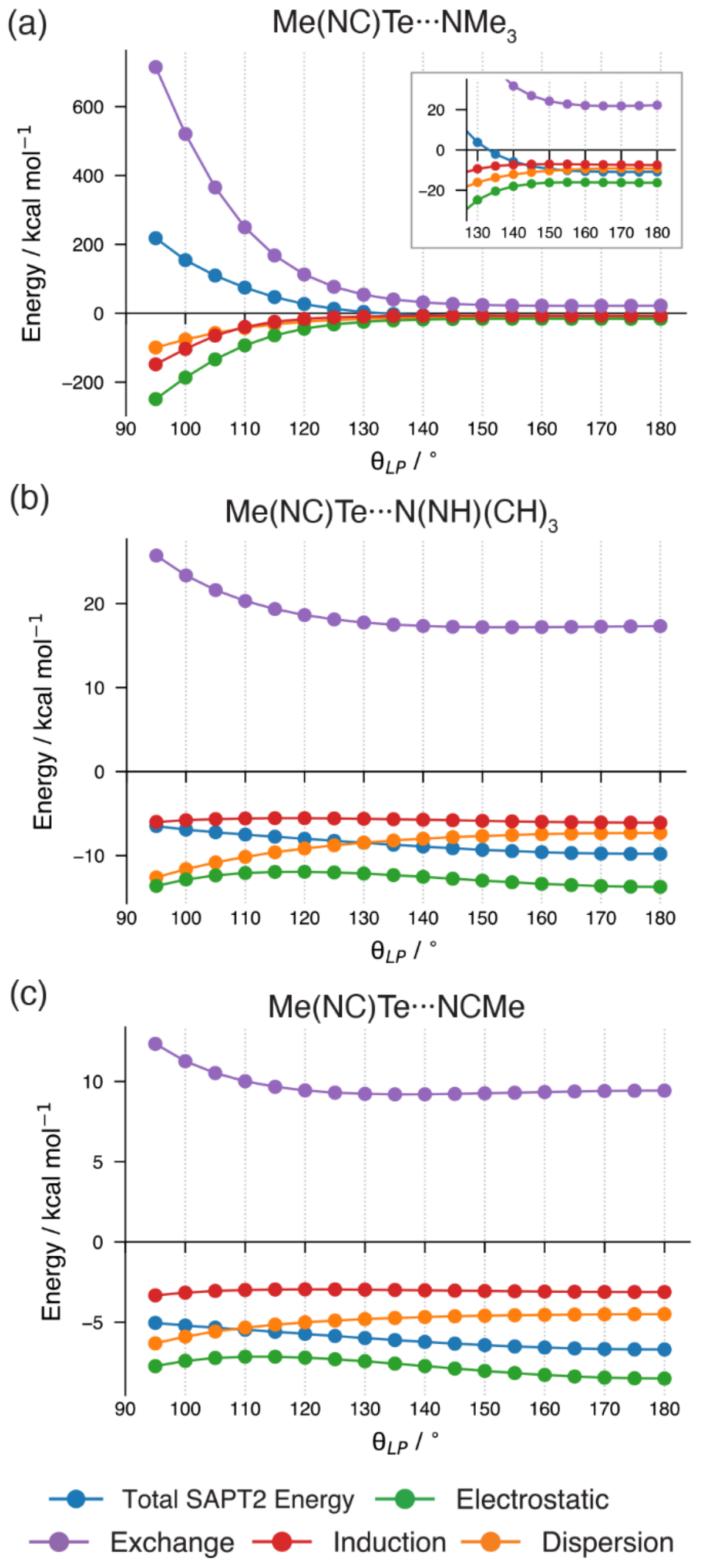

Figure S51. SAPT2/def2-TZVPD energy components as a function of the lone-pair angle (θ_{LP}) for (a) chalcogen bonding between Te(CN)Me and NMe_3, (b) chalcogen bonding between Te(CN)Me and imidazole, and (c) chalcogen bonding between Te(CN)Me and MeCN. The total SAPT2 interaction energy is abbreviated as “Total SAPT2 Energy”. The inset to panel (a) shows the change in the energy components over the range θ_{LP} = 130 – 180°.

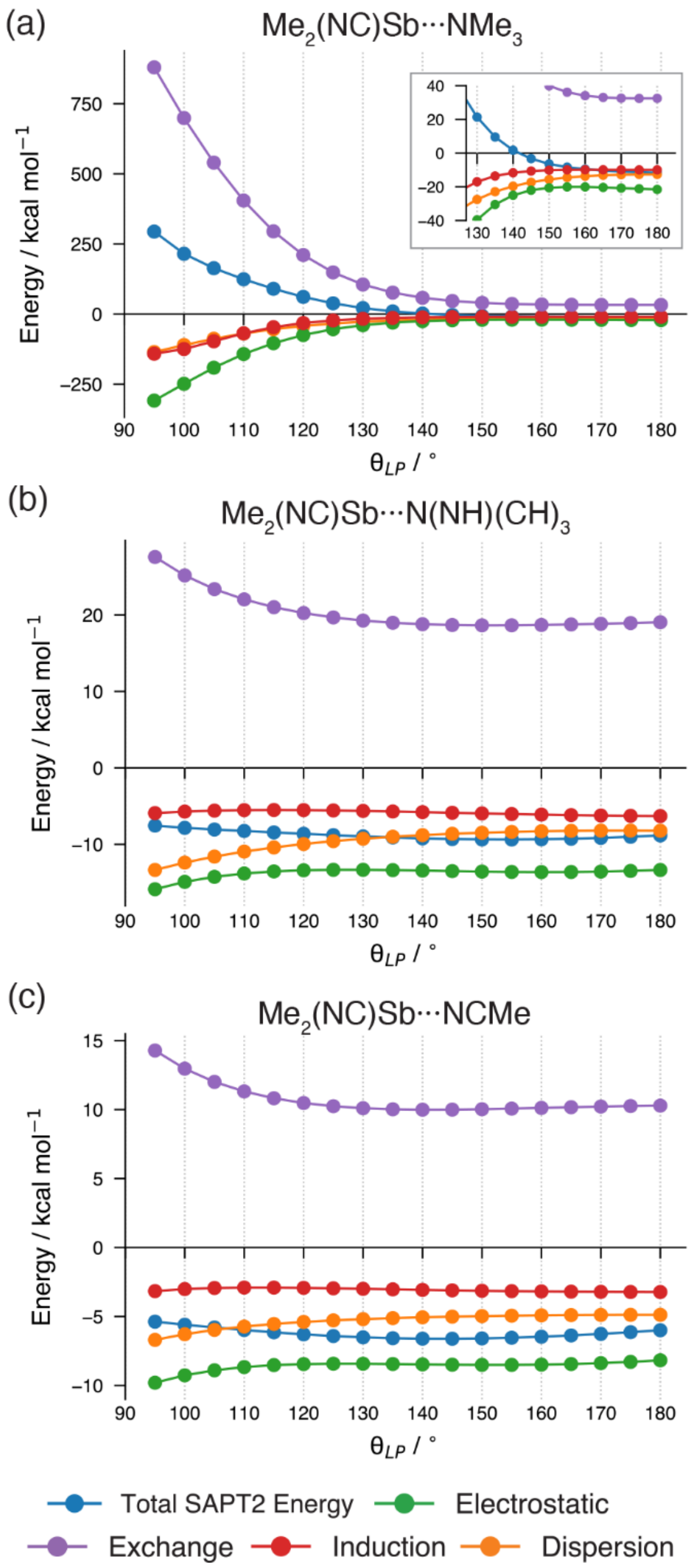

Figure S52. SAPT2/def2-TZVPD energy components as a function of the lone-pair angle (θ_{LP}) for (a) pnictogen bonding between $Sb(CN)Me_2$ and NMe_3, (b) pnictogen bonding between $Sb(CN)Me_2$ and imidazole, and (c) pnictogen bonding between $Sb(CN)Me_2$ and MeCN. The total SAPT2 interaction energy is abbreviated as "Total SAPT2 Energy". The inset to panel (a) shows the change in the energy components over the range θ_{LP} = 130 – 180°.

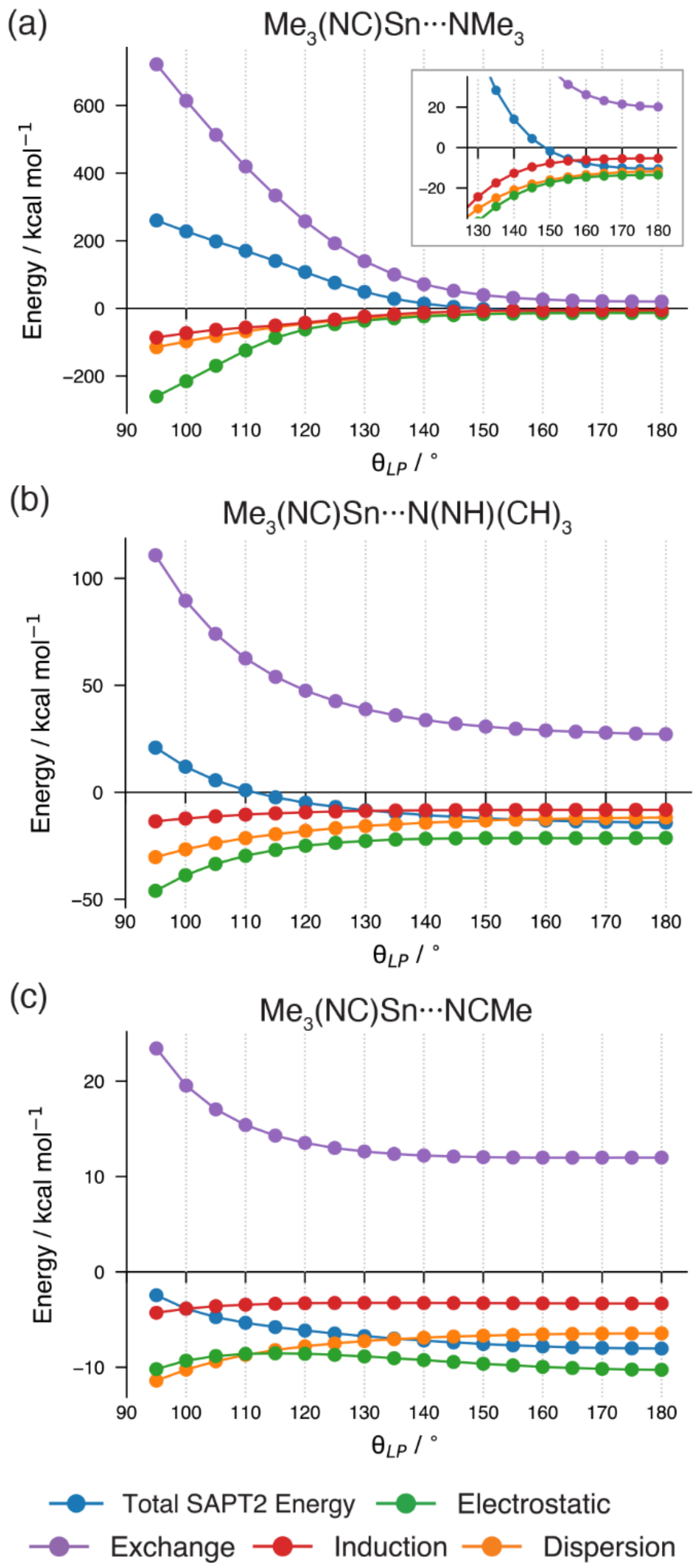

Figure S53. SAPT2/def2-TZVPD energy components as a function of the lone-pair angle (θ_{LP}) for (a) tetrel bonding between $Sn(CN)Me_3$ and NMe_3, (b) tetrel bonding between $Sn(CN)Me_3$ and imidazole, and (c) tetrel bonding between $Sn(CN)Me_3$ and MeCN. The total SAPT2 interaction energy is abbreviated as "Total SAPT2 Energy". The inset to panel (a) shows the change in the energy components over the range θ_{LP} = 130 – 180°.

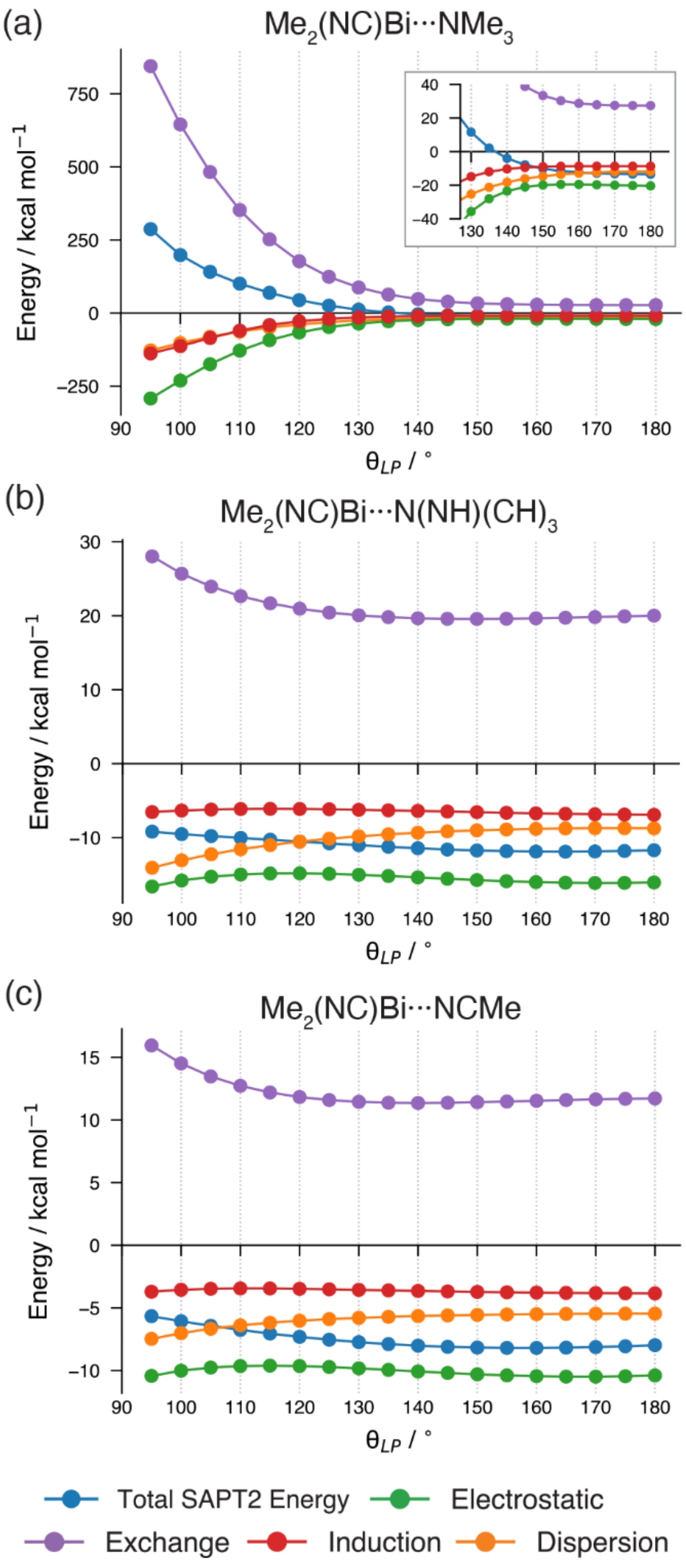

Figure S54. SAPT2/def2-TZVPD energy components as a function of the lone-pair angle (θ_{LP}) for (a) pnictogen bonding between $Bi(CN)Me_2$ and NMe_3, (b) pnictogen bonding between $Bi(CN)Me_2$ and imidazole, and (c) pnictogen bonding between $Bi(CN)Me_2$ and MeCN. The total SAPT2 interaction energy is abbreviated as "Total SAPT2 Energy". The inset to panel (a) shows the change in the energy components over the range θ_{LP} = 130 – 180°.

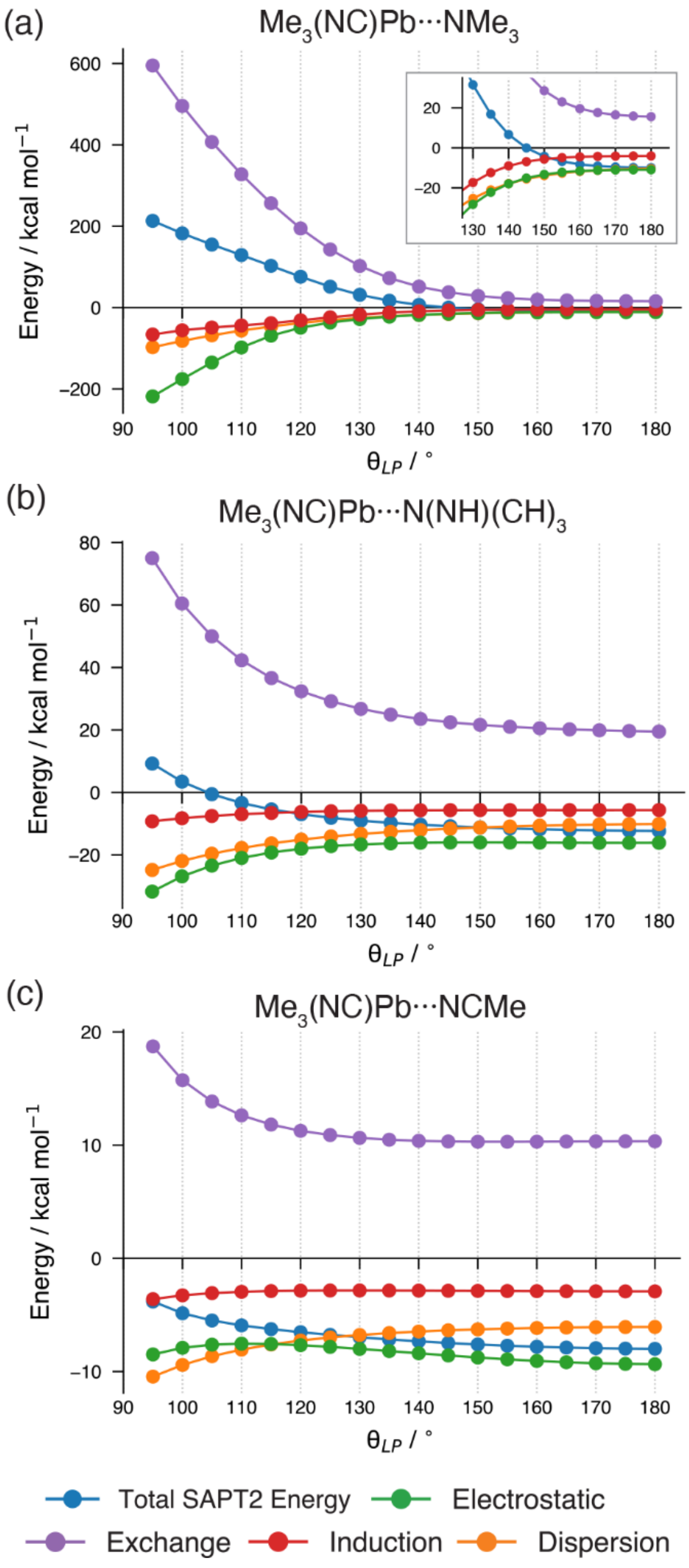

Figure S55. SAPT2/def2-TZVPD energy components as a function of the lone-pair angle (θ_{LP}) for (a) tetrel bonding between $Pb(CN)Me_3$ and NMe_3, (b) tetrel bonding between $Pb(CN)Me_3$ and imidazole, and (c) tetrel bonding between $Pb(CN)Me_3$ and MeCN. The total SAPT2 interaction energy is abbreviated as "Total SAPT2 Energy". The inset to panel (a) shows the change in the energy components over the range θ_{LP} = 130 – 180°.

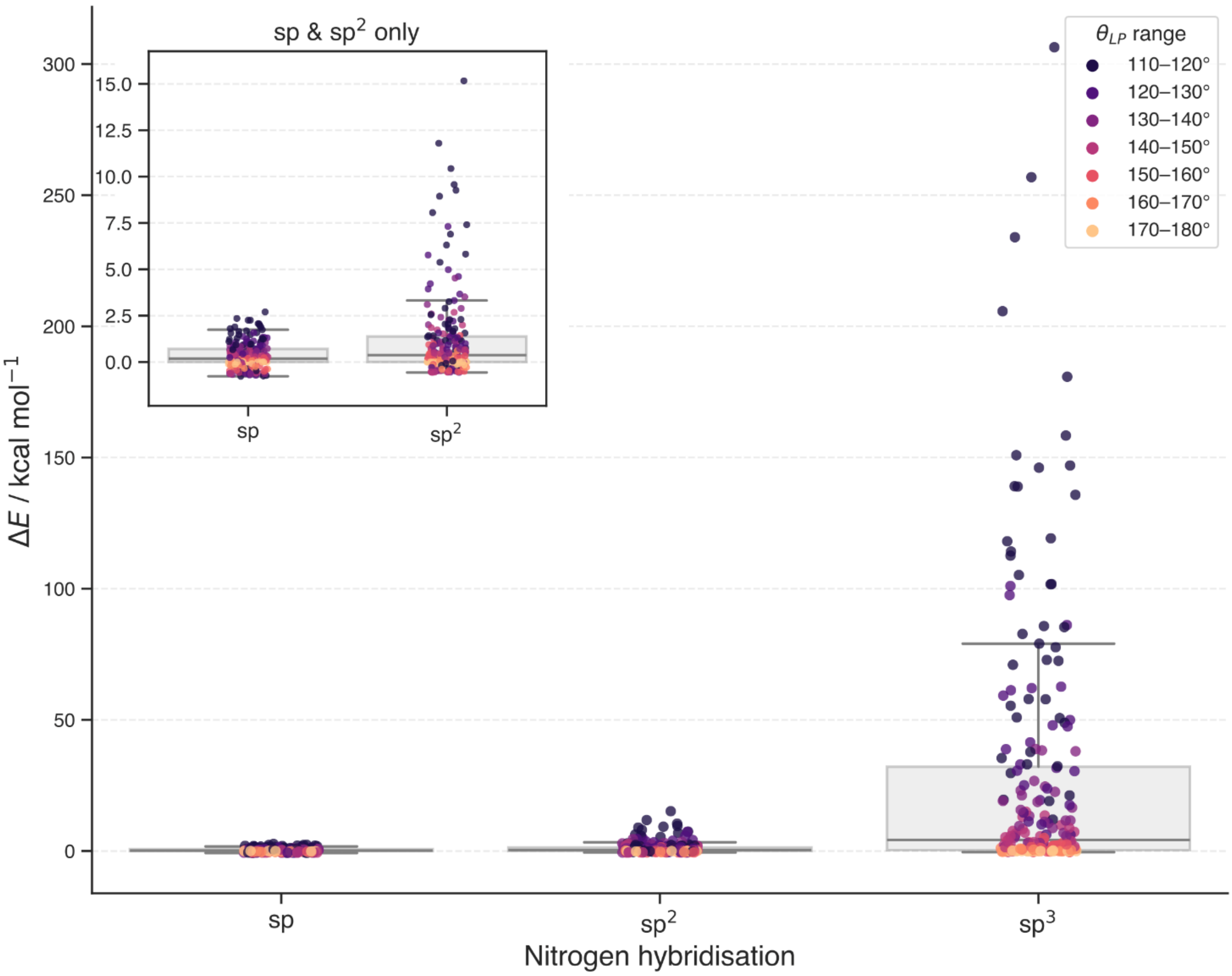

Figure S56. Box and strip plots illustrating the range of total interaction energies as a function of lone-pair angle (θ_{LP}) for halogen bonds involving ICN as halogen bond donor and the acceptors MeCN (N_{sp}), imidazole (N_{sp^2}) and NMe_3 (N_{sp^3}). Energies for a given θ_{LP} value are presented relative to the energy at θ_{LP} = 180°. Thus, $\Delta E = E(\theta_{LP}) - E(180°)$.

S7. References

S1. Groom, C. R.; Bruno, I. J.; Lightfoot, M. P.; Ward, S. C. The Cambridge Structural Database. *Acta Crystallogr.* **2016**, B*72*, 171−179.

S2. Bruno, I. J.; Cole, J. C.; Edgington, P. R.; Kessler, M.; Macrae, C. F.; McCabe, P.; Pearson, J.; Taylor, R. New Software for Searching the Cambridge Structural Database and Visualising Crystal Structures. *Acta Crystallogr.* **2002**, B58, 389−397.

S3. Sykes, R. A.; Johnson, N. T.; Kingsbury, C. J.; Harter, J.; Maloney, A. G. P.; Sugden, I. J.; Ward, S. C.; Bruno, I. J.; Adcock, S. A.; Wood, P. A.; McCabe, P.; Moldovan, A. A.; Atkinson, F.; Giangreco, I.; Cole, J. C. What has scripting ever done for us? The CSD Python application programming interface (API). *J. Appl. Cryst.* **2024**, *57*, 1235-1250.

S4. Alvarez, S. A Cartography of the van der Waals Territories. *Dalton Trans.* **2013**, *42*, 8617−8636.

S5. Allen, F. H.; Motherwell, W. D. S.; Raithby, P. R.; Shields, G. P.; Taylor, R. Systematic analysis of the probabilities of formation of bimolecular hydrogen-bonded ring motifs in organic crystal structures. *New J. Chem.* **1999**, *23*, 25-34.

S6. Zhao, Y.; Truhlar, D. G. The M06 suite of density functionals for main group thermochemistry, thermochemical kinetics, noncovalent interactions, excited states, and transition elements: two new functionals and systematic testing of four M06-class functionals and 12 other functionals. *Theor. Chem. Account.* **2008**, *120*, 215-241.

S7. Grimme, S.; Antony, J.; Ehrlich, S.; Krieg, H. A consistent and accurate ab initio parameterization of density functional dispersion correction (DFT-D) for the 94 elements H-Pu. *J. Chem. Phys*. **2010**, *132*, 154104.
S8. Weigend, F.; Ahlrichs, R. Balanced basis sets of split valence, triple zeta valence and quadruple zeta valence quality for H to Rn: Design and assessment of accuracy. *Phys. Chem. Chem. Phys*. **2005**, *7*, 3297–3305.
S9. Rappoport, D.; Furche, F.; Property-optimized Gaussian basis sets for molecular response calculations. *J. Chem. Phys*. **2010**, *133*, 134105.
S10. Neese, F.; Software update: The ORCA program system – Version 5.0. *Wiley Interdiscip. Rev. Comput. Mol. Sci.* **2022**, *12*, e1606.
S11. Werner, H.-J.; Knowles, P. J.; Manby, F. R.; Black J. A.; Doll, K.; Hesselmann, A.; Kats, D.; Köhn, A.; Korona, T.; Kreplin, D. A.; Ma, Q.; Miller III, T. F.; Mitrushenkov, A.; Peterson, K. A.; Polyak, I.; Rauhut, G.; Sibaev, M. The Molpro quantum chemistry package. *J. Chem. Phys*. **2020**, *152*, 144107.
S12. Werner, H.-J.; Knowles, P. J.; Celani, P.; Györffy, W.; Hesselmann, A.; Kats, D.; Knizia, G.; Köhn, A.; Korona, T.; Kreplin, D.; Lindh, R.; Ma, Q.; Manby, F. R.; Mitrushenkov, A.; Rauhut, G.; Schütz, M.; Shamasundar, K. R.; Adler, T. B.; Amos, R. D.; Bennie, S. J.; Bernhardsson, A.; Berning, A.; Black, J. A.; Bygrave, P. J.; Cimiraglia, R.; Cooper, D. L.; Coughtrie, D.; Deegan, M. J. O.; Dobbyn, A. J.; Doll, K.; Dornbach, M.; Eckert, F.; Erfort, S.; Goll, E.; Hampel, C.; Hetzer, G.; Hill, J. G.; Hodges, M.; Hrenar, T.; Jansen, G.; Köppl, C.; Kollmar, C.; Lee, S. J. R.; Liu, Y.; Lloyd, A. W.; Mata, R. A.; May, A. J.; Mussard, B.; McNicholas, S. J.; Meyer, W.; Miller III, T. F.; Mura, M. E.; Nicklass, A.; O'Neill, D. P.; Palmieri, P.; Peng, D.; Peterson, K. A.; Pflüger, K.; Pitzer, R.; Polyak, I.; Reiher, M.; Richardson, J. O.; Robinson, J. B.; Schröder, B.; Schwilk, M.; Shiozaki, T.; Sibaev, M.; Stoll, H.; Stone, A, J.; Tarroni, R.; Thorsteinsson, T.; Toulouse, J.; Wang, M.; Welborn, M.; Ziegler, B. MOLPRO, version 2023.2, a package of ab initio programs, 2023, see http://www.molpro.net.
S13. K. Kříž and J. Řezáč, Non-covalent interactions atlas benchmark data sets 4: σ-hole interactions. *Phys. Chem. Chem. Phys.*, **2022**, *24*, 14794–14804.
S14. Ma, Q.; Schwilk, M.; Christoph Köppl; Werner, H.-J. Scalable Electron Correlation Methods. 4. Parallel Explicitly Correlated Local Coupled Cluster with Pair Natural Orbitals (PNO-LCCSD-F12). *J. Chem. Theory Comput.* **2017**, *13*, 4871–4896.
S15. Ma, Q.; Werner, H. Explicitly Correlated Local Coupled-Cluster Methods Using Pair Natural Orbitals. *Wiley Interdiscip. Rev. Comput. Mol. Sci.* **2018**, *8*, e1371.
S16. Ma, Q.; Werner, H.-J. Scalable Electron Correlation Methods. 5. Parallel Perturbative Triples Correction for Explicitly Correlated Local Coupled Cluster with Pair Natural Orbitals. *J. Chem. Theory Comput.* **2017**, *14* (1), 198–215.
S17. Peterson, K. A.; Adler, T.; Werner, H.-J. Systematically Convergent Basis Sets for Explicitly Correlated Wavefunctions: The Atoms H, He, B–Ne, and Al–Ar. *J. Chem. Phys.* **2008**, *128*, 084102.
S18. Hill, J. G.; Peterson, K. A. Correlation consistent basis sets for explicitly correlated wavefunctions: Pseudopotential-based basis sets for the post-*d* main group elements Ga–Rn. *J. Chem. Phys*., **2014**, *141*, 094106.
S19. Knizia, G.; Adler, T. B.; Werner, H.-J. Simplified CCSD(T)-F12 Methods: Theory and Benchmarks. *J. Chem. Phys*., **2009**, *130*, 054104.
S20. Marchetti, O.; Werner, H.-J. Accurate Calculations of Intermolecular Interaction Energies Using Explicitly Correlated Coupled Cluster Wave Functions and a Dispersion-Weighted MP2 Method. *J. Phys. Chem. A.* **2009**, *113*, 11580–11585.
S21. Weigend, F. A fully direct RI-HF algorithm: Implementation, optimised auxiliary basis sets, demonstration of accuracy and efficiency, *Phys. Chem. Chem. Phys.*, **2002**, *4*, 4285–4291.
S22. Kritikou, S.; Hill, J. G. Auxiliary Basis Sets for Density Fitting in Explicitly Correlated Calculations: The Atoms H–Ar, *J. Chem. Theory Comput.*, **2015**, *11*, 5269–5276.
S23. Yousaf, K. E.; Peterson, K. A. Optimized auxiliary basis sets for explicitly correlated methods, *J. Chem. Phys*., **2008**, *129*, 184108.
S24. Weigend, F. Hartree–Fock Exchange Fitting Basis Sets for H to Rn. *J. Comput. Chem.* **2008**, *29*, 167–175.
S25. Shaw, R. A.; Hill, J. G. Approaching the Hartree–Fock Limit through the Complementary Auxiliary Basis Set Singles Correction and Auxiliary Basis Sets. *J. Chem. Theory Comput.* **2017**, *13*, 1691–1698.
S26. Valeev, E. F. Improving on the Resolution of the Identity in Linear R12 Ab Initio Theories. *Chem. Phys. Lett.* **2004**, *395*, 190–195.
S27. Werner, H.; Knizia, G.; Manby, F. R. Explicitly Correlated Coupled Cluster Methods with Pair-Specific Geminals. *Mol. Phys.* **2011**, *109*, 407–417.

S28. Peterson, K. A.; Krause, C.; Stoll, H.; Hill, J. G.; Werner, H.-J. Application of Explicitly Correlated Coupled-Cluster Methods to Molecules Containing Post-3*d* Main Group Elements. *Mol. Phys.* **2011**, *109*, 2607–2623.

S29. Jeziorski, B.; Moszynski, R.; Szalewicz, K. Perturbation Theory Approach to Intermolecular Potential Energy Surfaces of van der Waals Complexes. *Chem. Rev.* **1994**, *94*, 1887–1930.

S30. Smith, D. G. A.; Burns, L. A.; Simmonett, A. C.; Parrish, R. M.; Schieber, M. C.; Galvelis, R.; Kraus, P.; Kruse, H.; Di Remigio, R.; Alenaizan, A.; James, A. M.; Lehtola, S.; Misiewicz, J. P.; Scheurer, M.; Shaw, R. A.; Schriber, J. B.; Xie, Y.; Glick, Z. L.; Sirianni, D. A.; O'Brien, J. S. Psi4 1.4: Open-Source Software for High-Throughput Quantum Chemistry. *J. Chem. Phys*. **2020**, *152*, 184108.

S31. Polik, W. F.; Schmidt, J. R. WebMO : Web-Based Computational Chemistry Calculations in Education and Research. *Wiley Interdiscip. Rev. Comput. Mol. Sci.* **2022**, *12*, e1554.

S32. Frisch, M. J.; Trucks, G. W.; Schlegel, H. B.; Scuseria, G. E.; Robb, M. A.; Cheeseman, J. R.; Scalmani, G.; Barone, V.; Petersson, G. A.; Nakatsuji, H.; Li, X.; Caricato, M.; Marenich, A. V.; Bloino, J.; Janesko, B. G.; Gomperts, R.; Mennucci, B.; Hratchian, H. P.; Ortiz, J. V.; Izmaylov, A. F.; Sonnenberg, J. L.; Williams-Young, D.; Ding, F.; Lipparini, F.; Egidi, F.; Goings, J.; Peng, B.; Petrone, A.; Henderson, T.; Ranasinghe, D.; Zakrzewski, V. G.; Gao, J.; Rega, N.; Zheng, G.; Liang, W.; Hada, M.; Ehara, M.; Toyota, K.; Fukuda, R.; Hasegawa, J.; Ishida, M.; Nakajima, T.; Honda, Y.; Kitao, O.; Nakai, H.; Vreven, T.; Throssell, K.; Montgomery, J. A., Jr.; Peralta, J. E.; Ogliaro, F.; Bearpark, M. J.; Heyd, J. J.; Brothers, E. N.; Kudin, K. N.; Staroverov, V. N.; Keith, T. A.; Kobayashi, R.; Normand, J.; Raghavachari, K.; Rendell, A. P.; Burant, J. C.; Iyengar, S. S.; Tomasi, J.; Cossi, M.; Millam, J. M.; Klene, M.; Adamo, C.; Cammi, R.; Ochterski, J. W.; Martin, R. L.; Morokuma, K.; Farkas, O.; Foresman, J. B.; Fox, D. J. *Gaussian 16*, Revision C.01; Gaussian, Inc.: Wallingford, CT, 2016.

S33. Godbout, N.; Salahub, D. R.; Andzelm, J.; Wimmer, E. Optimization of Gaussian-Type Basis Sets for Local Spin Density Functional Calculations. Part I. Boron through Neon, Optimization Technique and Validation. *Can. J. Chem.* **1992**, *70*, 560–571.

S34. Gao, Y.; Chen, Y.; Li, R.; Bian, Y.; Li, X.; Jiang, J. Nonperipherally Octa(butyloxy)-Substituted Phthalocyanine Derivatives with Good Crystallinity: Effects of Metal-Ligand Coordination on the Molecular Structure, Internal Structure, and Dimensions of Self-Assembled Nanostructures. *Chem. Eur. J.* **2009**, *15*, 13241–13252.

S35. Yuan, Y.-Z.; Zhou, J.; Liu, X.; Liu, L.-H.; Yu, K.-B. A new hemidirected lead(II) complex converted into holodirected by secondary Pb-N interactions. *Inorg. Chem. Commun.* **2007**, *10*, 475–478.
